# Hierarchically Porous Carbons from Almond Residues via Hydrothermal Pretreatment and Mild K_2_CO_3_ Activation for Aqueous Zinc Hybrid Supercapacitors

**DOI:** 10.1021/acs.energyfuels.6c00654

**Published:** 2026-04-16

**Authors:** Densa A. Shaj, Darío Alvira, Daniel Antorán, Víctor Sebastián, Joan J. Manyà

**Affiliations:** † Aragón Institute for Engineering Research (I3A), Thermochemical Processes Group, 16765 University of Zaragoza, Escuela Politécnica Superior, Crta. de Cuarte s/n, 22071 Huesca, Spain; ‡ Department of Chemical Engineering and Environmental Technologies, University of Zaragoza, Campus Río Ebro, María de Luna 3, 50018 Zaragoza, Spain; § Instituto de Nanociencia y Materiales de Aragón (INMA), CSIC-Universidad de Zaragoza, 50009 Zaragoza, Spain; ∥ Networking Research Center on Bioengineering Biomaterials and Nanomedicine (CIBER-BBN), 28029 Madrid, Spain; ⊥ Laboratorio de Microscopías Avanzadas, Universidad de Zaragoza, 50018 Zaragoza, Spain

## Abstract

Agricultural residues offer a scalable feedstock for sustainable carbon electrodes, yet achieving high electrochemical performance in aqueous zinc-ion hybrid supercapacitors (ZHSCs) often relies on harsh activating agents and low carbon yields. Here, almond-tree pruning residues (AT) and almond shells (AS) are converted into porous carbons via hydrothermal pretreatment (HTC) followed by mild K_2_CO_3_ activation, enabling hierarchical porosity while limiting excessive burnoff. The HTC-assisted route markedly enhances N_2_-accessible surface area and mesopore volume, improving electrolyte accessibility and ion-transport pathways, while the presence of oxygen-containing groups contributes to favorable interfacial interactions in aqueous media. AT-derived carbons consistently outperform AS counterparts, highlighting the strong influence of precursor architecture on activation efficiency and pore connectivity. In a two-electrode aqueous ZHSC configuration (Zn metal anode; porous carbon cathode), the best performing AT-derived electrode delivered a specific capacity of 142 mAh g^–1^ at 0.1 A g^–1^ with 91% capacity retention after 10,000 cycles at 10 A g^–1^. Electrolyte chemistry plays a key role in durability: zinc trifluoromethanesulfonate (ZTFS) provides higher capacity retention and improved reversibility than ZnSO_4_, consistent with a more uniform Zn deposition and the formation of a less crystalline, fluorine-containing interphase, as evidenced by post-mortem analyses. Electrochemical impedance spectroscopy and galvanostatic intermittent titration techniques further support faster interfacial kinetics and more favorable transport in the best-performing carbon, in line with its balanced hierarchical porosity and surface chemistry. The device achieves an energy density of 87.8 Wh kg^–1^ at 62.3 W kg^–1^ and retains 37.9 Wh kg^–1^ at 13.6 kW kg^–1^, matching or surpassing many biomass-derived ZHSC cathodes prepared using more corrosive chemicals. Overall, this work demonstrates a greener, yield-efficient pathway to high-performance carbon cathodes for aqueous zinc-based hybrid energy storage.

## Graphical abstract

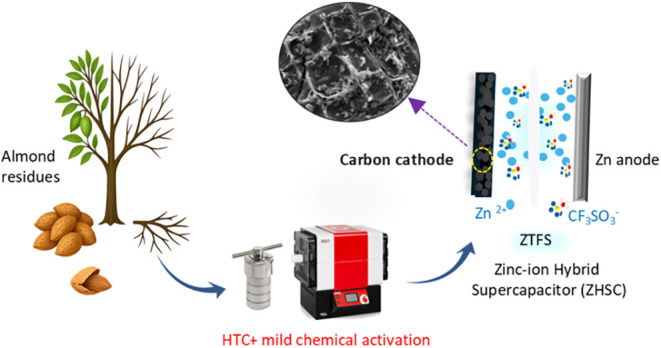

## Introduction

From the Voltaic cell to lithium-ion batteries and beyond, battery technology in energy storage devices has advanced steadily.^1^ However, this two-century-old legacy struggles to keep up with the rising energy demand, as it suffers from low power density, safety risks associated with organic electrolytes, high cost, and geopolitical concerns.^2^ By contrast, supercapacitors occupy the intermediate region between dielectric capacitors and batteries in the Ragone plot, delivering very high power (10^3^–10^5^ W kg^–1^) but typically at the expense of energy density.^3^ To address these limitations, hybrid supercapacitors (HSCs) have emerged as a promising direction due to their ability to bridge the performance gap between batteries and supercapacitors.^4^ At their core, HSCs synergistically combine faradaic and capacitive charge-storage mechanisms within a single system by employing distinct electrodes tailored to each function. Hence, this design integrates the advantages of both rechargeable batteries and supercapacitors, effectively mitigating the limitations typically encountered when either technology is used alone.^5^

Among metal-ion HSCs, alkali-based systems raise safety concerns because of the high reactivity of Li, Na, and K metals, as well as the flammability and volatility of organic electrolytes.^6^ In contrast, aqueous systems have attracted increasing attention due to their low cost, environmental friendliness, and improved intrinsic safety. In particular, zinc-ion hybrid supercapacitors (ZHSCs) are promising candidates for stable and efficient energy storage, thanks to the high ionic conductivity of aqueous electrolytes—enabling fast Zn^2+^ ion transport—together with broad electrode compatibility. A typical ZHSC couples a zinc metal-based anode with a porous carbon cathode in an aqueous zinc salt electrolyte—e.g., ZnSO_4_, Zn­(NO_3_)_2_, and Zn­(CF_3_SO_3_)_2_—with high ionic conductivity and low viscosity.^7^ The anode stores energy through reversible redox reactions, whereas the cathode operates via electrostatic interactions between its charged surface and electrolyte ions.^8^ Zinc is widely used as the anode owing to its low redox potential (−0.76 V vs SHE), excellent electrical conductivity, high theoretical gravimetric (823 mAh g^–1^) and volumetric (5855 mAh cm^–3^) capacities, low cost, nontoxicity, and well-established recycling infrastructure.^9^

In ZHSCs, carbon-based cathodes—such as porous carbon, graphene, and carbon nanotubes—feature high specific surface area, tunable porosity, good electrical conductivity, and superior chemical stability, which enable fast ion diffusion and outstanding cycling durability.^10^ In contrast, noncarbon-based materials (e.g., MXenes, transition-metal oxides, and conducting polymers) provide higher specific capacitance but often suffer from limited rate capability and structural degradation upon repeated cycling.^11^ Therefore, carbon-based electrodes are generally preferred for achieving operational stability over extended cycles and high power performance in ZHSCs.

The working principle of ZHSCs is governed by the reversible zinc plating/stripping reaction at the anode and the Zn^2+^ ion adsorption/desorption at the cathode. During charging, Zn^2+^ ions from the electrolyte migrate toward the zinc anode, gain electrons, and are deposited as metallic zinc. Conversely, during discharging, metallic zinc is oxidized, releasing electrons and dissolving back into the electrolyte as Zn^2+^ ions. At the carbon-based cathode, energy storage is predominantly governed by electrostatic interactions at the electrode–electrolyte interphase, following an electric double-layer capacitance (EDLC) mechanism.^7,12^ Heteroatom-containing surface groups may also influence the electrochemical response through additional surface redox contributions and improved electrode wettability.^13^ Although cation adsorption/desorption is generally considered the dominant process, anion adsorption/desorption has also been reported.^14,15^

Despite their promising working mechanism, ZHSCs still face key challenges that limit practical deployment. On the anode side, repeated zinc deposition/dissolution can lead to dendrite formation, dead zinc accumulation, and corrosion, decreasing the amount of active material and shortening cycle life.^16^ In addition, the electrochemical stability window of aqueous electrolytes is constrained by water decomposition, limiting operating voltage and energy density.^17^ On the cathode side, limited charge-storage capacity, sluggish ion transport, and side reactions can restrict the achievable energy and power densities of ZHSCs.^18^ Additionally, mismatched capacity and reaction kinetics between electrodes often lead to energy-density losses, especially under high-power operation.^3^ While extensive efforts have been made to stabilize the zinc anode, cathode design remains decisive for achieving high-performance ZHSCs. Its composition, porosity, and surface chemistry directly govern accessible adsorption sites, ion transport, and long-term stability.

Cathode performance is thereby closely linked to the density and accessibility of electrochemically active surface sites.^19^ Carbon materials commonly achieve higher surface area with tailored porosity and surface chemistry through chemical/physical activation,^20^ hard/soft templating methods,^21^ or heteroatom doping.^22^ These surface engineering strategies create hierarchical porous structures, improve electrolyte wettability and electrical conductivity, and thereby strengthen the double layer capacitive behavior. However, conventional chemical activation routes (e.g., KOH or ZnCl_2_) rely on corrosive and/or toxic reagents and harsh conditions, complicating scale-up and raise environmental and safety concerns.^23^ This has motivated interest in milder and more environmentally benign alternatives. In this context, K_2_CO_3_ is increasingly considered as a safer activating agent that can effectively generate predominantly microporous structures while preserving precursor morphology.^24^

Among carbon precursors, biomass-derived porous carbons (BCs) are especially attractive for practical cathodes because they are abundant, low-cost, structurally diverse, and tunable in surface chemistry, aligning with sustainability goals.^25^ Moreover, BCs can be produced from agroforestry residues, providing a route to valorize low-value waste within circular-economy approaches.^26^ In Mediterranean regions, almond agriculture is a particularly relevant source. Spain alone has 7 660 km^2^ of almond orchards and produced ca. 3.66 × 10^5^ Mg of in-shell almonds in the 2024–25 campaign, implying substantial streams of lignocellulosic residues (pruning wastes and almond shells).^27^

In this work, almond tree pruning residues and almond shells are used as sustainable lignocellulosic precursors to produce carbon-based cathodes for ZHSCs. These nonedible agro-residues, generated in large quantities during orchard management and nut processing, are valorized as low-cost carbon sources, aligning with circular-economy principles and reducing reliance on fossil-derived inputs and critical resources for electrochemical energy storage. Achieving hierarchical micro- and mesoporosity under relatively mild activation conditions without severe carbon loss remains a key challenge for biomass-derived carbons. In this work, we show that hydrothermal pretreatment prior to K_2_CO_3_ activation promotes pore development while limiting excessive burnoff. As a result, this strategy provides a practical route to balancing porosity and carbon under comparatively mild activation conditions.

## Experimental Section

### Carbon Precursors

The raw almond tree pruning residues (AT) and almond shells (AS) used here were obtained from an almond orchard in Huesca province (Spain). The content of extractives was determined by Soxhlet extraction using ethanol for 24 h under reflux.^28^ The extractives-free residues were then analyzed by thermogravimetric analysis (TGA), using a MK2-M5 thermobalance (CI Precision, UK), to estimate hemicellulose, cellulose, and lignin fractions. Approximately 10 mg of sample was heated to 950 °C at 5 °C min^–1^ under 250 cm^3^ min^–1^ (STP) of synthetic air. Proximate and ultimate analyses were conducted in triplicate to determine the volatile matter, fixed carbon, and ash contents, as well as the elemental composition (C, H, N, S). For proximate analysis, 1 g of biomass was dried at 105 ± 5 °C to constant mass, heated in a covered crucible at 925 ± 10 °C for 7 min to determine volatile matter, and then heated uncovered at 730  ±  10 °C for 2 h to determine ash content. Ultimate analysis was carried out using a CHN628 elemental analyzer (LECO, USA) according to ASTM D5373–16. The inorganic composition of the biomass ash was determined via X-ray fluorescence (XRF) using an ARL ADVANT’X-2331 (Thermo Fisher Scientific, Switzerland) spectrometer under helium atmosphere. Data was processed with UNIQUANT software for semiquantitative analysis.

Fourier-transform infrared (FTIR) spectra of biomass precursors and hydrochars were recorded using a Cary 630 FTIR (Agilent Technologies, USA) spectrometer over the wavenumber range of 4000–400 cm^–1^. Spectral acquisition and processing were performed using MicroLab FTIR software. Morphological features of both precursors and hydrochars were analyzed by scanning electron microscopy using an Apreo ChemiSEM System (Thermo Fisher Scientific, USA). Prior to SEM imaging, the biomass samples were sputter-coated with a thin Pd layer.

### Synthesis of Biomass-Derived Carbons

Both biomass precursors were ground and sieved to a particle size of 1.4–4.0 mm. The hydrothermal pretreatment was carried out by dispersing 7 g of biomass in 50 g of distilled water and heating the suspension at 185 °C for 12 h in a 100 mL Teflon-lined stainless-steel autoclave under autogenous pressure. After cooling at room temperature, the solid product (hydrochar) was recovered by vacuum filtration. For chemical activation, the dried hydrochar was physically mixed (dry blending) with the activating agent (KOH or K_2_CO_3_) at hydrochar-to-activating agent mass ratios ranging from 1:1 to 1:4. The mixtures were then carbonized/activated in a tubular furnace (Carbolite TF1 16/60/300, Germany) under an argon atmosphere at 800 °C for 2 h, using a heating rate of 5 °C min^–1^. The resulting carbons were washed sequentially with distilled water, 2 mol dm^–3^ HCl to remove residual inorganic species, and then copious distilled water until neutral pH was reached. Finally, the porous BCs were dried and sieved to collect particles smaller than 90 μm. For comparison, control samples were prepared by direct carbonization of hydrochar without activation and by direct chemical activation of the raw biomass without hydrothermal pretreatment.

The sample nomenclature used in this work is summarized in Table 1. Each code indicates: (1) the biomass precursor, AT or AS; (2) the preparation route, where *H* denotes the hydrochar-based route and *D* direct activation of the raw biomass; and (3) the activation treatment, where *C* refers to K_2_CO_3_ activation, *H* to KOH activation, and *P* to pyrolysis without chemical activation. The final digits indicate the precursor-to-activating-agent mass ratio. For example, AT-HC14 denotes an AT-derived carbon prepared through the hydrochar route (*H*) and activated with K_2_CO_3_ (*C*) at a hydrochar-to-activating agent ratio of 1:4.

**Table 1:** Sample Nomenclature and Synthesis Conditions

sample ID	preparation route	Activation treatment	precursor:activating agent mass ratio
AT-HC12	hydrochar route	K_2_CO_3_ activation	1:2
AT-HC14	hydrochar route	K_2_CO_3_ activation	1:4
AT-HH11	hydrochar route	KOH	1:1
AS-HC12	hydrochar route	K_2_CO_3_	1:2
AS-HC14	hydrochar route	K_2_CO_3_	1:4
AS-HH11	hydrochar route	KOH	1:1
AT-DC	direct activation	K_2_CO_3_	1:4
AS-DC	direct activation	K_2_CO_3_	1:4
AT-HP	hydrochar route	pyrolysis only	–
AS-HP	hydrochar route	pyrolysis only	–

### Physicochemical Characterization of Carbons

The morphology of the carbons was examined by scanning electron microscopy (SEM; Inspect F50, FEI, The Netherlands), while elemental mapping (EDS) was performed with the above-mentioned Apreo ChemiSEM System. High-resolution microstructural features were analyzed by transmission electron microscopy (HR-TEM; Tecnai F30, FEI) operated at 300 kV using a SuperTwin objective lens (0.19 nm point resolution). Interlayer spacings were obtained from HR-TEM images using DigitalMicrograph by averaging multiple measurements from representative regions. X-ray diffraction (XRD) patterns were collected with an Empyrean diffractometer (Malvern Panalytical, UK) using Cu Kα radiation (λ = 0.154 nm). Raman spectra were acquired using an Alpha300 microscope (WITec, Germany) with a 532 nm excitation laser.

Surface chemical composition and bonding states were characterized by X-ray photoelectron spectroscopy (XPS; AXIS Supra, Kratos Analytical, UK) using Al Kα radiation (1486.6 eV). High-resolution C 1s, O 1s, and N 1s spectra were fitted in CasaXPS software with mixed Gaussian–Lorentzian peak shapes after Shirley background subtraction.

Textural properties were determined from N_2_ adsorption isotherms at −196 °C and CO_2_ adsorption isotherms at 0 °C, both measured with an Autosorb iQ^3^ gas sorption analyzer (Anton Paar QuantaTec, USA). Prior to analysis, samples were degassed under vacuum at 150 °C for 8 h. Pore size distributions were estimated from the N_2_ isotherms using a QSDFT adsorption model for carbon with slit- and cylindrical-shaped pores, and from the CO_2_ isotherms using a Monte Carlo model, as implemented in QuadraWin 6.0. The specific surface area was additionally calculated by the Brunauer–Emmett–Teller (BET) method for comparative purposes.

### Electrochemical Performance of ZHSCs

Aqueous slurries were prepared by dispersing the as-produced BCs with sodium carboxymethyl cellulose (Na-CMC) as binder (5 wt %) and, when specified, acetylene black as conductive additive (10 wt %). The slurries were coated onto stainless-steel (AISI 316) foil to obtain an active-material loading of ca. 1.5 mg cm^–2^; the resulting films were then punched into 12 mm diameter disk electrodes. The resulting working electrodes (cathodes) were vacuum-dried at 120 °C for 12 h. The electrochemical performance was evaluated in two-electrode Swagelok-type cells using Zn metal as the anode, either 2 mol dm^–3^ ZnSO_4_ or 1 mol dm^–3^ Zn­(CF_3_SO_3_)_2_ (abbreviated as ZTFS) as the electrolyte, and a glass fiber membrane (Prat Dumas, France) as the separator. The thickness of carbon electrodes was measured using a TOB-DTT-25 digital micrometer (Tob Machine, China).

Electrochemical measurements were conducted using a MultiEmStat4 HR potentiostat/galvanostat (PalmSens, The Netherlands) at room temperature. Galvanostatic charge–discharge (GCD) tests were performed at current densities ranging from 0.1 to 20 A g^–1^ (normalized to the mass of active material in the cathode) within a cell voltage window of 0.1–1.7 V. Cyclic voltammetry (CV) was conducted at scan rates of 10, 20, 30, 40, and 50 mV s^–1^ to study the charge-storage mechanism. The dependence of the peak current (*i*) and scan rate (*v*) was analyzed using the power-law dependence

1 $$i=a\nu^{b}$$

where *a* is a constant and *b* is the slope of the log­(*i*) versus log­(*ν*) plot. Values of *b* approaching 1 and 0.5 are commonly associated with surface-controlled (capacitive) and diffusion-controlled processes, respectively. The capacitive and diffusion-controlled contributions were further quantified using Dunn’s method,^29^

2 $$i\left(\nu \right)=k_{1}\nu +k_{2}\nu^{1/2}$$

where the first and second terms represent the capacitive and diffusion-controlled current contributions, respectively; *k*_1_ and *k*_2_ were determined by linear fitting at each potential.

Electrochemical impedance spectroscopy (EIS) measurements were performed over the frequency range from 100 kHz to 0.01 Hz using a 10 mV AC perturbation. The impedance spectra were fitted to an equivalent-circuit model using the Python package *impedance.py*.^30^ Galvanostatic intermittent titration technique (GITT) measurements were conducted at 0.03 A g^–1^ using 5 min current pulses followed by 1 h relaxation periods at open-circuit voltage (OCV). Self-discharge was evaluated under open-circuit conditions by charging the cells to 1.1, 1.3, 1.5, and 1.7 V and monitoring the OCV decay for 48 h; voltage retention was calculated as the ratio of remaining voltage to initial voltage, *V*(*t*)/*V*_0_. Extended open-circuit measurements up to 72 h were additionally performed at 1.5 V, and the resulting voltage-decay curve was analyzed using a mixed-mechanism fitting approach.

The specific energy density (*E*, Wh kg^–1^) was calculated from the galvanostatic discharge profiles as

3 $$E=\frac{I}{3.6m}\int Vdt$$

where *I* (A) is the applied current, *m* (g) the mass of active material in the cathode, and the integral term corresponds to the area under the experimental discharge curve The specific power density (*P*, W kg^–1^) was obtained from

4 P=(E·3600)/Δt

where Δ*t* (s) is the discharging time. All electrochemical performance metrics, including specific capacitance, energy density, and power density, were calculated based on the mass of active material in the cathode.

## Results and Discussion

### Characterization of Raw Biomasses and Produced Hydrochars

Understanding the compositional differences between AT and AS biomasses is essential, as variations in extractives and biopolymer contents can critically influence their thermochemical conversion.^31^ Soxhlet extraction yielded 33.1 wt % extractives from AT, compared to only 9.1 wt % from AS. The higher extractives content in AT—likely including oils, resins and waxes—was expected given the role of pruning residues in sap transport, wound sealing, and stress response, whereas shells mainly consist of inert protective tissues.^32^

The TGA curves obtained for the extractive-free biomasses were analyzed by deconvoluting the differential thermogravimetric (DTG) profiles using a MATLAB routine, assuming Gaussian peak shapes to estimate the contributions from hemicellulose (two peaks), cellulose (two peaks), and lignin (three peaks). Figure S1 (Supporting Information) shows the resulting DTG deconvolutions for both samples. This approach, partly based on that reported by Shapiro et al.,^33^ provides a rapid semiquantitative assessment of the main lignocellulosic components. As summarized in Table 2, AS showed slightly higher cellulose and lignin fractions than AT, suggesting a modestly more thermally resilient matrix.

**Table 2:** Composition of the Raw Biomass Samples Used in This Study

	lignocellulosic constituents (wt % in dry and extractive-free basis)		
sample	hemicellulose	cellulose	lignin
AT	50.3	18.7	31.0
AS	47.8	20.2	32.0

	proximate analysis (wt % in dry basis)		
sample	Volatile matter	fixed carbon	ash
AT	79.7 ± 0.8	18.3 ± 0.7	2.0 ± 0.1
AS	81.7 ± 0.4	18.1 ± 0.4	0.14 ± 0.02

	ultimate analysis (wt % in daf basis)	
element	AT	AS
C	43.9 ± 0.2	46.3 ± 0.0
H	6.63 ± 0.03	6.47 ± 0.01
N	0.98 ± 0.08	0.35 ± 0.06
S	0.04 ± 0.00	0.02 ± 0.00
O (by difference)	48.4	46.9

Proximate analysis (Table 2) showed comparable volatile matter and fixed carbon in both biomasses; however, AT exhibited a higher ash content than AS (2.0 vs 0.14 wt %). Surface EDX spectra indicated carbon and oxygen as the predominant elements in both samples, with calcium more prominent in AT (2.4 wt %) and potassium enriched in AS (1.2 wt %), as shown in Figure S2. XRF analysis of the corresponding ashes (Figure S3) corroborated these trends, revealing a Ca-rich ash in AT (52.4 wt %), and a K-rich ash in AS (54.9 wt %). Ultimate analysis indicated higher oxygen and nitrogen contents in AT, while sulfur was almost negligible in both samples.

Building on the characterization of the raw biomass (AT and AS), FTIR spectra of the corresponding hydrochars (AT-HTC and AS-HTC) were analyzed to assess the modifications induced by hydrothermal pretreatment (Figure S4). For both precursors, the intensity of O–H stretching band (3200–3600 cm^–1^) and aliphatic C–H stretching bands (2800–3000 cm^–1^) decreased after HTC, consistent with dehydration and partial decomposition of cellulose- and hemicellulose-derived structures. Changes in the C–O and C–O–C stretching region (1000–1300 cm^–1^) further support the transformation of oxygenated aliphatic functionalities during HTC. The band around 1600 cm^–1^, commonly associated with aromatic C=C vibrations and/or conjugated structures, shows a slightly more pronounced development in AS-HTC than in AT-HTC, pointing to precursor-dependent differences in structural reorganization. In addition, changes in the 1700–1300 cm^–1^ indicate modifications in O-containing functional groups after HTC. Overall, these results suggest that hydrothermal pretreatment reduces hydroxyl- and aliphatic-rich functionalities and promotes the conversion of the raw biomass into more condensed carbonaceous intermediates.

SEM images of the raw biomass and the corresponding hydrochars (Figure S5) also reveal clear morphological changes after HTC. AT exhibits a loosely packed fibrous morphology, whereas AS appear denser and more compact, with more consolidated regions. After HTC, both materials evolve into irregular, fragmented particles with rougher surfaces. AT-HTC still retains partially collapsed elongated features, while AS-HTC displays a greater abundance of spherical features together with compact agglomerated regions. These observations are consistent with the FTIR results and support that HTC induces substantial chemical and morphological reorganization of the biomass precursors, which likely influences their subsequent response to chemical activation.

### Mass Yields during Hard Carbon Production

Table 3 summarizes the mass yields after hydrothermal pretreatment and subsequent activation/pyrolysis, together with the overall yield and the burnoff degree associated with each process condition. The latter was calculated as the mass loss relative to the corresponding pyrolyzed sample obtained without chemical activation. Overall, AS-derived carbons show slightly higher solid yields than their AT counterparts, in line with the compositional differences discussed above. Direct activation with K_2_CO_3_ (AT-DC and AS-DC) led to low burnoff degrees (4.68% and 4.83%, respectively), indicating a limited extent of activation under these conditions. Introducing the hydrothermal pretreatment increased the burnoff degree for AT, reaching 6.96% for AT-HC14, whereas its effect was much less pronounced for AS (4.94%). This suggests that the HTC step more effectively modifies the AT precursor, through partial hydrolysis and the generation of incipient narrow microporosity,^34^ thereby facilitating subsequent chemical activation. In contrast, KOH activation was substantially more aggressive, resulting in markedly higher burnoff degrees of 31.8% and 28.0% for AT-HH11 and AS-HH11, respectively.

**Table 3:** Mass Yields (wt % in Dry Basis) and Burn-off Degrees (%) for All Carbons

sample ID	HTC yield	activation/pyrolysis yield	overall yield	burnoff degree
AT-HC12	65.1	29.8	19.4	5.00
AT-HC14	65.1	29.2	19.0	6.96
AT-HH11	65.1	21.4	13.9	31.8
AS-HC12	62.5	35.1	21.9	3.40
AS-HC14	62.5	34.5	21.6	4.94
AS-HH11	62.5	26.1	16.3	28.0
AT-DC	–	21.6	21.6	4.68
AS-DC	–	25.6	25.6	4.83
AT-HP	65.1	31.3	20.4	–
AS-HP	62.5	36.3	22.7	–

### Physicochemical Properties of Hard Carbons

SEM micrographs of the raw biomasses, the directly activated carbons (AT-DC, AS-DC), and the hydrothermally pretreated, nonactivated carbons (AT-HP, AS-HP) are shown in Figure 1. The carbons produced by direct activation (Figure 1b,e) largely preserve the structural features of the parent biomasses (Figure 1a,d), with only mild surface etching and a predominantly rough texture. This limited morphological evolution, consistent with the low burnoff degrees discussed above, is compatible with a relatively mild activation effect under these conditions, where CO_2_/CO generated from K_2_CO_3_ at high temperature can locally gasify the carbon surface and promote subtle etching and pore widening.^35^ As observed, AT-derived carbon retains elongated transport channels typical of pruning residues, whereas AS-derived carbon maintains the intrinsic macroporous domains of the shell, with localized pore enlargement in some regions.

**Figure 1:**
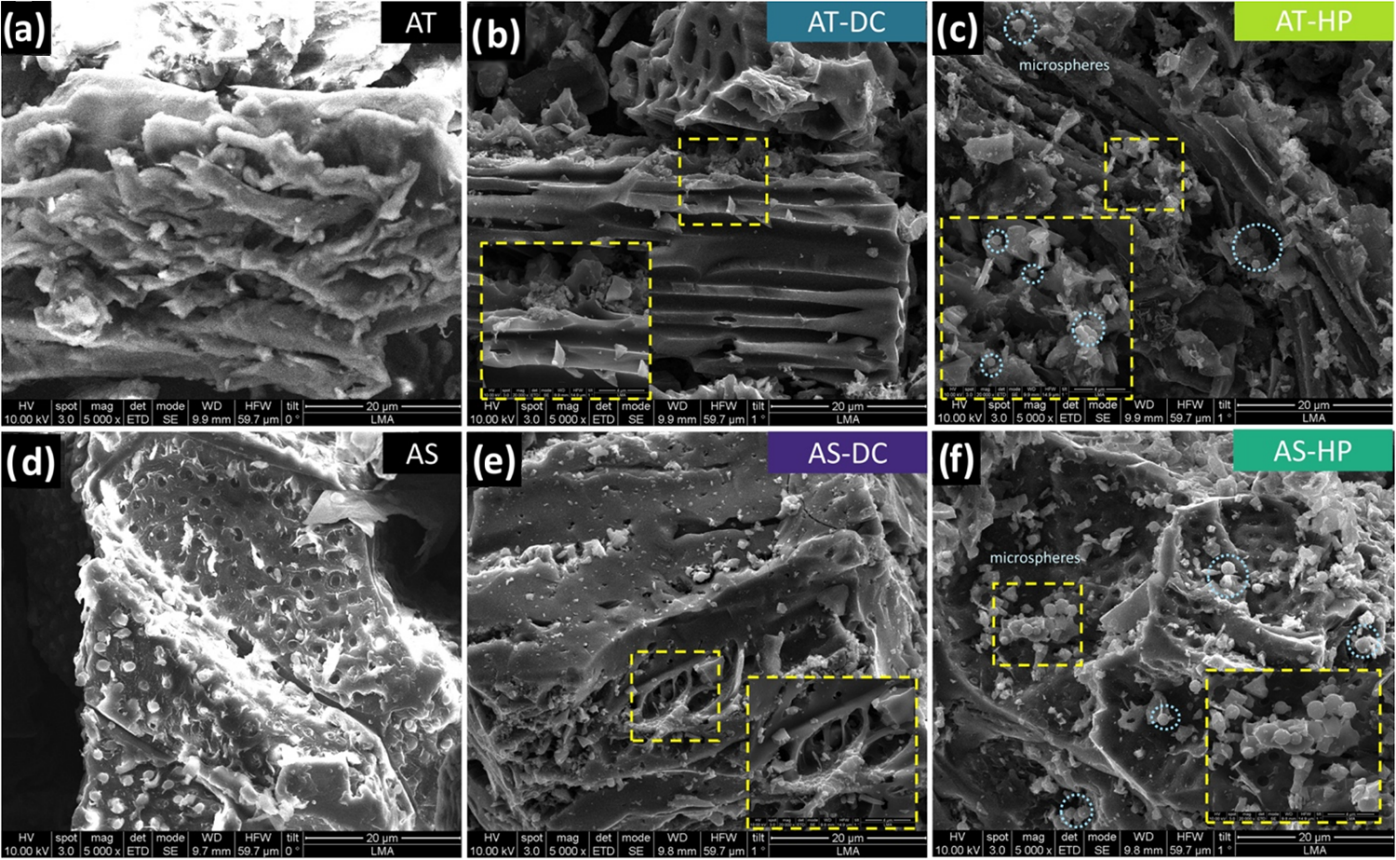
SEM micrographs of AT (a), AT-DC (b), AT-HP (c), AS (d), AS-DC (e), and AS-HP (f).

In contrast, the hard carbons synthesized through HTC followed by pyrolysis at 800 °C (Figure 1c,f) exhibit a more evident surface transformation, including the appearance of carbonaceous microspheres, while still preserving the hierarchical lignocellulosic framework of the precursors. These microspheres are attributed to HTC-driven hydrolysis/dehydration and subsequent polymerization/condensation reactions, yielding secondary carbon-rich features that may enhance electrolyte accessibility and provide additional active surface for ion storage.^36^

The morphological evolution induced by the cascaded HTC and chemical activation route is shown in Figures 2 and S6–S11. For almond tree pruning residues, AT-HC12 still displays recognizable biomass-derived motifs together with a limited number of microspheres and a relatively smooth, weakly porous surface. Increasing the activating agent loading (AT-HC14) led to a more pronounced surface transformation, with smoothing and partial loss of the honeycomb-like features. By contrast, KOH activation produced a heavily etched texture and the development of interconnected voids (Figure 2c), consistent with its markedly higher burnoff degree. The almond shells-derived carbons follow the same overall trend. K_2_CO_3_-activated carbons preserved the intrinsic macroporosity of the shell, while showing progressively stronger smoothing/etching and fragmentation as the activating-agent loading increased (Figures 2d,e). Conversely, KOH activation generated strongly etched surfaces with abundant microporosity (Figure 2f); in some regions, signs of partial collapse of porous domains are observed, suggesting an overactivation regime and local structural degradation.

**Figure 2:**
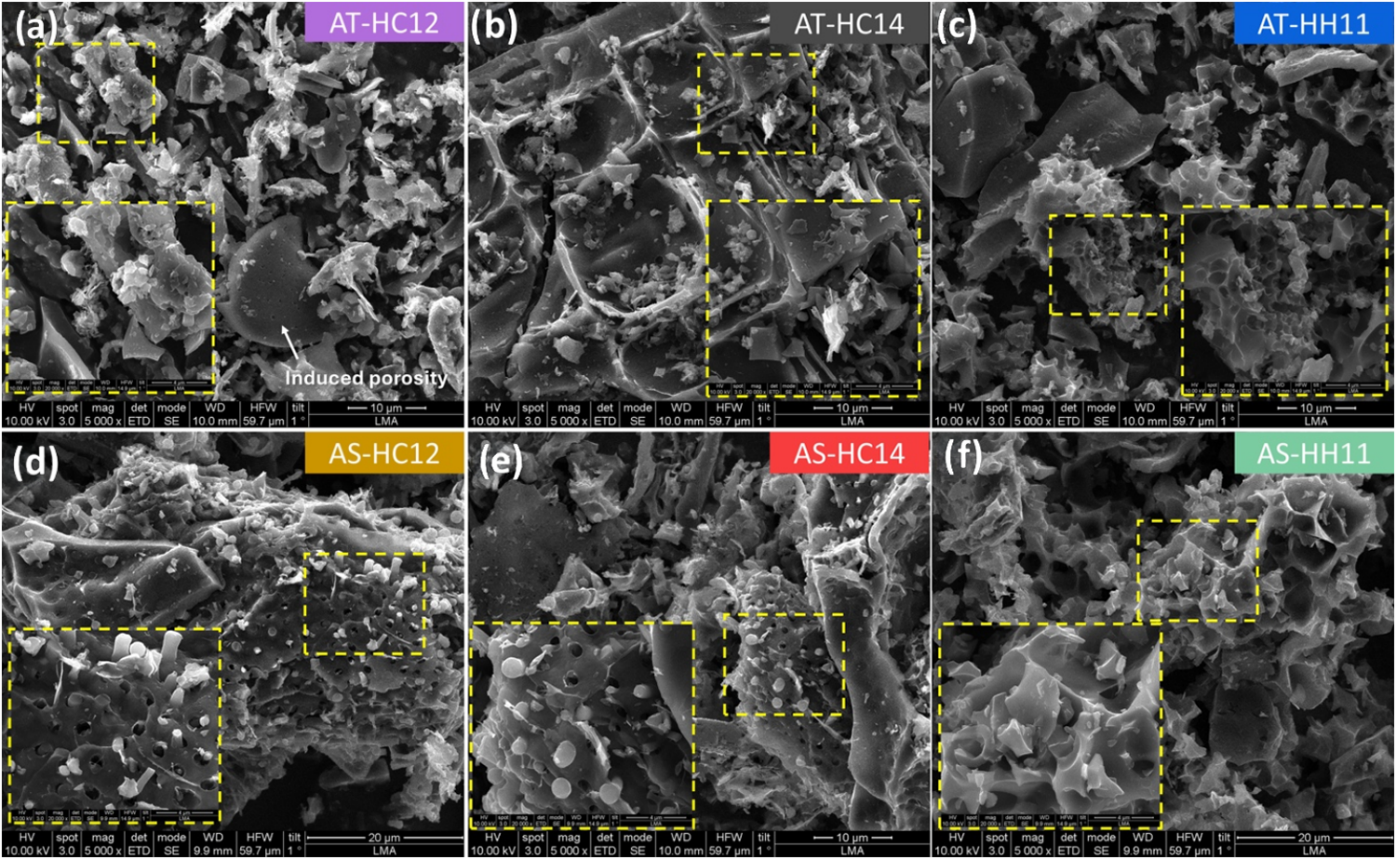
SEM micrographs of AT-HC12 (a), AT-HC14 (b), AT-HH11 (c), AS-HC12 (d), AS-HC14 (e), and AS-HH11 (f).

Figures 3 (and Figures S12–S18) shows HR-TEM images of the resulting carbons. All samples display typical hard-carbon features, with turbostratic domains composed of curved, randomly oriented graphene layers and no extended graphitic stacking. Across the series, lattice fringes consistent with expanded interlayer distances in the range 0.36–0.48 nm are observed (Figures 3 and S12), with the largest values typically found for AT-HC12 and AT-HC14 (Figure 3a,b). These spacings are larger than those commonly reported for biomass-derived carbons obtained at comparable highest treatment temperatures (i.e., 0.37–0.42 nm).^37^ The AS-derived carbons show a similar response under K_2_CO_3_ activation, exhibiting predominantly disordered domains with expanded interlayer distances (Figure 3d,e).

**Figure 3:**
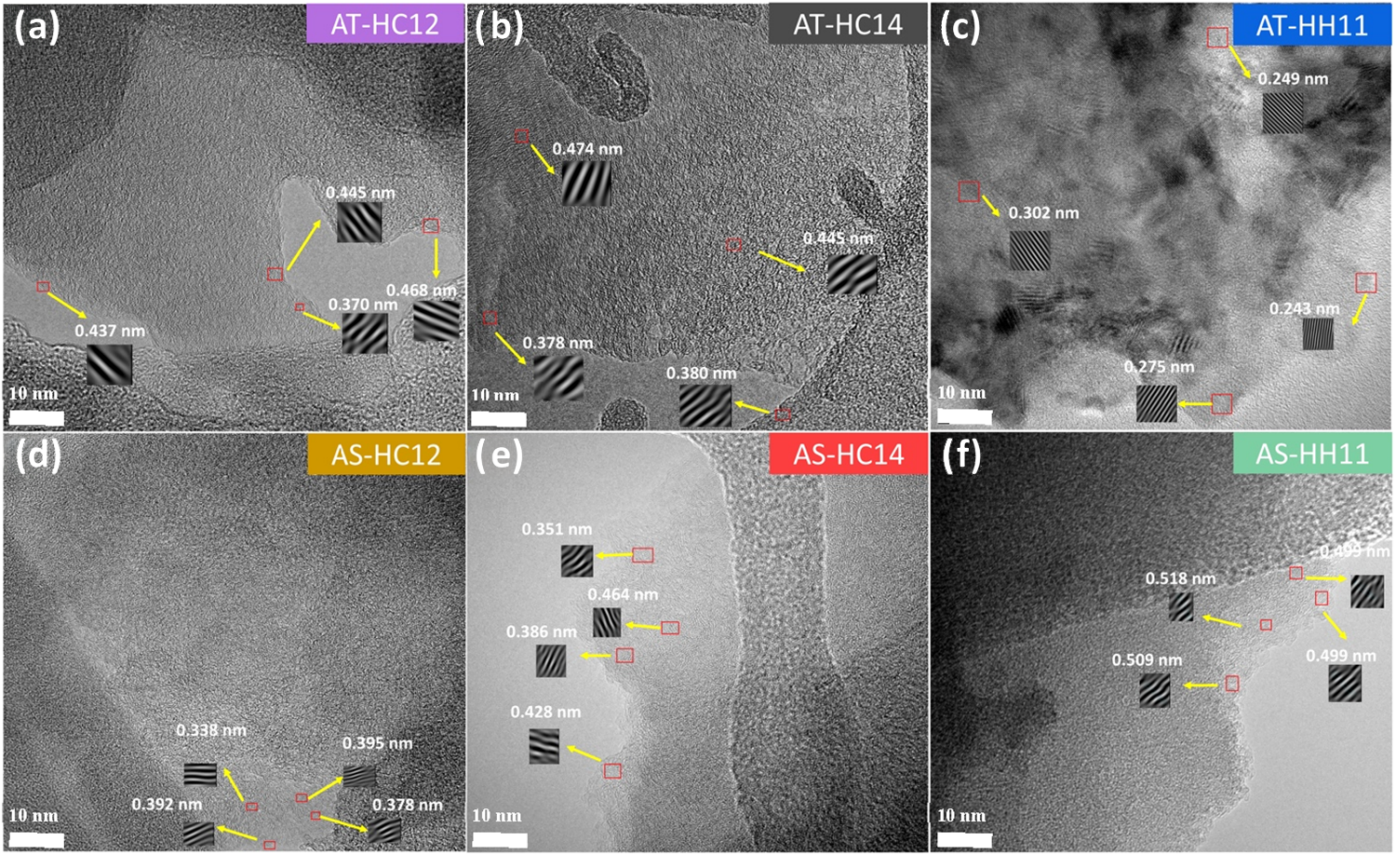
HR-TEM images of AT-HC12 (a), AT-HC14 (b), AT-HH11 (c), AS-HC12 (d), AS-HC14 (e), and AS-HH11 (f).

Interestingly, activation with KOH led to different features depending on the biomass source. AT-HH11 exhibits a heterogeneous structure, in which low-ordering turbostratic regions with expanded interlayer spacings coexist with locally more ordered graphitic-like motifs characterized by shorter lattice-fringe spacings (0.24–0.30 nm) and a higher number of aligned carbon layers (Figures 3c and S15). This behavior is consistent with the higher reactivity of KOH, where redox-driven etching and the formation of reactive K-containing intermediates (e.g., molten K_2_O and metallic K) may promote local rearrangement toward more ordered carbon in restricted regions, even as extensive gasification proceeds.^38,39^ In contrast, KOH activation in AS-HH11 (Figure 3f) does not show clear evidence of such local ordering and instead yields even more expanded turbostratic spacings (ca. 0.50 nm) together with pronounced macroporosity, consistent with the severe etching observed at lower magnification (Figure S18). A plausible contributing factor can be the denser, shell-derived architecture, which may limit the penetration/intercalation of reactive K-containing species into the carbon framework, thereby suppressing the development of graphitic-like motifs and favoring extensive etching and interlayer separation.

From the XRD patterns collected for the synthesized carbons, the nonactivated samples (AT-HP and AS-HP) display the broad (002) and (100) features typically observed for disordered/turbostratic carbons (Figure 4a). Upon chemical activation—particularly after hydrothermal pretreatment—the (002) and (100) profiles become markedly broader, consistent with increased structural disorder and/or reduced coherence lengths associated with pore development. In addition to these carbon features, several patterns show reflections attributable to KCl (JCPDS 00–041–1476). KCl signatures are observed for the directly activated samples (AT-DC and AS-DC) and for the hydrothermally pretreated, activated AS-derived carbons, whereas they are not detected for the HTC-assisted AT-derived samples. This contrast likely reflects differences in precursor architecture; a more compact shell-derived matrix may trap residual salts more effectively, leading to detectable crystalline KCl, whereas the more open AT-derived structure may facilitate salt removal below the XRD detection limit.

**Figure 4:**
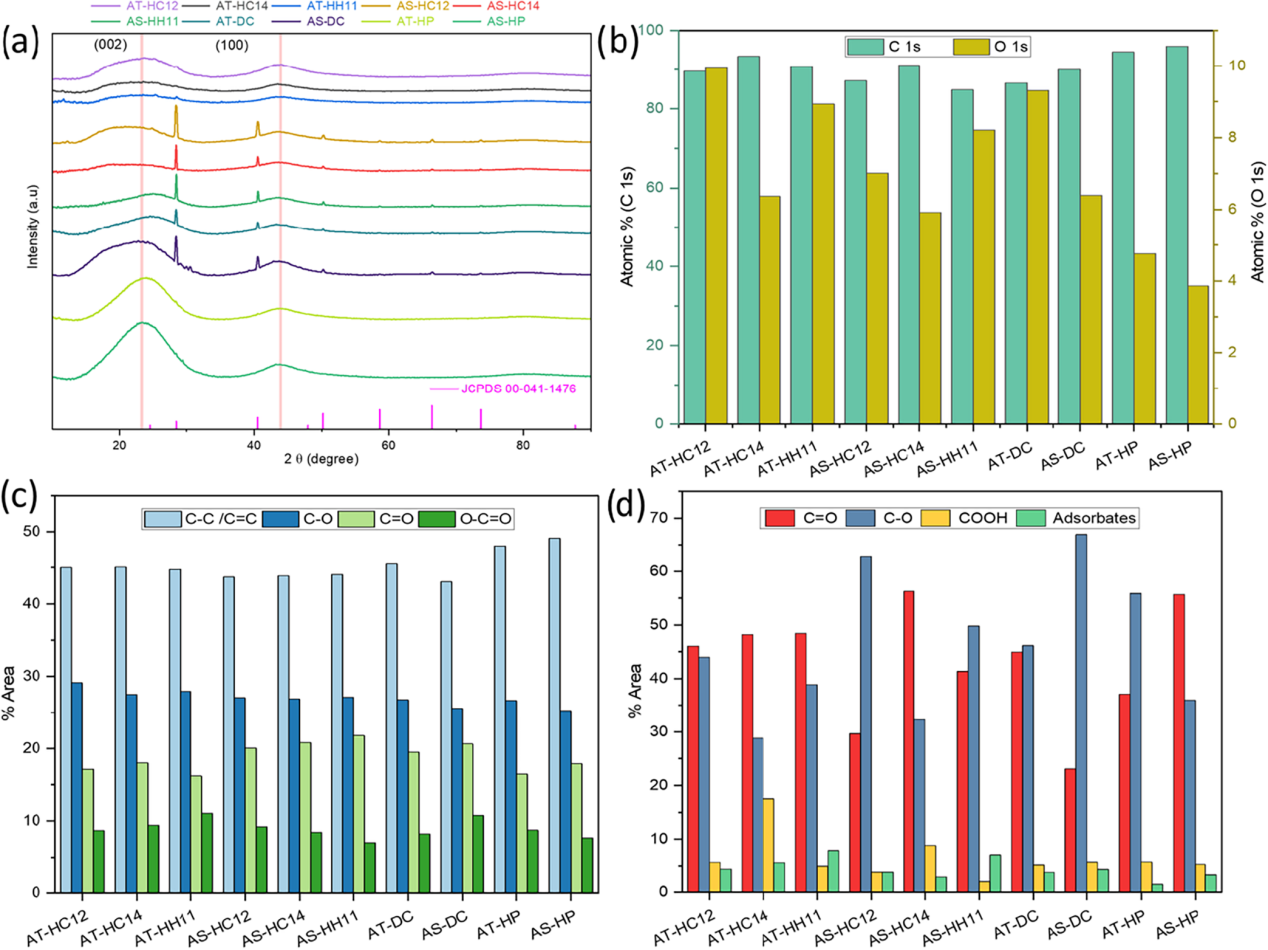
XRD patterns of all samples together with the KCl reference pattern (JCPDS 00–041–1476) (a), C and O atomic contents (at %) from the XPS C 1s and O 1s regions (b), and functional-group distribution derived from C 1s (c) and O 1s (d) peak deconvolution.

The residual KCl previously identified by XRD was further evaluated by bulk XRF analysis for AT-DC, AS-DC, AT-HC14, and AS-HC14 samples (Figure S19). The hydrothermally pretreated carbons show lower residual contents of K (0.06–0.53 wt %) and Cl (0.16–0.61 wt %) than the directly activated samples (0.54–1.30 wt % K and 0.34–0.62 wt % Cl), indicating that hydrothermal pretreatment is associated with a lower amount of residual inorganic species after washing. Surface-sensitive SEM-EDS mapping of AT-HC14 and AS-HC14 further supports that potassium- and chlorine-containing residues are present only at very low levels on the carbon surface, with AT-HC14 showing nearly negligible amounts (Table S1; Figures S20 and S21). Although no crystalline KCl is detected by XRD for AT-HC14, the presence of trace residual species remains consistent with the bulk compositional analysis.

Raman spectra (Figure S22) were processed using a custom Python-based analysis pipeline including baseline extraction and intensity normalization prior to quantitative analysis. To avoid overparametrization, the first-order region was fitted by nonlinear least-squares using only the D (≈1350 cm^–1^, Lorentzian), D′ (1610–1625 cm^–1^, Gaussian), and G (1590–1600 cm^–1^, Lorentzian) bands. From the fitted spectra, the following parameters were computed: band positions (ω_G_, ω_D_), band widths (fwhm_G_, fwhm_D_), and heigh peak-based *I*_D_/*I*_G_ ratios. As shown in Table S2, all samples exhibit very similar band positions together with broad features, indicating highly disordered/turbostratic carbons, for which Raman primarily reflects short-range ordering.^40^ In this regime, variations in *I*_D_/*I*_G_ and line widths are expected to be subtle and not uniquely convertible into a crystallite size, because the D region may include overlapping disorder-related contributions, and both peak widths and intensity ratios are strongly affected by the defect population. Only a modest increase in *I*_D_/*I*_G_ is observed for the chemically activated carbons (up to 1.09–1.13 vs 1.03–1.07 for the nonactivated ones), suggesting a small shift toward a higher contribution of defect/edge-related scattering rather than a change in structural ordering.

EDS analysis data (Table S3 and Figure S23) show that the nonactivated AT-HP and AS-HP samples are carbon-rich (93–94 at %) with minor oxygen, as expected for biomass thermally annealed at relatively high temperatures. After chemical activation, the oxygen content increases markedly (9–13 at %). Notably, samples prepared with the lower K_2_CO_3_ ratio (1:2) exhibit slightly higher oxygen than those activated at 1:4, suggesting that milder conditions may better preserve oxygen-containing functionalities, whereas harsher activation can promote further carbon gasification and partial deoxygenation.

Regarding surface chemistry, XPS data revealed marked changes as a function of precursor and activation conditions, as reflected in the high-resolution C 1s and O 1s core-level regions (Figures S24–S33). The C 1s envelopes were deconvoluted into four components centered at ≈284.3 eV (C–C/C=C), ≈285.9 eV (C–O), ≈287.3 eV (C=O), and ≈290.7 eV (O–C=O), whereas the O 1s profiles were fitted with contributions at ≈531.5 eV (C=O), ≈533.1 eV (C–O), ≈535.2 eV (COOH), and ≈537.8 eV (adsorbates). Survey quantification (Figure 4b and Table S4) shows that the nonactivated carbons display moderate oxygen contents (AT-HP: 4.76 at %; AS-HP: 3.86 at %), while chemical activation increases surface oxygen to 5.92–9.95 at % depending on precursor and route. For both precursors activated with K_2_CO_3_, the 1:2 ratio yields systematically higher O contents than 1:4, consistent with EDS and indicating higher retention of oxygenated species under milder conditions. Deconvolution trends further indicate that oxygen speciation shifts with activation severity (Figure 4c,d): the 1:2 samples are relatively enriched in C–O contributions, whereas the 1:4 samples show increased O–C=O/COOH-related fractions (i.e., a shift toward more acidic functionalities under harsher activation). In agreement with XRD, XRF, and EDS (Table S3), residual K and Cl signals were detected in AS-derived activated carbons and in directly activated samples (Table S4 and Figures S27–S31), pointing to incomplete removal of inorganic residues (likely KCl), while they remain below detection in AT-HC and AT-HH samples. Nitrogen (predominantly pyrrolic-type species) was only detected in AT-derived carbons, consistent with the higher N content in the raw biomass (Table 2).

Bulk elemental analysis by CHN (Table S5) provides complementary compositional information to the surface-sensitive results. The nonactivated carbons show the highest carbon contents, whereas chemical activation leads to a relative increase in oxygen content, consistent with the surface trends identified by XPS. In addition, the AT-derived carbons retain slightly higher nitrogen contents than the corresponding AS-derived samples, again in agreement with the XPS analysis.

Figure 5 summarizes the textural properties of the BCs derived from N_2_ and CO_2_ adsorption (see also Table S6, and Figures S34 and S35 for isotherms and estimated pore size distributions, respectively). The nonactivated chars (AT-HP and AS-HP) showy very low N_2_-accessible surface areas and negligible mesoporosity, consistent with a pore network dominated by very narrow micro/ultramicropores. Upon direct activation with K_2_CO_3_, AT-DC reached a high BET (N_2_) surface area (1208 m^2^ g^–1^), whereas the corresponding value for AS-DC is much lower (509 m^2^ g^–1^), indicating a markedly less developed N_2_-accessible porosity in the shell-derived carbon. Introducing hydrothermal pretreatment prior to activation leads to a clear increase in surface area and pore volume for both precursors. In particular, AT-HC14 and AT-HH11 reach BET (N_2_) surface areas of 1461 and 1494 m^2^ g^–1^, respectively. These results indicate that HTC pretreatment is associated with a more developed porous structure after activation. The pore volumes derived from the pore size distribution analysis (Figure 5b) further show that HTC-assisted activation yields a hierarchical pore network dominated by ultra/micropores together with a measurable mesopore contribution, which is favorable for electrolyte accessibility and ion transport.^41,42^

**Figure 5:**
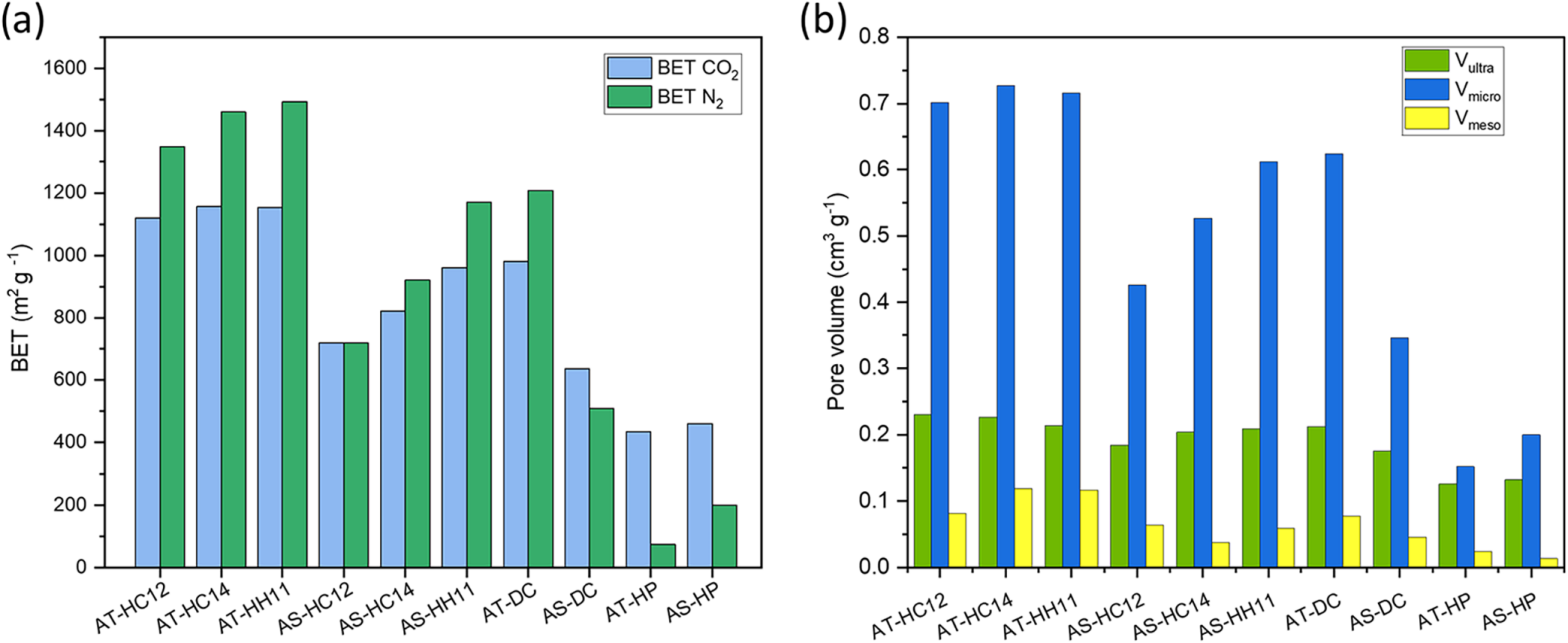
BET (N_2_) and BET (CO_2_) specific surface areas (a); ultramicropore (*V*_ultra_), micropore (*V*_micro_), and mesopore (*V*_meso_) volumes of the produced BCs.

Among the AT-derived samples, AT-HC14 combines high surface area with a relatively large mesopore volume (0.120 cm^3^ g^–1^), clearly higher than that of AT-DC (0.077 cm^3^ g^–1^) and comparable to that of AT-HH11 (0.117 cm^3^ g^–1^). In contrast, the AS-derived carbons show lower overall porosity development, particularly in the directly activated case. Overall, the combined HTC + K_2_CO_3_ activation route appears to provide the most balanced development of hierarchical porosity.

### Electrochemical Performance

All the synthesized hard carbons were first evaluated as cathodes in a two-electrode ZHSC configuration using ZTFS as electrolyte. The measured rate capability (0.1–20 A g^–1^) is summarized in Figure 6a,b. Regardless of the synthesis route, all activated AT-derived carbons outperform the AS counterparts, in line with their stronger porosity development (higher accessible surface and mesopore volume). The nonactivated carbons (AT-HP and AS-HP) delivered very low capacities, reflecting their limited accessible porosity and sluggish charge-storage kinetics. Direct chemical activation improved the response, particularly for AT-DC (88 mAh g^–1^ at 0.1 A g^–1^, Figure 6a); however, the capacity rapidly decayed as the current density increased, evidencing pronounced diffusion limitations consistent with its comparatively modest mesoporosity.

**Figure 6:**
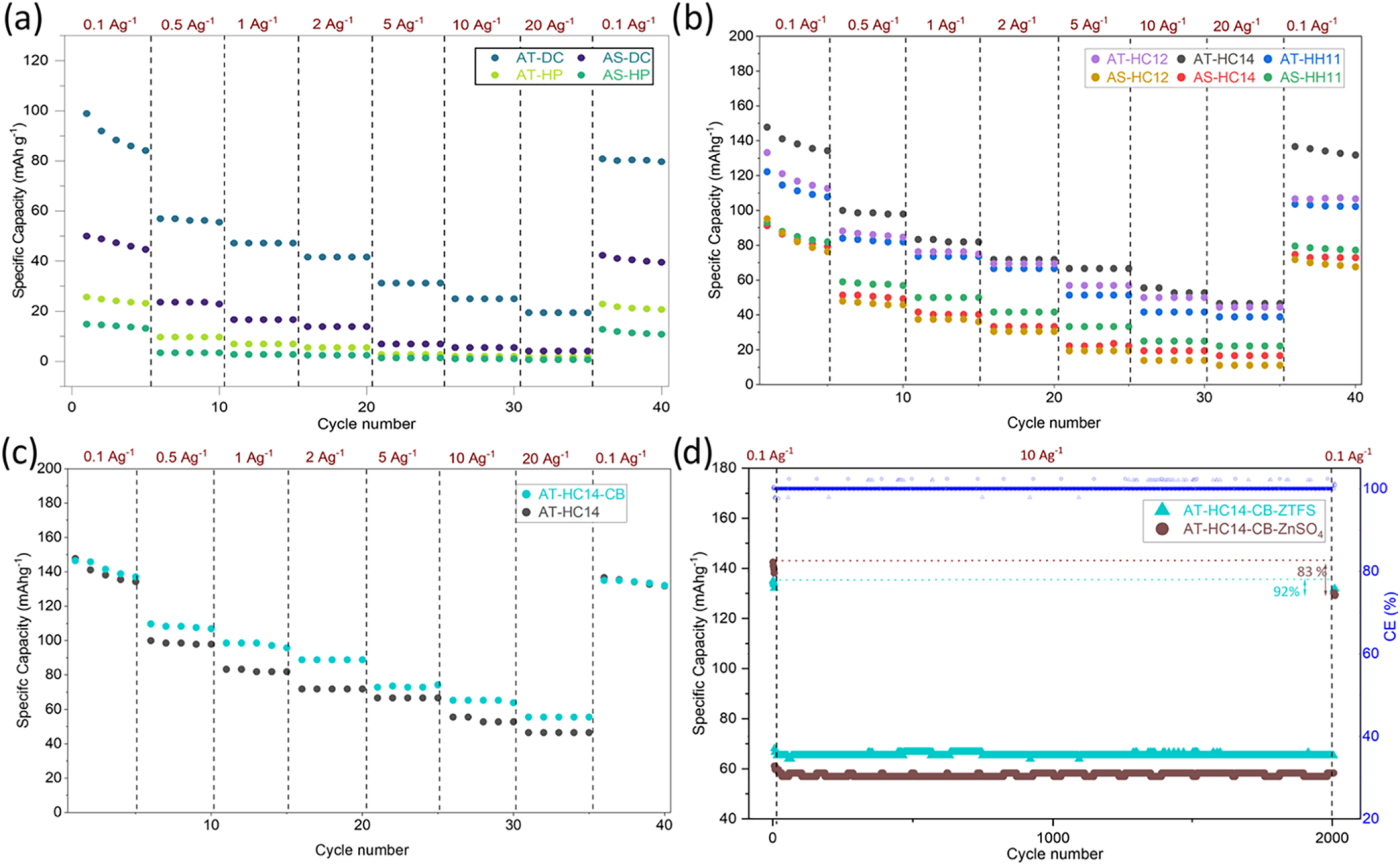
GCD cycling of BC-based cathodes at various current densities from 0.1 to 20 A g^–1^ (a–c); long-term cycling of AT-HC14-CB in ZTFS and ZnSO_4_ aqueous electrolytes (d).

Introducing hydrothermal pretreatment prior to activation produced a clear performance boost (Figure 6b). Among all samples, AT-HC14 delivered the highest capacity at low current (142 mAh g^–1^ at 0.1 A g^–1^) while preserving markedly higher capacities at elevated rates (66 and 46 mAh g^–1^ at 5 and 20 A g^–1^, respectively). This improved rate capability is primarily ascribed to its hierarchical pore network, where the larger mesopore contribution enhances accessibility and facilitates ion transport. In addition, the relatively oxygen-rich surface (C–O/C=O/COOH) may improve electrode wetting and contribute to pseudocapacitive charge storage.^43,44^

To further probe kinetic limitations, AT-HC14 was also tested with the addition of 10 wt % conductive carbon black (AT-HC14-CB, Figure 6c). While the low-rate capacity remains essentially unchanged, the conductive additive yields a consistent benefit as the current density increases. This behavior indicates that, once ion transport is partially alleviated by the hierarchical porosity, resistive limitations within the electrode become increasingly relevant at high current density, as also reflected by the pronounced *iR* drop in the GCD profiles (Figure S36) for the AT-HC14-CB-based cathode. The voltage loss reaches approximately 0.2 and 0.4 V at 10 and 20 A g^–1^, respectively, indicating non-negligible resistive losses under these high-rate conditions. Similar behavior has been reported for activated-carbon-based aqueous Zn-ion hybrid capacitors at comparable current density.^45^ Nevertheless, the comparative rate-performance trends among the different samples remain meaningful under identical testing conditions.

Cycling tests were also performed in a three-electrode cell using a second Zn disk as pseudoreference electrode in order to compare the cathode response with that obtained in the two-electrode cell. The specific capacities obtained in both configurations were very similar (Figure S37), indicating that, under the present conditions, the two-electrode setup provides a consistent basis for evaluating the cathode performance. These results therefore support the use of the two-electrode configuration for the comparative electrochemical assessment carried out in this work.

The electrochemical behavior of the best-performing electrode was further assessed at a higher active material loading of ca. 3.5 mg cm^–2^ (Figure S38). Although a moderate performance decrease is observed, consistent with increased transport limitations, the main capacity and rate-performance trends are maintained.

Long-term cycling stability was evaluated for AT-HC14-CB at 10 A g^–1^ over 2000 cycles, comparing ZTFS- and ZnSO_4_-based electrolytes (Figure 6d). In both cases, the device exhibits highly stable operation with high Coulombic efficiency. Importantly, the ZTFS electrolyte delivered higher and more stable capacity throughout prolonged cycling (≈65 mAh g^–1^) than ZnSO_4_ (≈58 mAh g^–1^). When the current density is returned to 0.1 A g^–1^, the capacity recovery is also superior in ZTFS (≈92% retention) compared with ZnSO_4_ (≈83%), evidencing better reversibility and fewer parasitic processes in the former electrolyte. The enhanced performance in ZTFS can be rationalized considering electrolyte-specific ion–solvent and ion-anion interactions in aqueous Zn^2+^ systems. Compared with sulfate, the triflate anion is less strongly coordinating and typically promotes weaker ion pairing and different solvation structures, which can mitigate concentration polarization and interfacial overpotentials during fast Zn^2+^ transport. Conversely, SO_4_^2–^ is a strongly coordinating divalent anion that can favor more tightly bound Zn^2+^ solvation/ion-association environments, increasing polarization under high-rate operation and accelerating performance decay.^46^

Post-mortem SEM analysis of Zn anodes recovered after cycling in both electrolytes highlighted differences in Zn deposition/stripping morphology that directly impact device stability (Figure 7). In ZnSO_4_, charging leads to the formation of large lamellar and dendritic deposits, visible as angular plate-like crystals associated with inhomogeneous nucleation and uncontrolled growth (Figure 7b). Such structures can increase the likelihood of local current hotspots and separator penetration and favor the generation of electrically isolated “dead Zn”, thereby lowering the plating/stripping efficiency. After discharge, the ZnSO_4_-cycled anode shows a rough and porous surface covered by granular aggregates (Figure 7d), consistent with incomplete stripping and surface passivation driven by corrosion byproducts. In contrast, ZTFS promotes compact, dense, and comparatively uniform Zn deposits (Figure 7e), in agreement with more reversible Zn plating/stripping during long-term operation. This behavior is commonly ascribed to the development of a thin, F-rich interphase (often reported to contain ZnF_2_), which suppresses parasitic reactions and mitigates dendrite growth.^47,48^ In addition, the lower water activity and weaker Zn^2+^-anion interactions in ZTFS reduce hydrogen evolution and Zn corrosion relative to ZnSO_4_, further stabilizing the metal–electrolyte interphase. Cross-sectional SEM images corroborate these trends: Zn deposited in ZTFS forms a thicker and more coherent layer (≈50 μm), whereas deposits formed in ZnSO_4_ are thinner (≈14 μm) and notably more porous/discontinuous (Figure 7c,f), evidencing denser growth and improved structural integrity under cycling.

**Figure 7:**
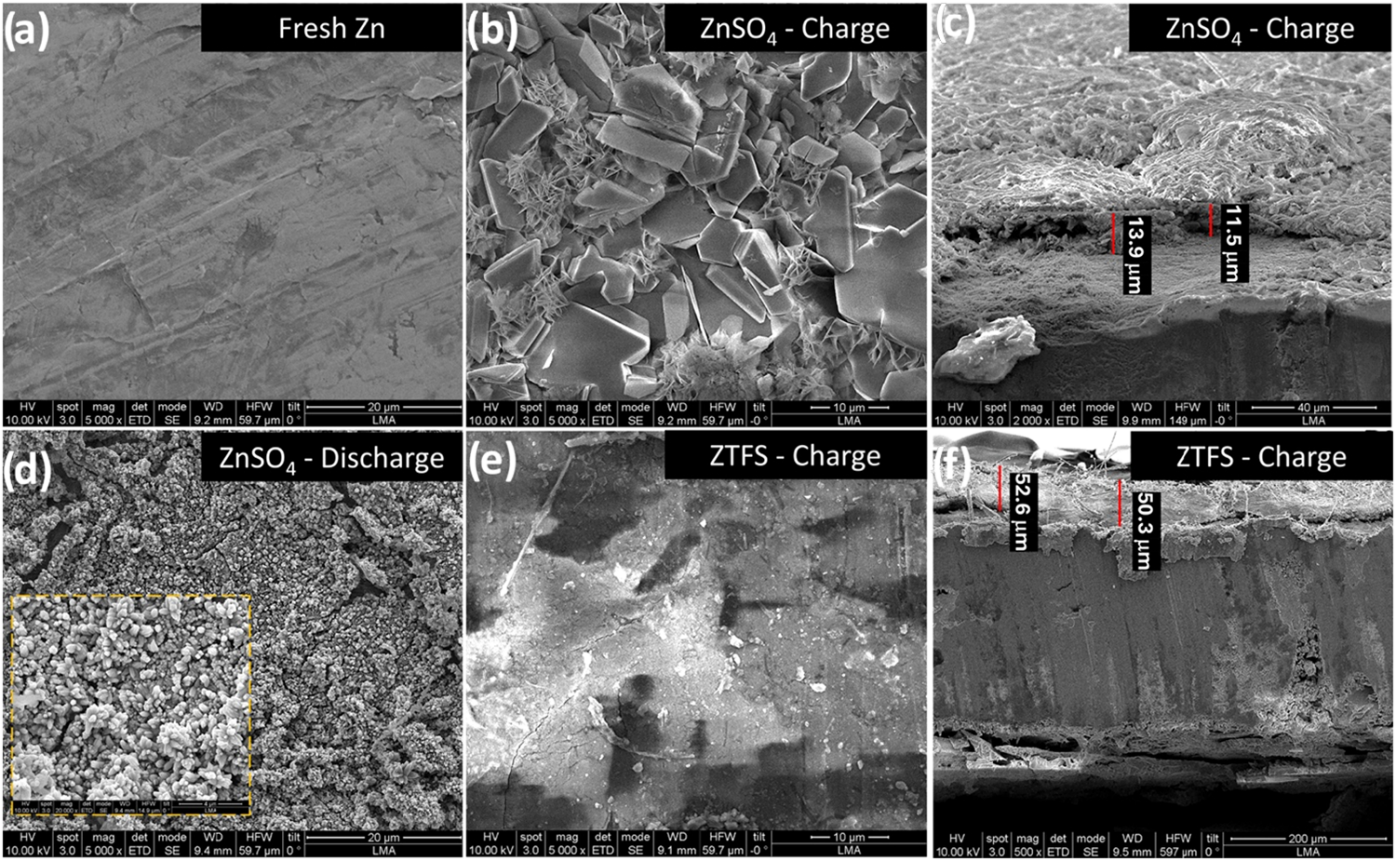
SEM images after 2000 cycles at 10 A g^–1^ of Zn anode: fresh (a), after charge and discharge cycles in ZnSO_4_-based electrolyte (b–d), and after charge in ZTFS-based electrolyte (e and f).

Post-mortem SEM of the AT-HC14-CB cathode after 2000 discharge cycles in ZTFS (Figure S39) supports the structural integrity of the carbon electrode. The heterogeneous particle/microsphere morphology is preserved, with no evidence of fracture, particle detachment, or collapse. To further assess possible thickness changes in the carbon electrode, measurements were performed using a digital micrometer. Comparable values were obtained for the pristine and cycled electrodes in ZTFS, both around 54 μm. Consistently, cross-sectional SEM images (Figure S39f) show a similar thickness of approximately 56 μm. Taken together, these results indicate that no significant thickness change occurs during cycling within experimental uncertainty, in agreement with the preserved morphology of the electrode.

Prolonged cycling stability of AT-HC14-CB was further evaluated at 10 A g^–1^ over 10,000 cycles in both electrolytes. Under these conditions, the device operating in ZTFS retained 91% of its initial capacity, whereas the system using ZnSO_4_ retained 82% (Figure S40). To investigate the origin of this electrolyte-dependent durability, post-mortem analyses were performed on both Zn anodes and carbon cathodes by XRD, SEM-EDS, and XPS. For the Zn anode cycled in ZnSO_4_, XRD revealed the appearance of additional reflections assigned to basic zinc sulfate hydroxide hydrate, Zn_4_(OH)_6_SO_4_·*x*H_2_O, indicating the formation of a crystalline hydroxy-sulfate surface phase (Figures S41a and S42, Table S7). This interpretation is supported by SEM, which shows well-defined crystalline deposits, and by EDS and XPS analyses, which detect substantial O and S contents together with Zn^2+^- and sulfate-related signatures (Figure S46 and Table S11 for SEM-EDS, and Figure S50 for XPS). Similar degradation products were also identified on the carbon cathode cycled in ZnSO_4_, where XRD indicates the presence of ZnSO_4_·6H_2_O and Zn_4_SO_4_(OH)_6_·xH_2_O, accompanied by significant Zn and S accumulation detected by SEM-EDS and XPS (Figures S41b, S44, S48, and S1; Tables S9 and S11). Altogether, these results indicate extensive sulfate-derived interfacial reactions in ZnSO_4_, affecting both electrodes and contributing to the larger performance decay.

In contrast, electrodes cycled in ZTFS show markedly lower deposition of crystalline byproducts. On the Zn anode, XRD mainly preserves the reflections of metallic Zn, with only weak and broad additional features, suggesting the presence of poorly crystalline or amorphous surface species (Figures S41a and S43, Table S8). SEM-EDS and XPS detect fluorine-containing species together with ZnF_2_-related and CF_3_-related contributions, consistent with the formation of a mixed fluorinated interphase (Figures S47 and S52). On the carbon cathode, only weak reflections assigned to zinc hydroxide carbonate were detected by XRD (Figures S41b and S45, Table S10), while SEM-EDS/XPS indicate limited surface deposition together with fluorinated and carbonate-containing species (Figures S49 and S53; Table S11). Cross-sectional SEM further shows that the electrode thickness remains essentially unchanged after cycling (54–58 μm), indicating preservation of the electrode architecture (Figure S54).

Overall, the post-mortem analyses show that ZnSO_4_ promotes the accumulation of abundant crystalline sulfate/hydroxy-sulfate byproducts on both electrodes, whereas ZTFS favors the formation of a less crystalline fluorine-containing interphase with more limited deposition. These differences in interfacial evolution are consistent with the higher capacity retention observed in ZTFS during long-term cycling. Although ZTFS provides improved cycling stability, ZnSO_4_ remains more attractive from a cost perspective and is still widely used in aqueous Zn-based systems.^49^ Therefore, the choice of electrolyte involves a trade-off between electrochemical durability and economic feasibility.

The self-discharge behavior of the ZHSC device (AT-HC14-CB cathode) was examined under open-circuit conditions (Figure S55). After charging to different initial voltages and resting for 48 h, all cases exhibited the expected gradual open-circuit voltage (OCV) decay, which becomes more pronounced at higher potentials. The device retained 26.3% and 24.8% of its initial voltage after 48 h when charged to 1.7 and 1.5 V, respectively, whereas lower voltages of 1.1 and 1.3 V showed slower decay. Such behavior is consistent with the known self-discharge tendency of porous-carbon-based aqueous Zn-ion hybrid capacitors, where noticeable voltage decay over tens of hours is commonly observed under open-circuit conditions.^50^

To further examine the long-time voltage-decay behavior, an extended self-discharge measurement up to 72 h was conducted at 1.5 V (Figure S56). The resulting curve was analyzed using a mixed-mechanism fitting approach following the method reported by Yang et al.^50^ The fitting suggests a multistage behavior composed of an initial exponential decay associated with ohmic leakage, an intermediate diffusion-related regime attributed to gradual ion redistribution within the hierarchical pore network, and a slower long-time relaxation consistent with faradaic and/or deep-pore processes. Overall, this analysis supports that self-discharge in the present hybrid supercapacitor arises from combined electrostatic and kinetic contributions rather than from a single dominant process.

Figure 8a displays the CV curves obtained for AT-HC14-CB at scan rates from 10 to 50 mV s^–1^. The CVs exhibit quasi-rectangular shapes with only weak redox humps, consistent with a predominantly capacitive response of the porous hard-carbon cathode, with minor pseudocapacitive contributions associated with surface oxygen-containing functionalities and the Zn^2+^/Zn reaction at the metal anode. Based on the power-law relationship in eq 1, b values of 0.87 and 0.85 were obtained for the anodic and cathodic peaks, respectively (Figure 8b), indicating a hybrid charge-storage regime with a strong surface-controlled component and a non-negligible diffusion contribution. This trend is corroborated by Dunn’s analysis and the resulting partition of capacitive and diffusion-controlled currents (Figure 8c), showing that the capacitive fraction increases from 64% at 10 mV s^–1^ to 80% at 50 mV s^–1^ (Figure 8d). Although Dunn’s method provides only an approximate kinetic partition and does not imply complete independence between capacitive and diffusion-controlled processes in porous electrodes, the increasing capacitive contribution at higher scan rates is consistent with a progressively greater dominance of surface-controlled charge-storage processes within the hierarchical pore network. The remaining diffusion-controlled fraction can be ascribed to mass-transport limitations, including ion transport through the bulk electrode, in agreement with the trends observed in the GCD profiles (Figure S36).

**Figure 8:**
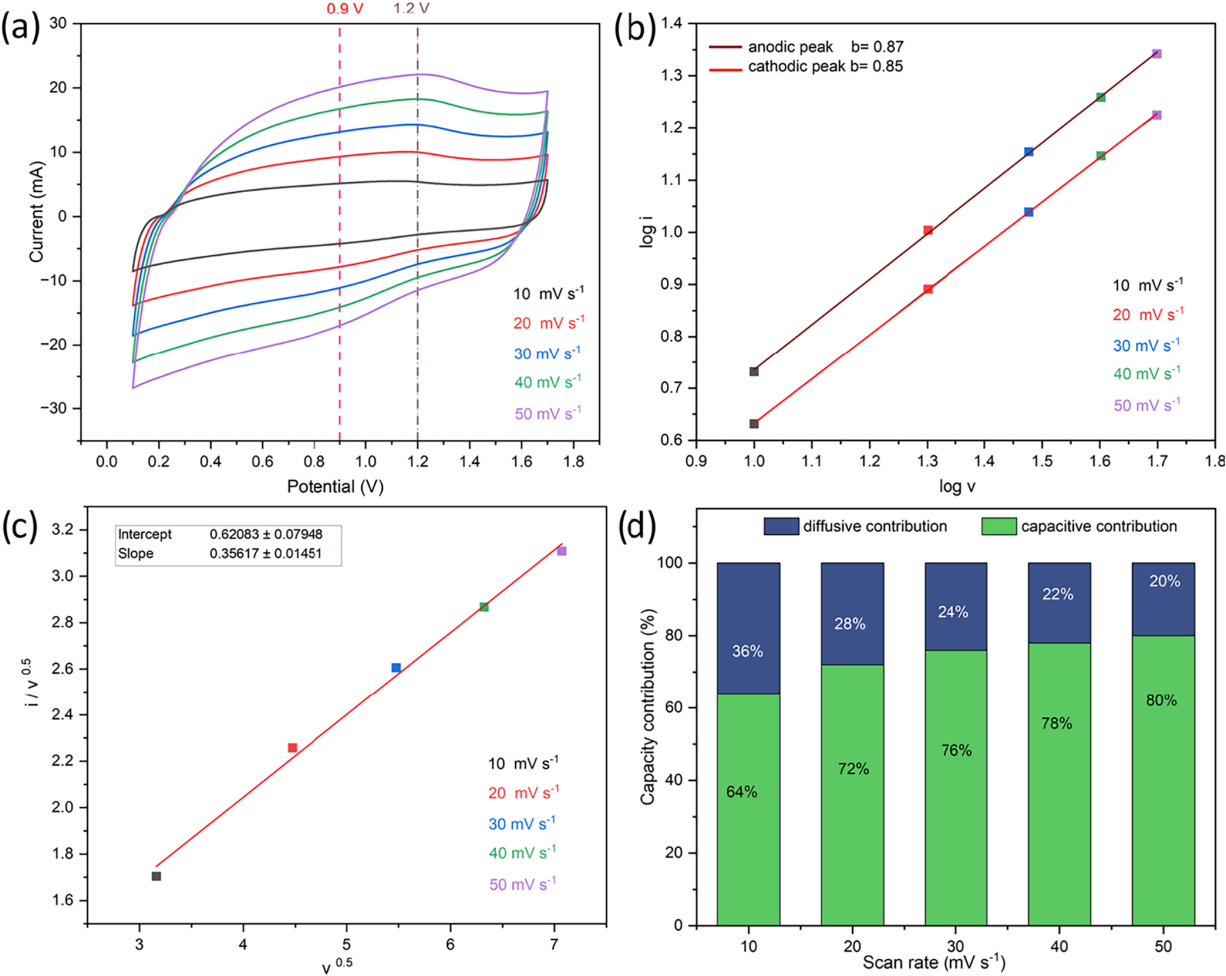
Charge storage mechanism of AT-HC14-CB: CV profiles at different scan rates (a), log­(*i*) vs log­(*v*) plots (b), Dunn′s profile (c), and capacitive and diffusion-controlled contributions at different scan rates (d).

To further probe interfacial and transport limitations, EIS measurements were performed on AT-derived carbon cathodes containing 10 wt % conductive carbon black after 2000 galvanostatic cycles at 10 A g^–1^ (charged state) in the ZTFS-based electrolyte. The resulting Nyquist plots together and the equivalent circuit employed for fitting are shown in Figure 9a (fit parameters in Table S12). The equivalent circuit used for all electrodes comprises an ohmic resistance (*R*_s_) in series with two parallel resistor-constant-phase element (*R* || *CPE*) units, followed by an open Warburg element (*W*_0_). Here, *R*_s_ primarily accounts for the electrolyte resistance and additional contributions from current collectors, contacts, and cell hardware. The two *R* || *CPE* elements are interpreted as effective descriptors of distributed interfacial and ion-transport processes with different characteristic time constants within the hierarchical porous electrode. The use of CPEs instead of ideal capacitors reflects the expected nonideal capacitive behavior of heterogeneous porous carbons. The *W*_0_ element captures the diffusion-related low-frequency response and is consistent with finite-length ion transport within a bounded pore network, rather than ideal semi-infinite diffusion.

**Figure 9:**
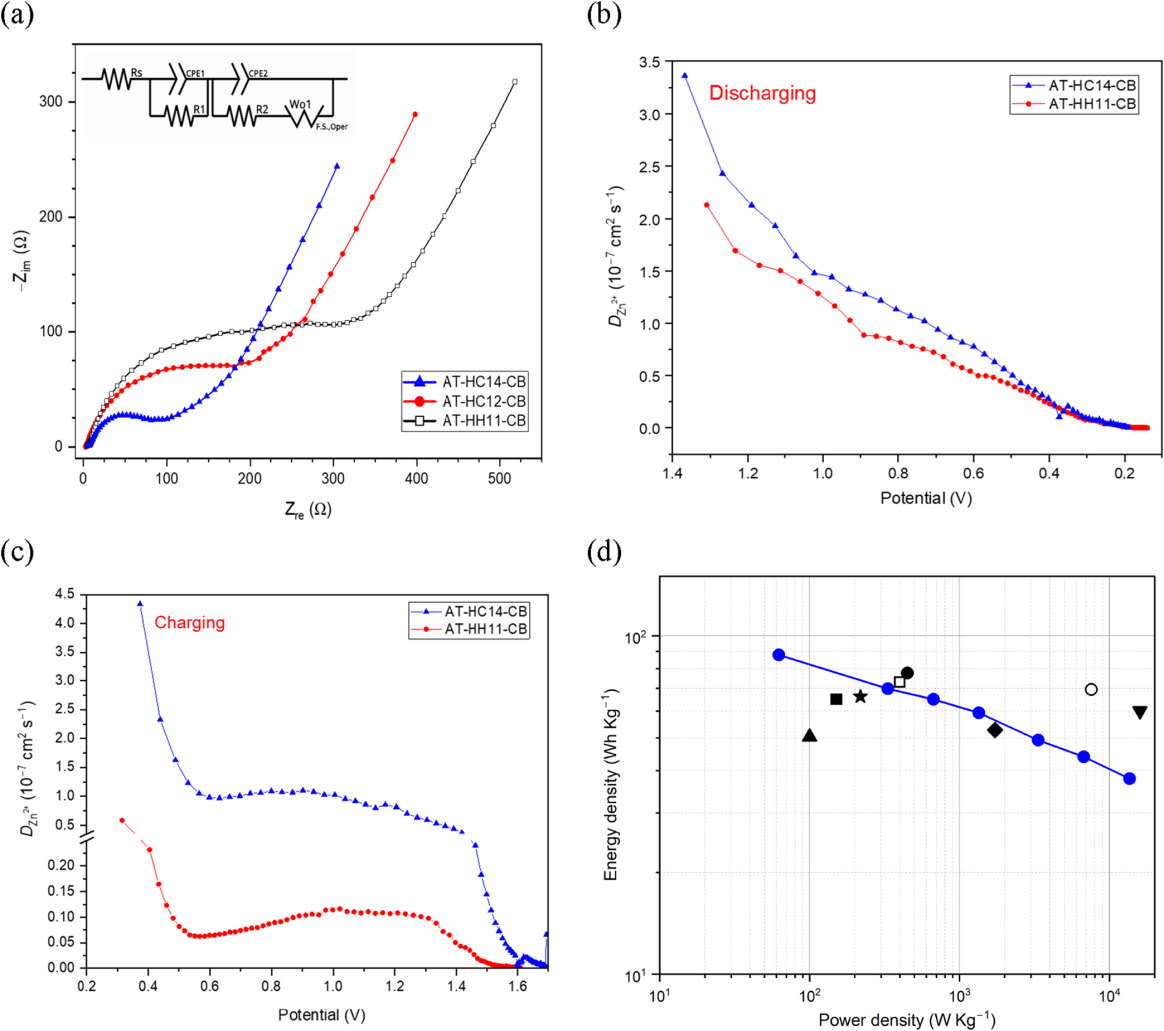
Nyquist plots of AT-HC12-CB, AT-HC14-CB, and AT-HH11-CB together with the equivalent circuit (a); apparent GITT-derived diffusion coefficients for AT-HC14-CB and AT-HH11-CB during discharge (b) and charge (c); and Ragone plot for the best-performing material reported in this work with metrics normalized to the mass of active material in the cathode (blue line). Literature data included in panel (d) are identified by the following symbols: square,^46^ circle,^51^ up triangle,^52^ down triangle,^53^ diamond,^54^ star,^55^ empty circle,^56^ and empty square.^57^

Clear differences were observed among the three cycled electrodes (AT-HC12-CB, AT-HC14-CB, and AT-HH11-CB). AT-HC14-CB exhibits the lowest overall impedance response within the explored frequency window, consistent with its lower *R*_s_ and reduced interfacial polarization relative to the other samples. In contrast, AT-HH11-CB shows systematically larger impedance, with a higher high-frequency intercept and a more pronounced midto-low frequency response. While the fitted parameters should be regarded as effective (i.e., model-dependent) descriptors rather than unique physical quantities, the comparative trend is robust: the best-performing electrode (AT-HC14-CB) displays a less resistive distributed response and a more capacitive (more vertical) low-frequency behavior, whereas AT-HC12-CB and especially AT-HH11-CB exhibit a stronger low-frequency rise indicative of more severe transport and/or polarization constraints within the porous electrode network.

To complement the EIS analysis and further assess transport kinetics within the carbon cathodes, GITT measurements were carried out during both discharge and charge for AT-HC14-CB and AT-HH11-CB (Figure 9b,c). The derived diffusion coefficients are reported as *D*_Zn_^2+^; however, in aqueous ZHSCs these values should be regarded as apparent transport coefficients, since cotransport/charge compensation by other ionic species (e.g., anions and/or protons) may contribute depending on potential and local electrolyte environment.^12^ Under the present conditions, the extracted values mainly reflect transport limitations within the porous carbon cathode rather than solid-state diffusion in the Zn anode.^58^

During discharge, the *D*_Zn_^2+^ values decrease progressively for both electrodes as the potential is lowered. Over nearly the entire discharge range, AT-HC14-CB exhibits higher *D*_Zn_^2+^ values than AT-HH11-CB, with the difference being more pronounced at higher potentials and gradually narrowing toward the end of discharge. This behavior indicates less hindered ion transport in AT-HC14-CB throughout most of the zinc-ion storage process.

During charge, both electrodes show a nonmonotonic evolution of *D*_Zn_^2+^ values, characterized by an initial sharp decrease, followed by a broader intermediate region with more moderate variation, and a final decline near the upper cutoff potential. Importantly, AT-HC14-CB maintains clearly higher *D*_Zn_^2+^ values than AT-HH11-CB over most of the charging process, again indicating faster transport kinetics and lower mass-transport limitations.

These GITT results are consistent with the EIS response. In particular, AT-HC14-CB displays a less resistive low-frequency behavior and a more vertical capacitive tail in the Nyquist plot, whereas AT-HH11-CB shows a stronger low-frequency rise, indicative of more pronounced diffusion/polarization limitations within the porous electrode. Although the *D*_Zn_^2+^ values derived from GITT and the diffusion-related contribution inferred from EIS are not directly equivalent in a strict quantitative sense, both techniques consistently indicate more favorable ion-transport characteristics for AT-HC14-CB.

Overall, the best-performing almond-tree-derived hard carbon, AT-HC14-CB, delivered an energy density of 87.8 Wh kg^–1^ at 62.3 W kg^–1^ and retained 37.9 Wh kg^–1^ even at a high-power density of 13.6 kW kg^–1^ (Figure 9d). These values were calculated by integrating the experimentally measured discharge curves (eq 3); therefore, the effect of the *iR* drop is inherently included in the reported energy and power densities, even though the voltage loss becomes significant at high current density. The resulting metrics remain comparable to representative literature reports for biomass-derived carbon cathodes in ZHSCs, including electrodes prepared using more corrosive and less sustainable activating agents as ZnCl_2_ or KOH (see Table S13).^46,51–57^

## Conclusions

This work shows that hydrothermal pretreatment (HTC) is a useful precursor-conditioning step for the preparation of biomass-derived carbon cathodes by subsequent K_2_CO_3_ activation. Under the conditions explored here, HTC-assisted activation provides a more favorable balance between porosity development and carbon preservation than direct activation, yielding carbons with a hierarchical pore network that combines extensive micro/ultramicroporosity with a meaningful mesopore contribution. The overall yield reached 19 wt % (dry basis).

The precursor nature was also found to be important. Carbons derived from almond pruning residues (AT) consistently outperformed those prepared from almond shells (AS), indicating that the initial precursor structure strongly influences the final activation response, pore development, and electrochemical behavior. Among the materials investigated, AT-HC14-CB provided the best overall performance with improved rate capability. At the same time, the galvanostatic profiles revealed a significant *iR* drop at the highest current density, showing that resistive losses remain relevant under extreme high-rate operation.

Long-term cycling and post-mortem characterization demonstrated that electrolyte chemistry is decisive for durability. Compared with ZnSO_4_, the ZTFS electrolyte delivered higher sustained capacity and better retention upon prolonged cycling. Post-mortem XRD, SEM-EDS, and XPS analyses indicate that ZnSO_4_ promotes the formation of abundant crystalline hydroxy-sulfate byproducts on both electrodes, whereas ZTFS favors a less crystalline fluorine-containing interphase with more limited deposition. The carbon cathode preserved its morphology and thickness after cycling, supporting its structural integrity under repeated charge/discharge.

Finally, the kinetic analyses are mutually consistent: CV indicates a strong surface-controlled contribution to charge storage, whereas EIS and GITT point to lower overall transport limitations for the best-performing electrode. Overall, this study provides a practical process route to valorize almond-derived residues into competitive cathode materials for aqueous Zn-ion hybrid supercapacitors, while also identifying clear next steps focused on HTC optimization, electrode formulation, higher-loading validation, and electrolyte engineering.

## Supplementary Material



## Notes

Biomass and hydrochar characterization; additional physicochemical characterization of the carbons; Raman analysis and fitted parameters; XPS survey spectra and high-resolution deconvolutions; CHN elemental analysis; adsorption isotherms and pore size distributions; additional electrochemical data, including two- vs three-electrode comparison and higher-loading tests; post-mortem characterization of electrodes cycled in ZTFS and ZnSO_4_ electrolytes (XRD, SEM-EDS, and XPS); self-discharge analysis; GCD profiles with *iR* drop; EIS fitting parameters; and literature benchmarking table (PDF)
