# Supplementary material for: Hierarchically Porous Carbons from Almond Residues via Hydrothermal Pretreatment and Mild K2CO3 Activation for Aqueous Zinc Hybrid Supercapacitors
Source: Energy Fuels. 2026 Apr 16;40(17):9699–714. doi: 10.1021/acs.energyfuels.6c00654 (PMC13137416; doi:10.1021/acs.energyfuels.6c00654)
Supplement: Supplementary file 1 [file ef6c00654_si_001.pdf]

Supporting Information

Hierarchically Porous Carbons from Almond Residues via Hydrothermal Pretreatment and Mild K_2CO_3 Activation for Aqueous Zinc Hybrid Supercapacitors

Densa A. Shaj[†,‡], Darío Alvira[†,‡], Daniel Antorán[†,‡], Víctor Sebastián[‡,§,ǀ,□], Joan J. Manyà[†,‡,]*

[†] Aragón Institute for Engineering Research (I3A), Thermochemical Processes Group, University of Zaragoza, Escuela Politécnica Superior, Crta. de Cuarte s/n, 22071 Huesca, Spain.

[‡] Department of Chemical Engineering and Environmental Technologies, University of Zaragoza, Campus Río Ebro, María de Luna 3, 50018 Zaragoza, Spain.

[§] Instituto de Nanociencia y Materiales de Aragón (INMA), CSIC-Universidad de Zaragoza, Zaragoza, Spain.

[ǀ] Networking Research Center on Bioengineering Biomaterials and Nanomedicine (CIBER-BBN), Madrid, Spain.

[□] Laboratorio de Microscopías Avanzadas, Universidad de Zaragoza, 50018, Zaragoza, Spain.

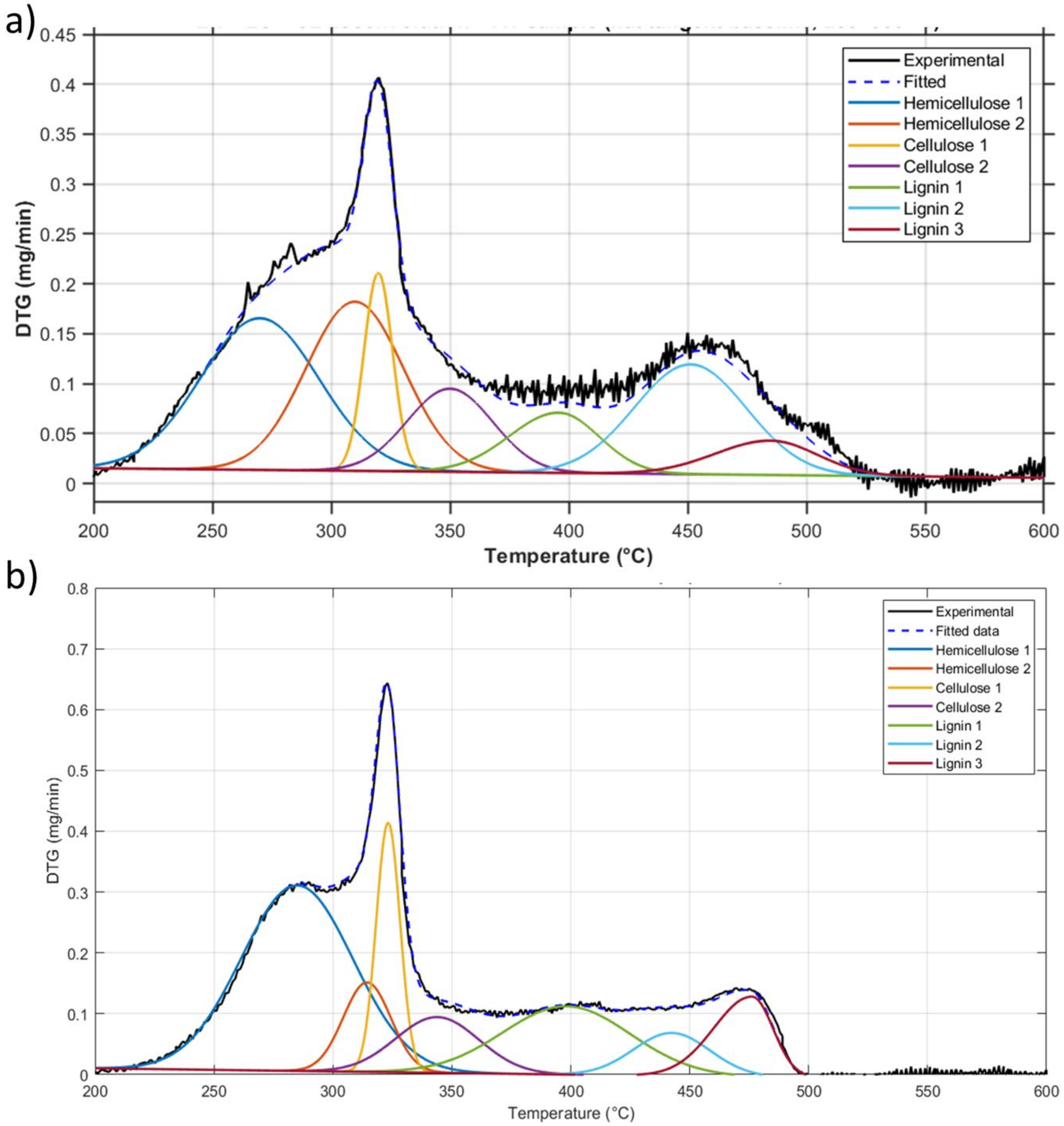

Figure S1. Deconvolution of DTG profiles obtained for AT (a) and AS (b) samples.

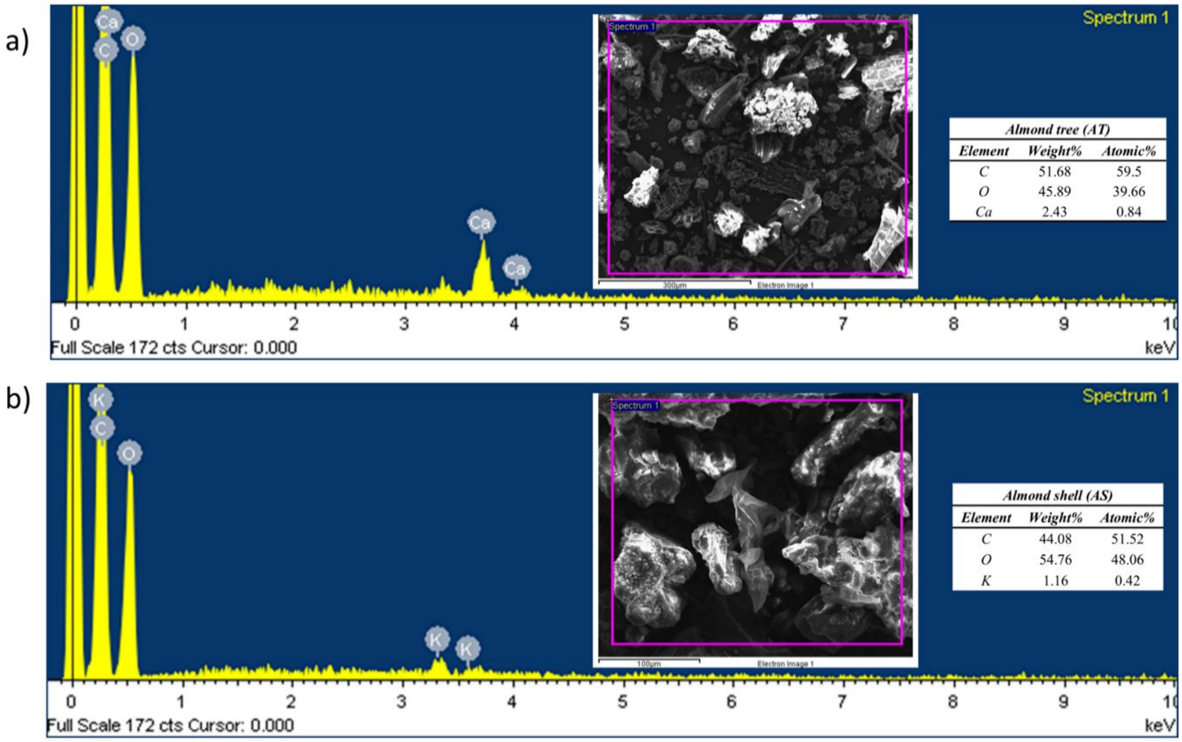

Figure S2. EDX spectra of raw biomasses: AT (a) and AS (b).

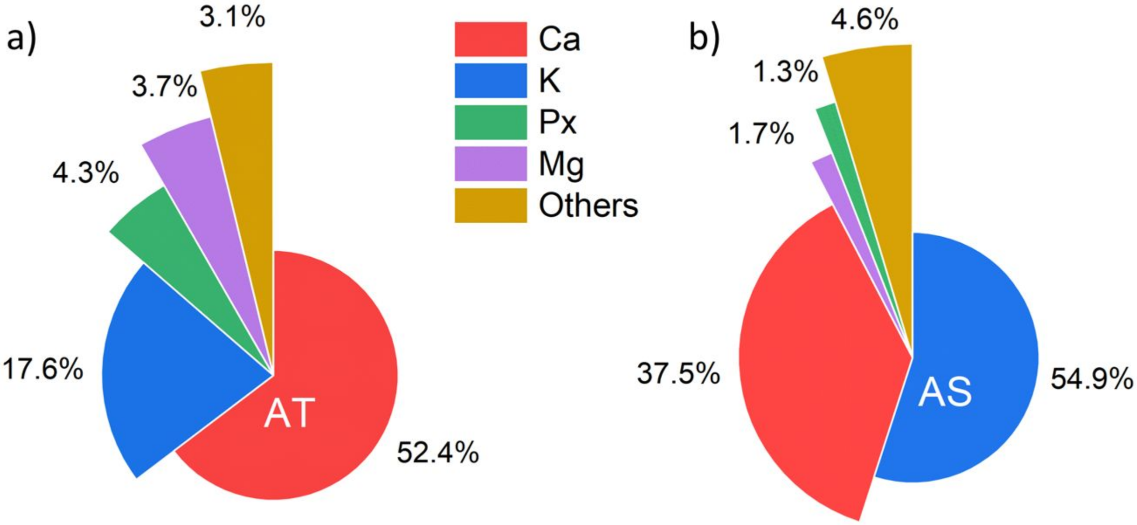

Figure S3. XRF analysis of inorganic main components (in wt %, without normalization) for AT (a) and AS (b) raw biomasses.

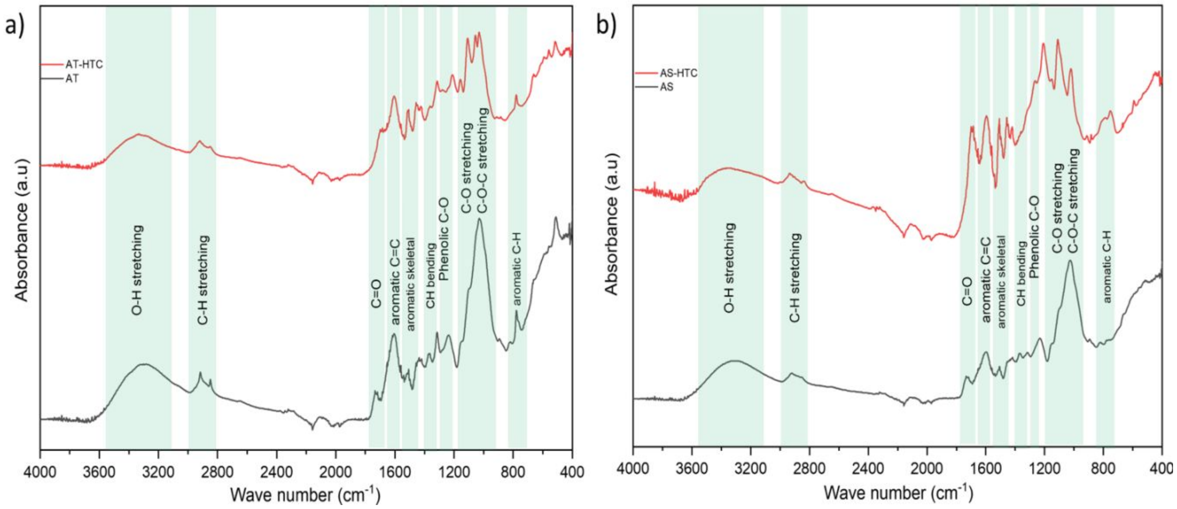

Figure S4. FTIR analysis of raw biomass and hydrochar (HTC) for AT and AT-HTC (a), and AS and AS-HTC (b). Peak assignments were made based on literature reports.[1,2]

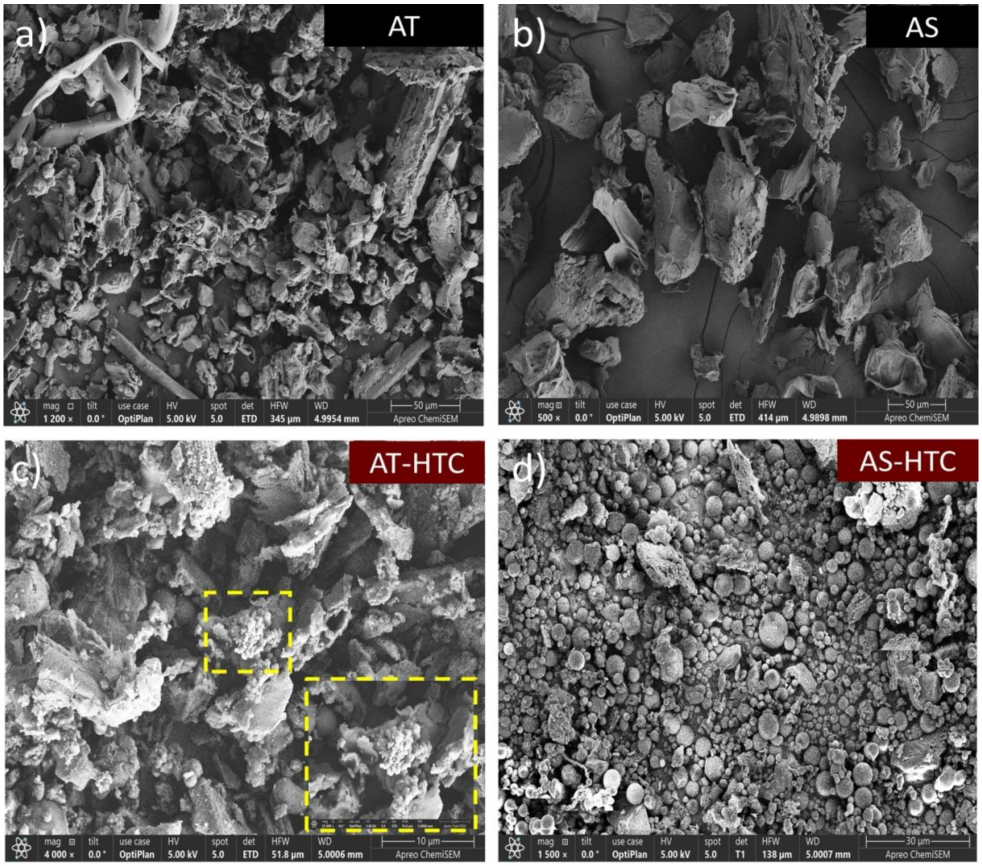

Figure S5. SEM images of AT (a) and AS (b) raw biomasses as well as AT-HTC (c) and AS-HTC (d) hydrochars.

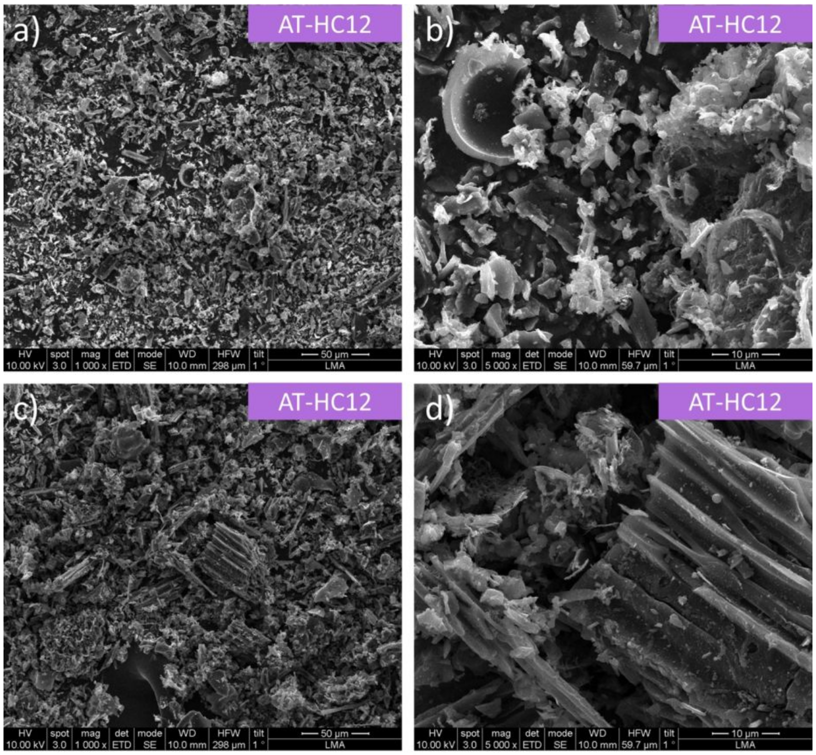

Figure S6. Additional SEM micrographs of AT-HC12.

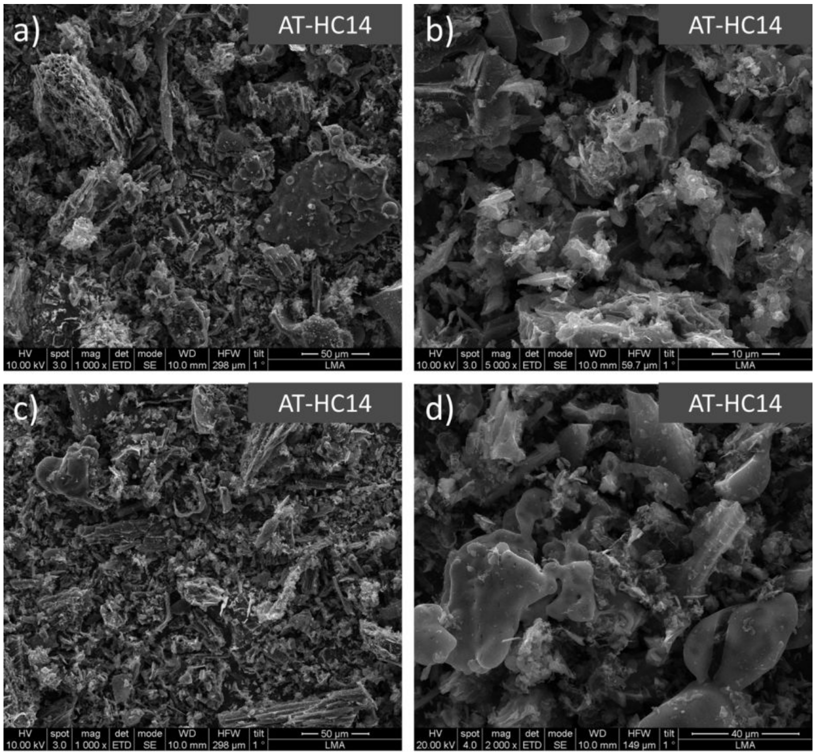

Figure S7. Additional SEM micrographs of AT-HC14.

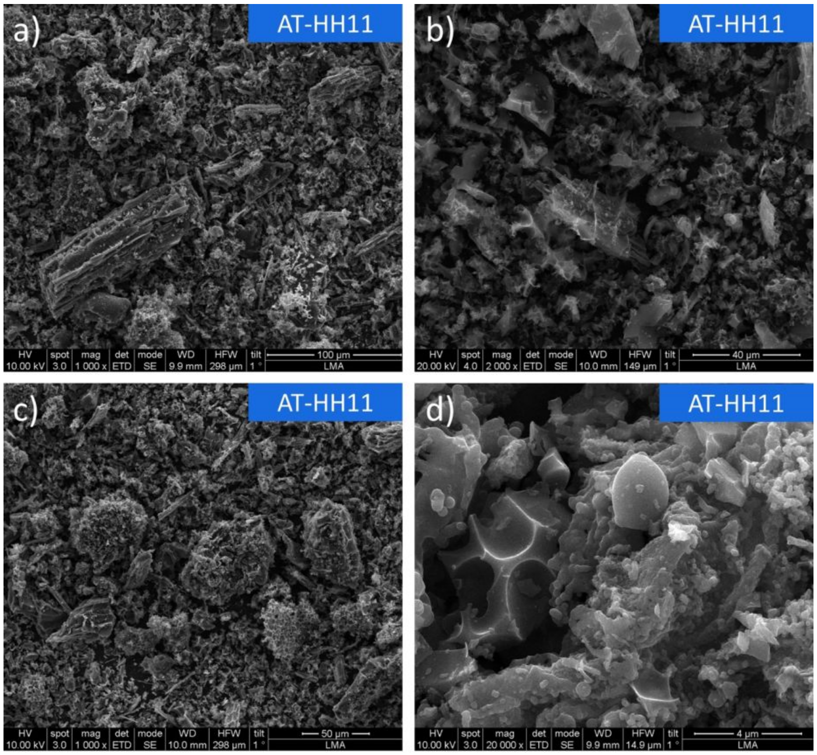

Figure S8. Additional SEM micrographs of AT-HH11.

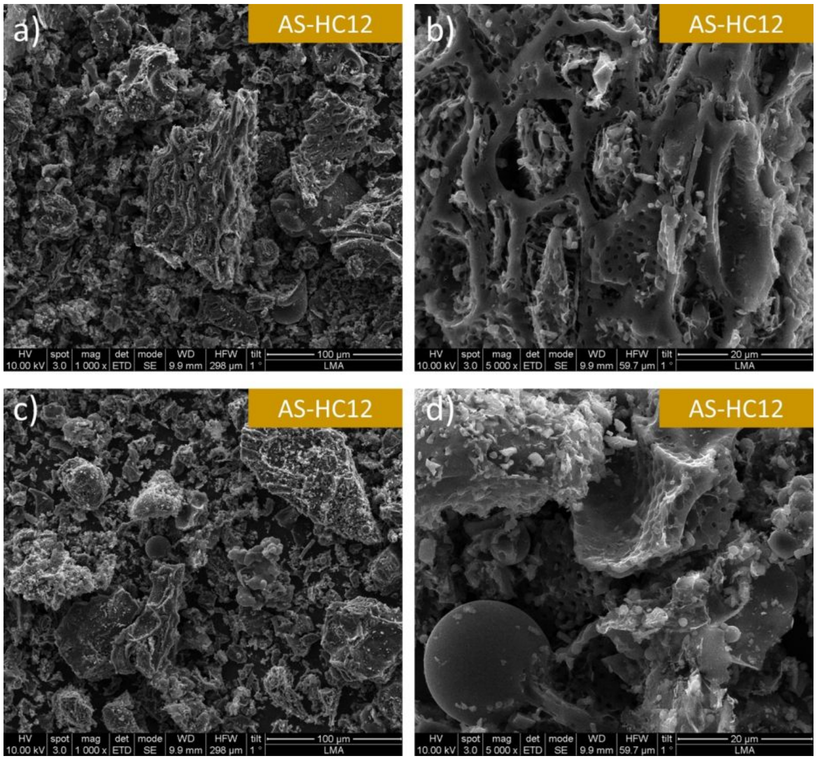

Figure S9. Additional SEM micrographs of AS-HC12.

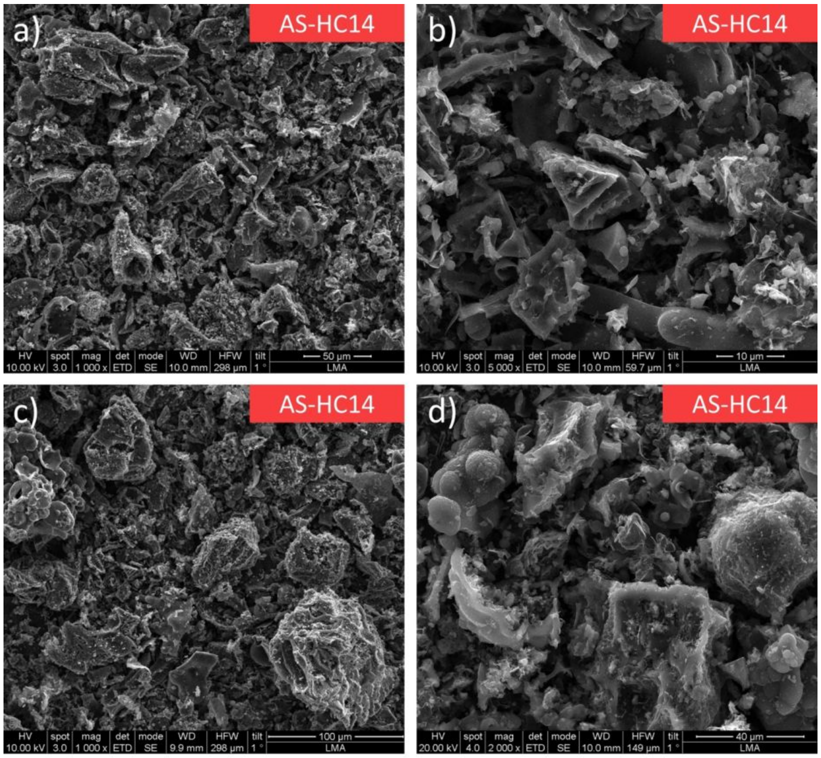

Figure S10. Additional SEM micrographs of AS-HC14.

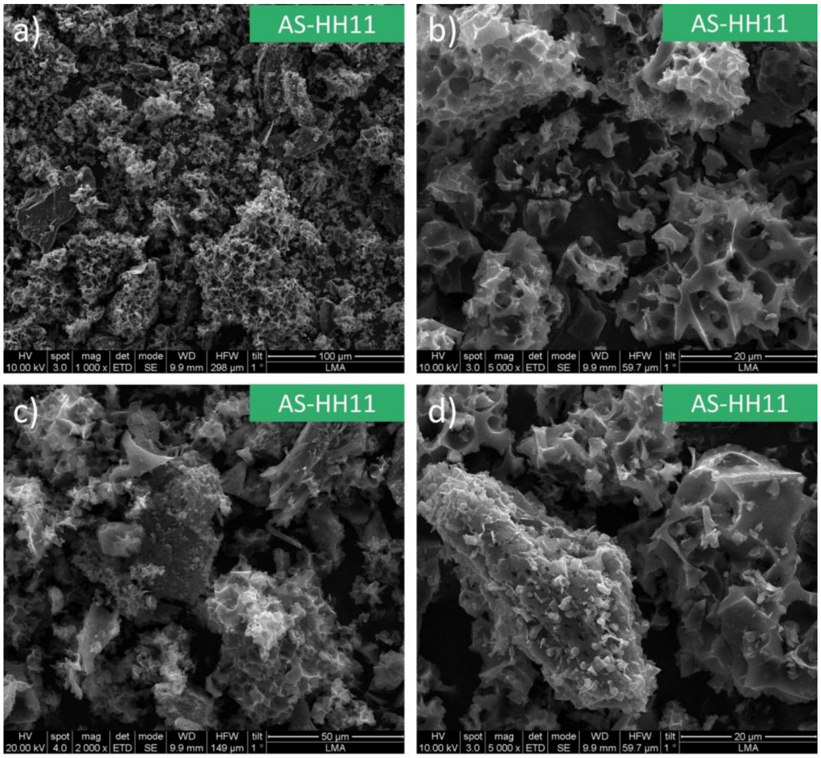

Figure S11. Additional SEM micrographs of AS-HH11.

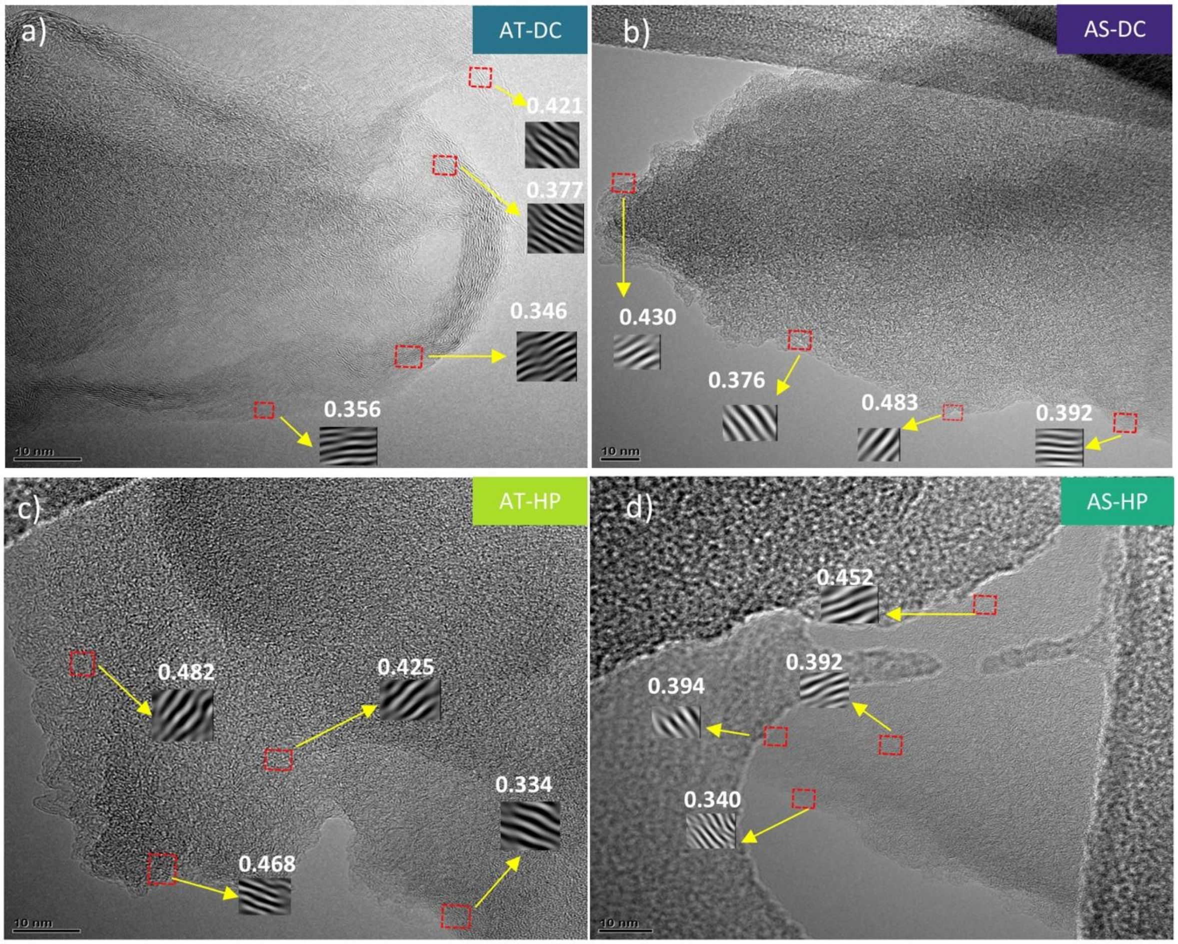

Figure S12. HR-TEM images of AT-DC (a), AS-DC (b), AT-HP (c), and AS-HP (d).

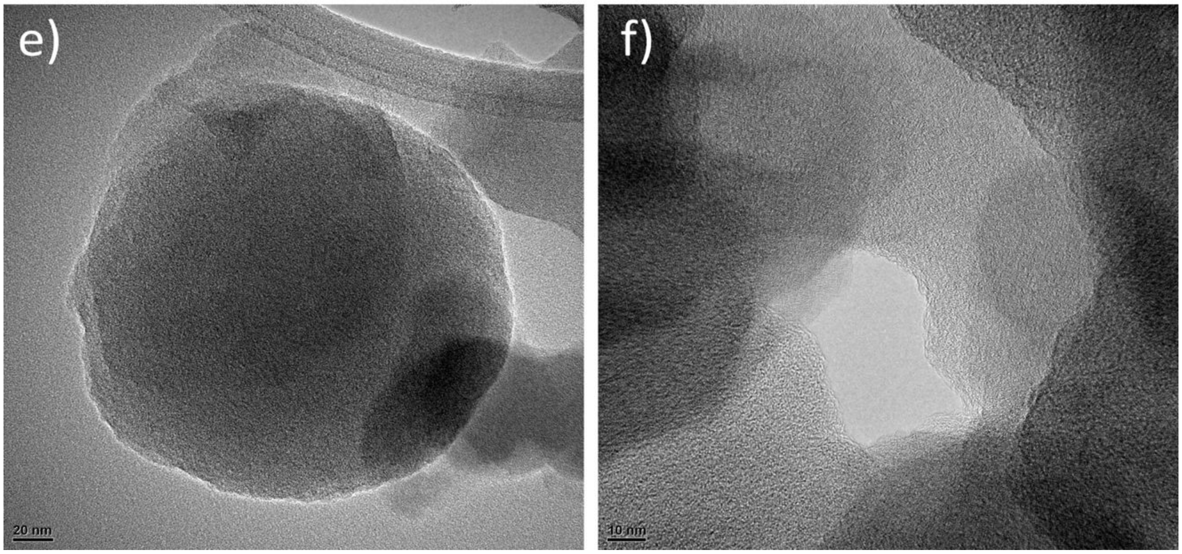

Figure S13. Additional HR-TEM images of AT-HC12.

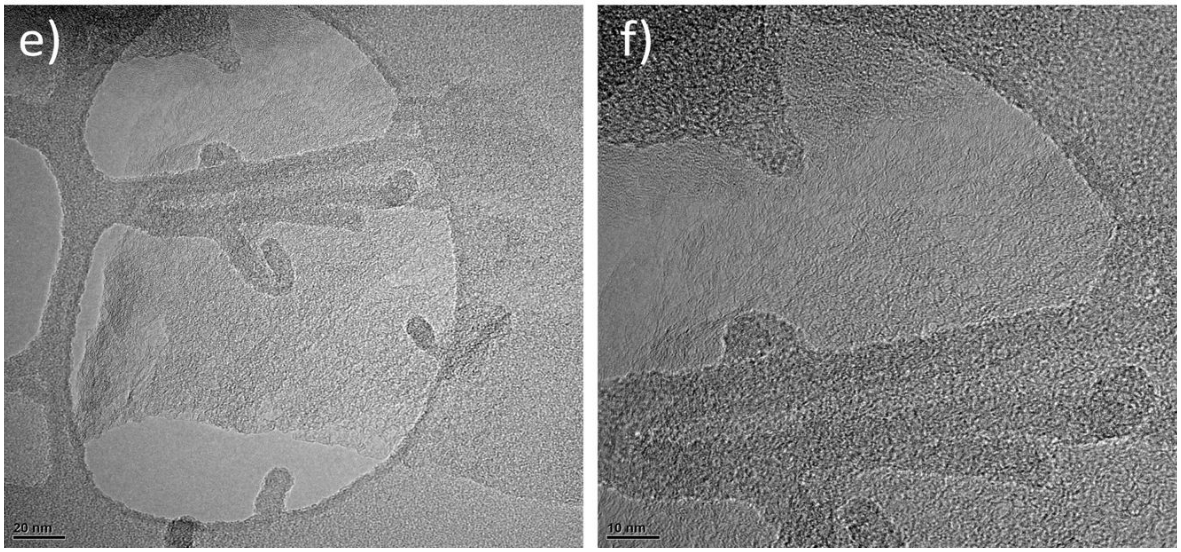

Figure S14. Additional HR-TEM images of AT-HC14.

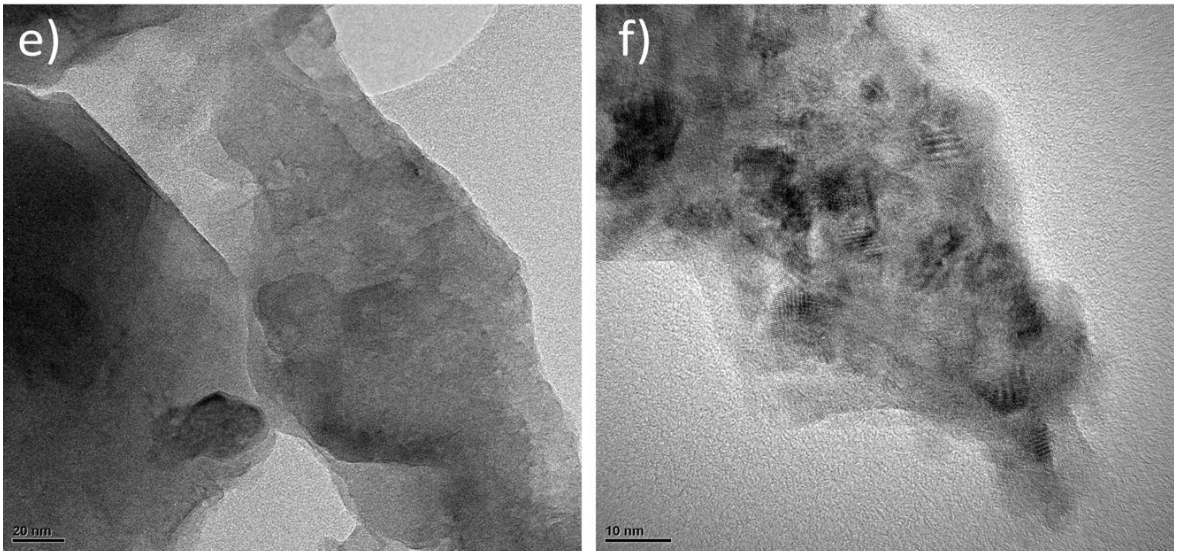

Figure S15. Additional HR-TEM images of AT-HH11.

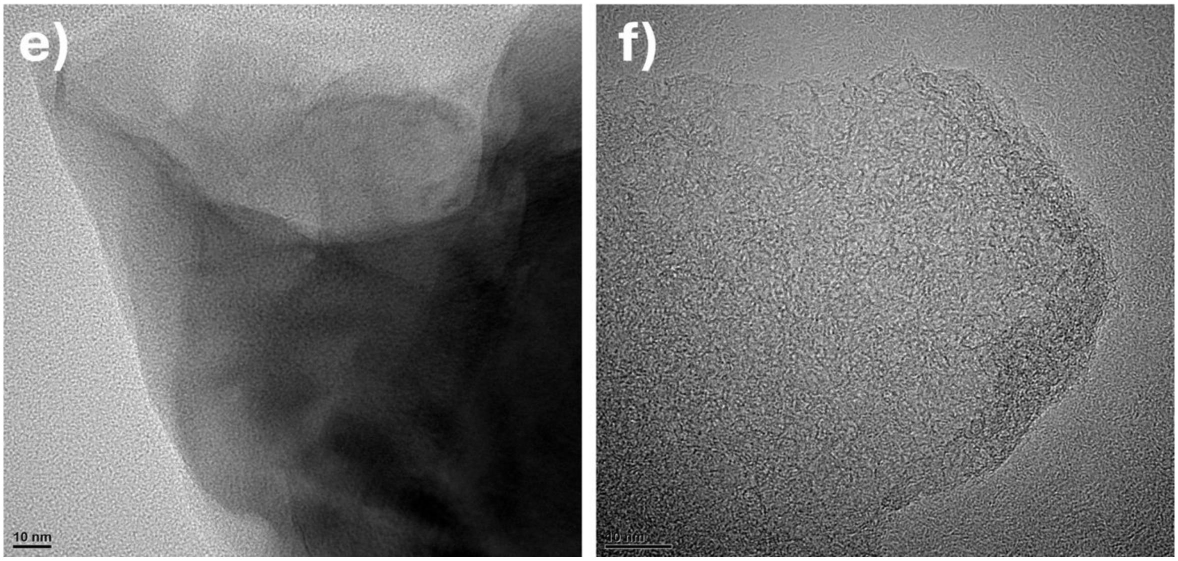

Figure S16. Additional HR-TEM images of AS-HC12.

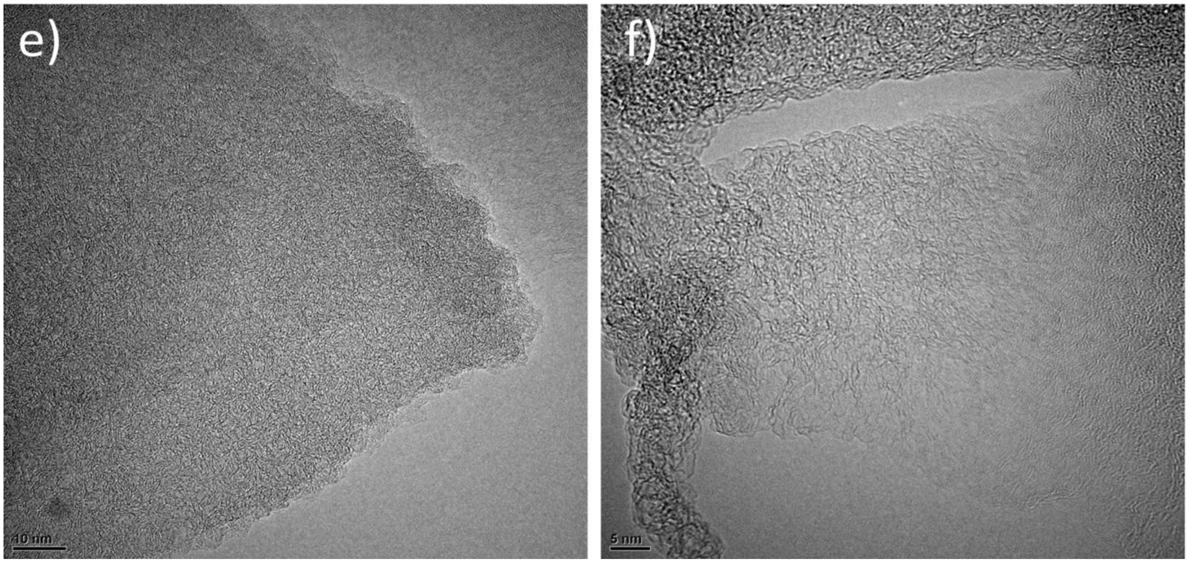

Figure S17. Additional HR-TEM images of AS-HC14.

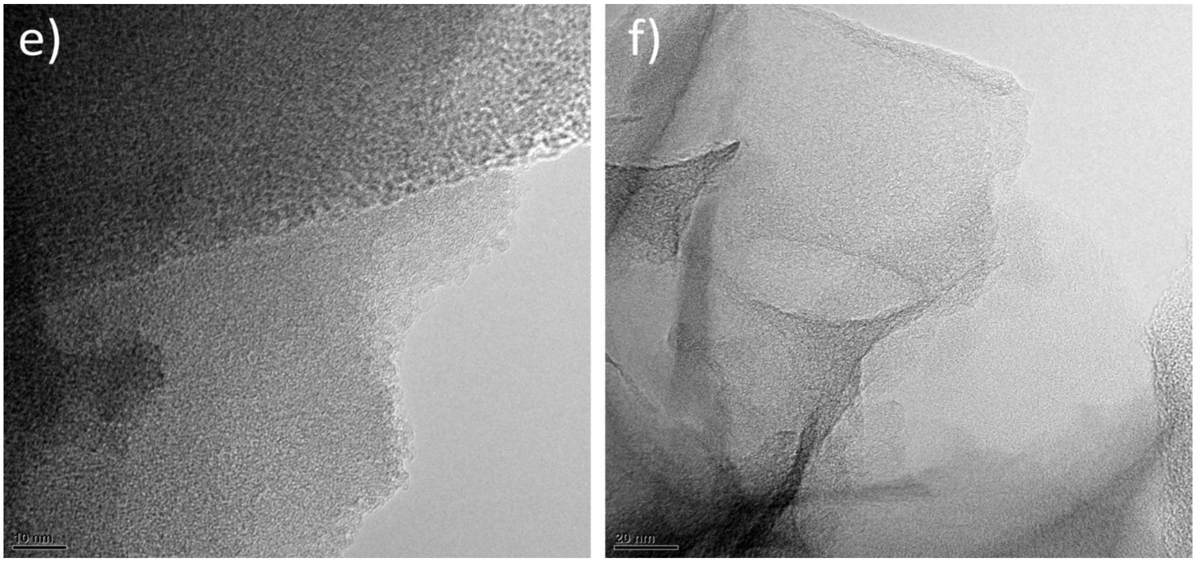

Figure S18. Additional HR-TEM images of AS-HH11.

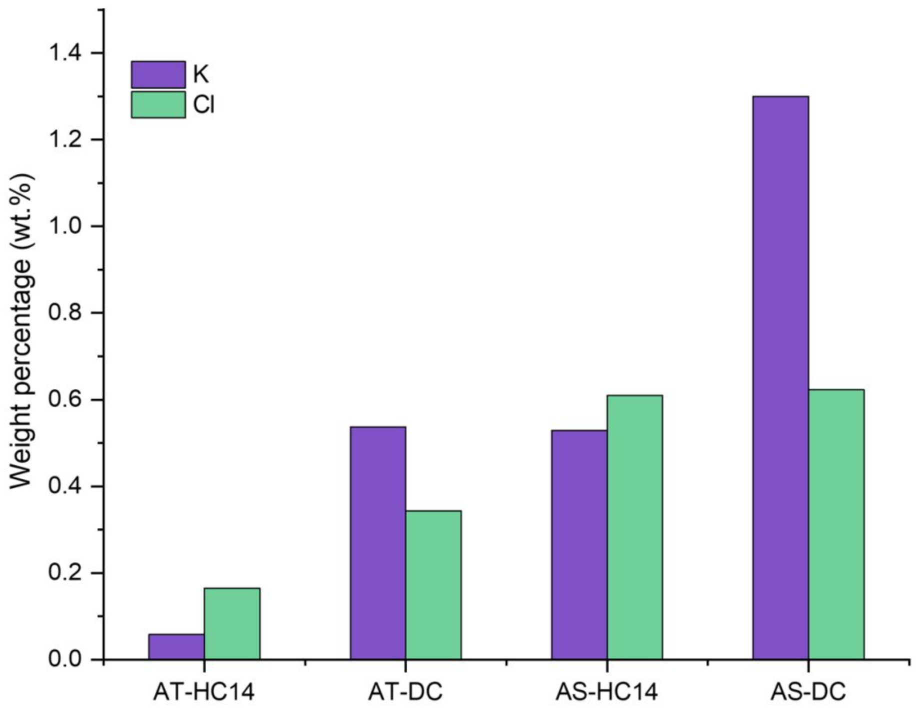

Figure S19. KCl contents measured by XRF for AT-HC14, AT-DC, AS-HC14, and AS-DC samples (wt %).

Table S1. SEM-EDS quantification of residual KCl in AT-HC14 and AS-HC14 samples

element	AT-HC14		AS-HC14	
	at %	wt %	at %	wt %
C	92.1	89.4	94.2	92.1
O	7.8	10.1	5.6	7.3
K	0.0	0.1	0.1	0.2
Cl	0.1	0.4	0.1	0.4

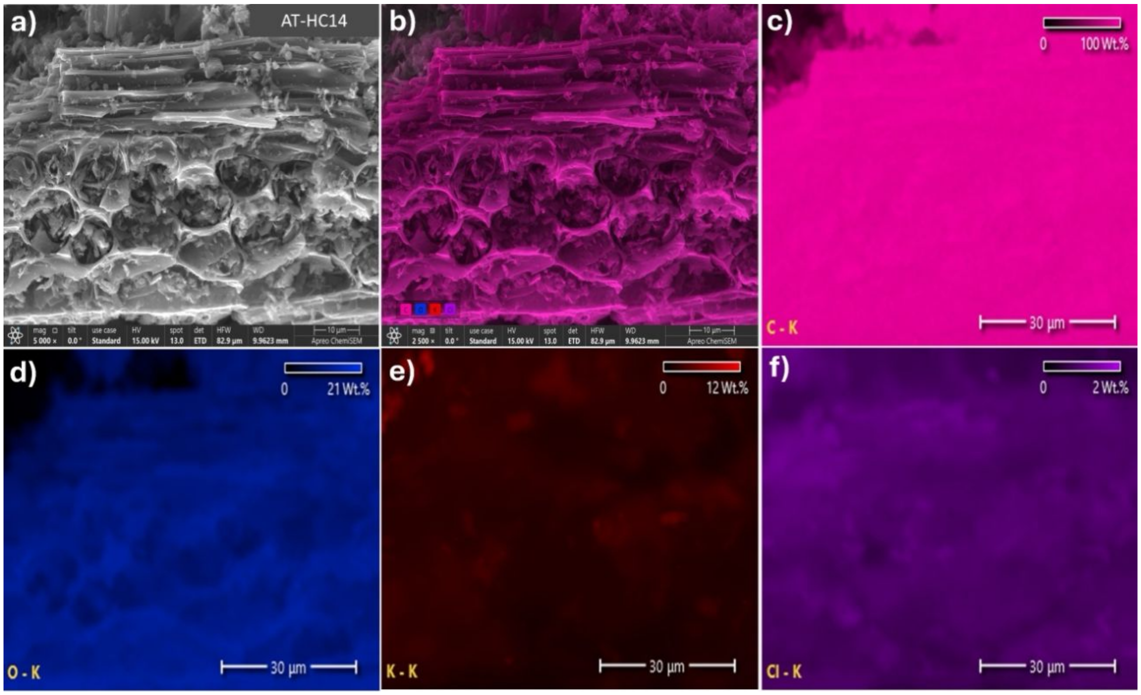

Figure S20. SEM-EDS elemental mapping of AT-HC14: SEM image (a), elemental quantification map (b), and spatial distributions of C (c), O (d), K (e), and Cl (f).

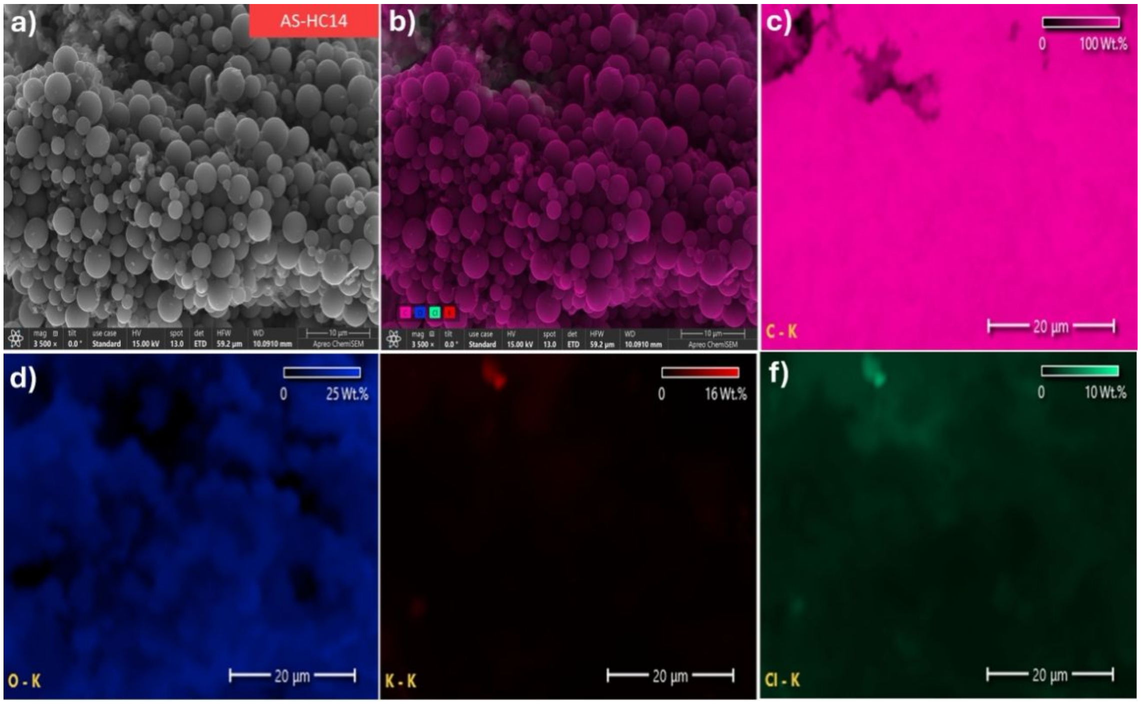

Figure S21. SEM-EDS elemental mapping of AS-HC14: SEM image (a), elemental quantification map (b), and spatial distributions of C (c), O (d), K (e), and Cl (f).

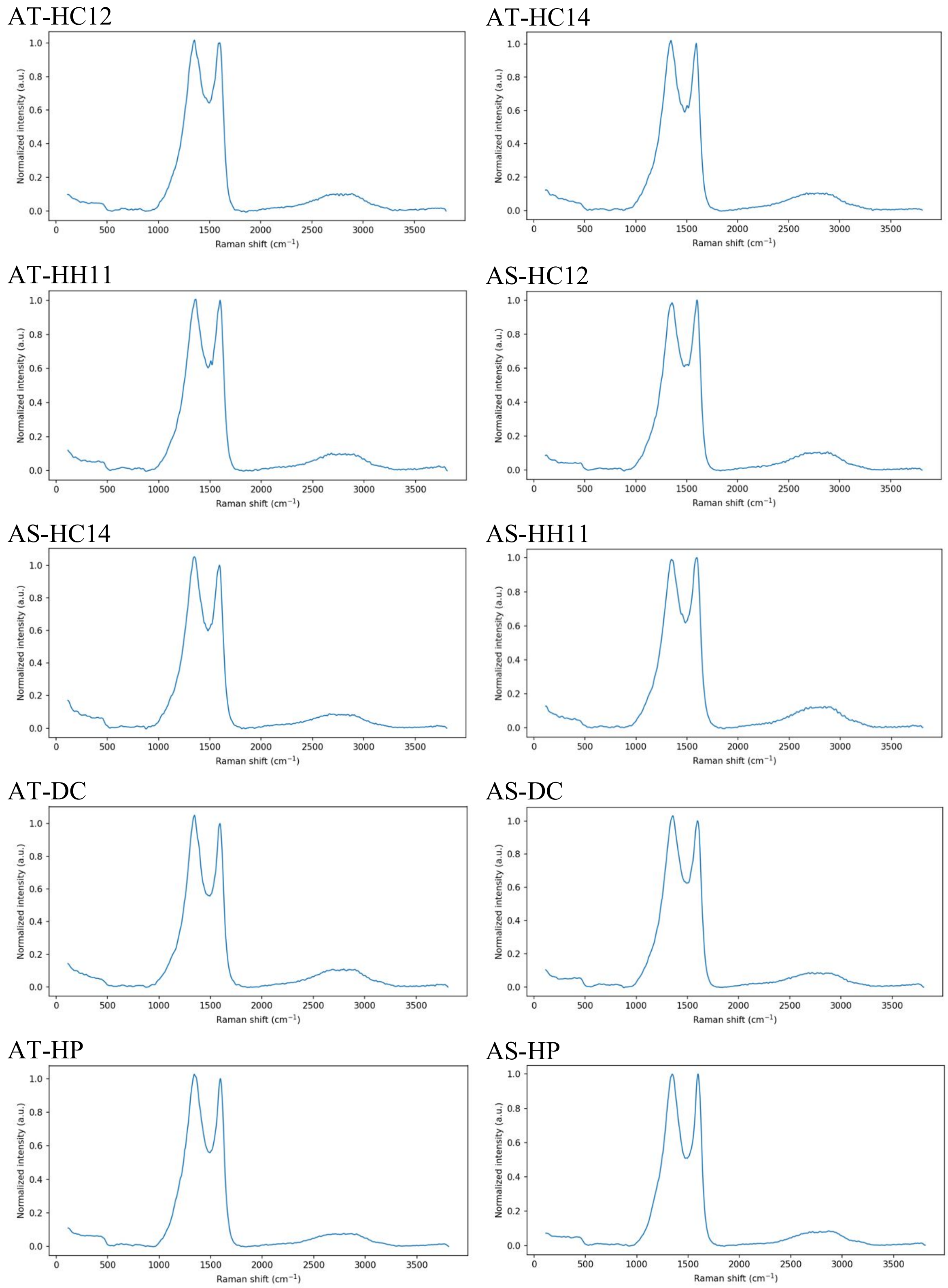

Figure S22. Selected Raman spectra (baseline subtracted and normalized) of carbons.

Table S2. Parameters obtained from Raman spectra (four measurements per sample).

sample ID	ω_G (cm^{-1})	ω_D (cm^{-1})	$FWHM_G$ (cm^{-1})	$FWHM_D$ (cm^{-1})	I_D/I_G ratio
AT-HC12	1601 ± 2.13	1348 ± 2.04	85.63 ± 4.39	181.7 ± 17.3	1.088 ± 0.03
AT-HC14	1596 ± 1.40	1344 ± 3.07	84.60 ± 2.22	187.7 ± 8.32	1.124 ± 0.04
AT-HH11	1600 ± 2.69	1349 ± 2.32	89.57 ± 1.56	196.8 ± 10.3	1.096 ± 0.02
AS-HC12	1599 ± 3.51	1348 ± 1.29	86.99 ± 1.98	192.2 ± 8.79	1.105 ± 0.01
AS-HC14	1597 ± 2.70	1348 ± 1.13	90.82 ± 1.37	195.9 ± 5.62	1.135 ± 0.04
AS-HH11	1596 ± 2.09	1347 ± 3.41	89.95 ± 5.42	196.2 ± 14.3	1.120 ± 0.02
AT-DC	1601 ± 3.82	1348 ± 3.64	85.60 ± 7.41	185.3 ± 16.5	1.121 ± 0.03
AS-DC	1599 ± 4.04	1349 ± 2.66	89.56 ± 2.27	187.0 ± 8.40	1.131 ± 0.02
AT-HP	1598 ± 2.76	1347 ± 2.09	83.88 ± 2.07	188.9 ± 8.70	1.074 ± 0.04
AS-HP	1598 ± 1.50	1344 ± 0.99	77.01 ± 1.54	178.7 ± 4.08	1.037 ± 0.02

Table S3. EDS analysis results (at %)

sample ID	C	O	Cl	K
AT-HC12	87.8	12.2	–	–
AT-HC14	90.3	9.71	–	–
AT-HH11	87.2	12. 8	–	–
AS-HC12	87.4	11.2	0.74	0.74
AS-HC14	88.5	9.88	0.87	0.63
AS-HH11	86.4	12.6	0.53	0.49
AT-DC	89.3	9.08	0.82	0.58
AS-DC	87.8	11.4	0.38	0.49
AT-HP	93.9	5.61	0.47	–
AS-HP	93.3	6.43	0.31	–

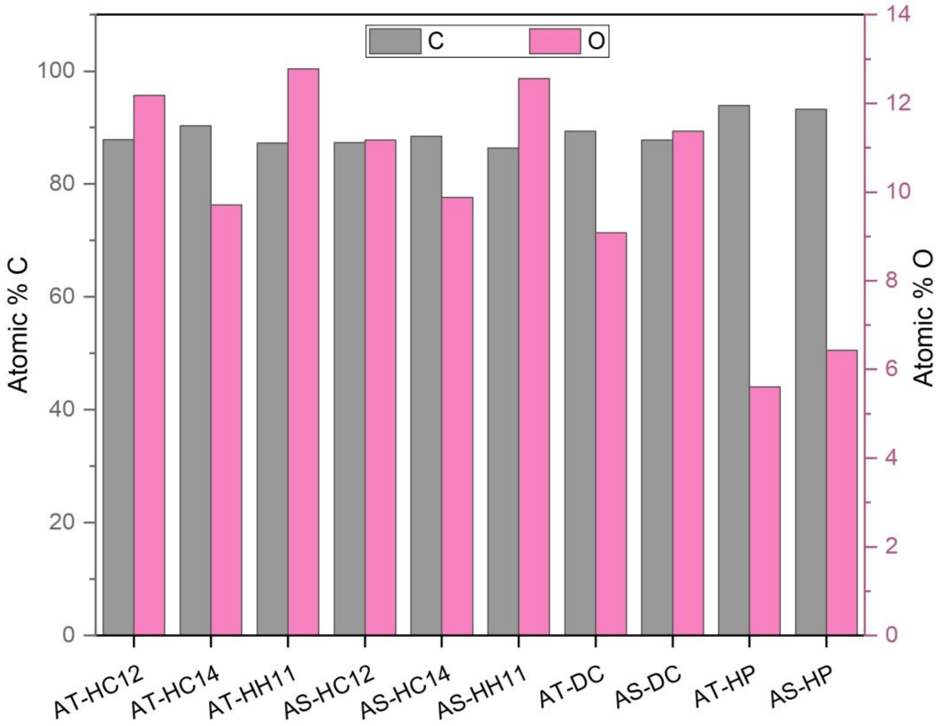

Figure S23. Carbon and oxygen contents (at %) from EDS analysis.

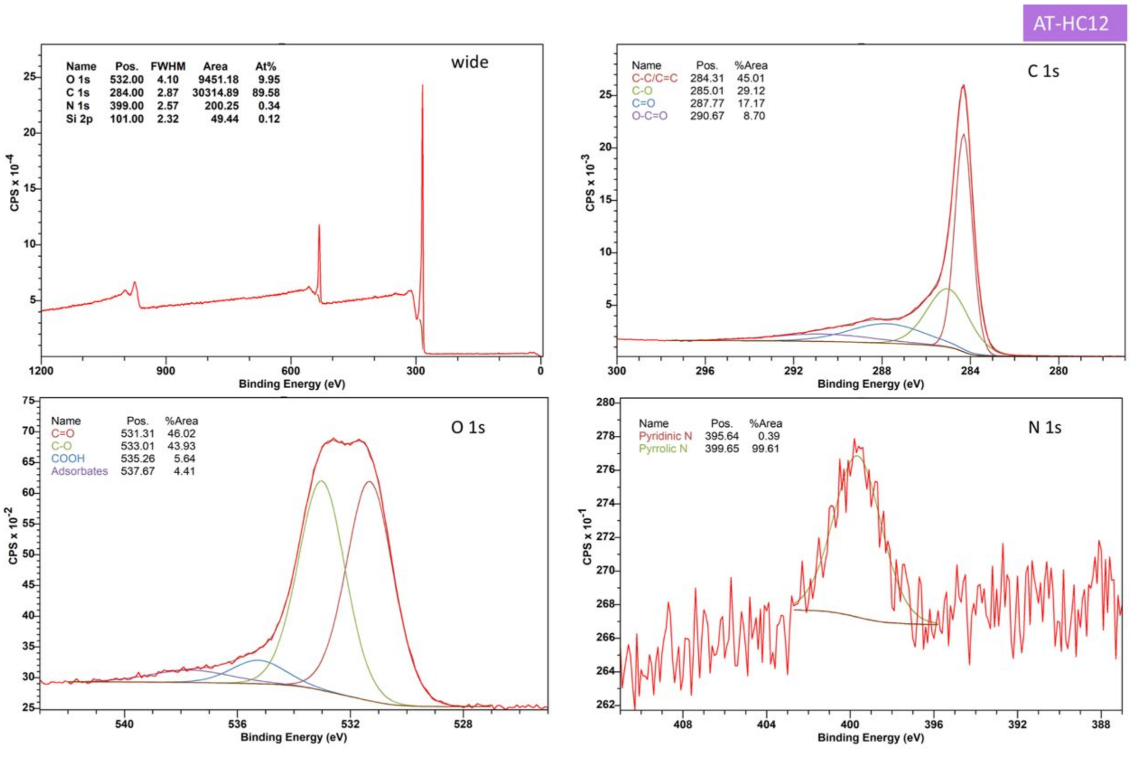

Figure S24. XPS survey, C 1s, O 1s, and N 1s spectra for AT-HC12.

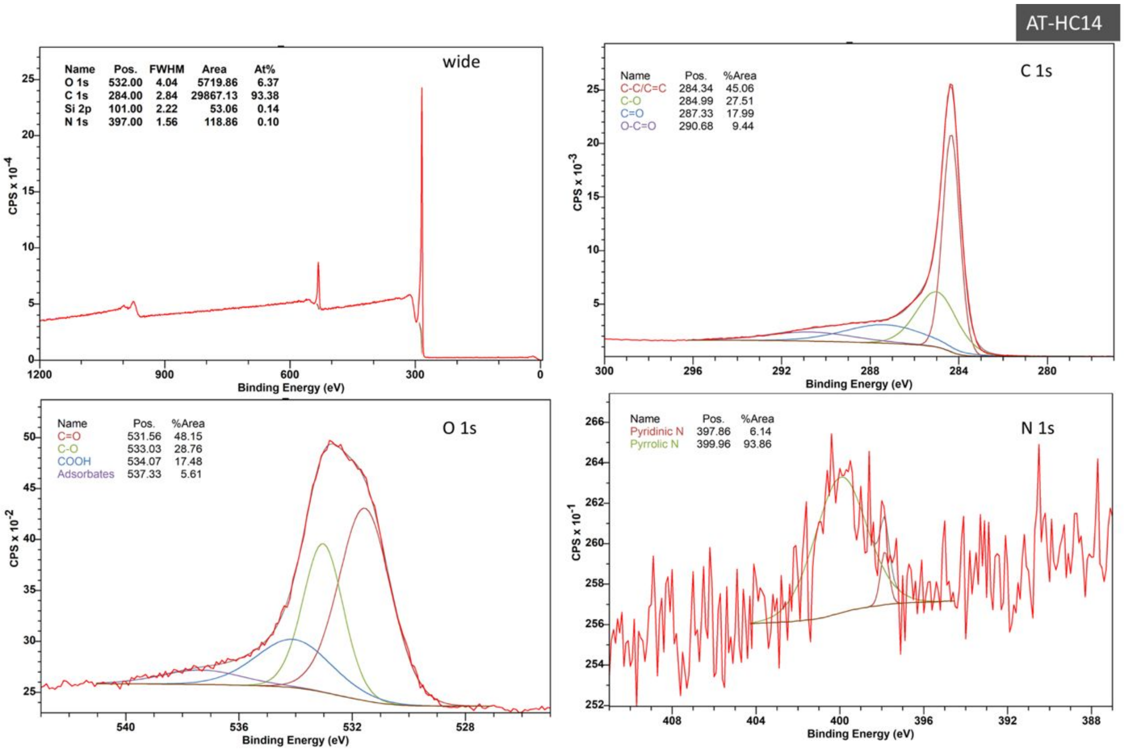

Figure S25. XPS survey, C 1s, O 1s, and N 1s spectra for AT-HC14.

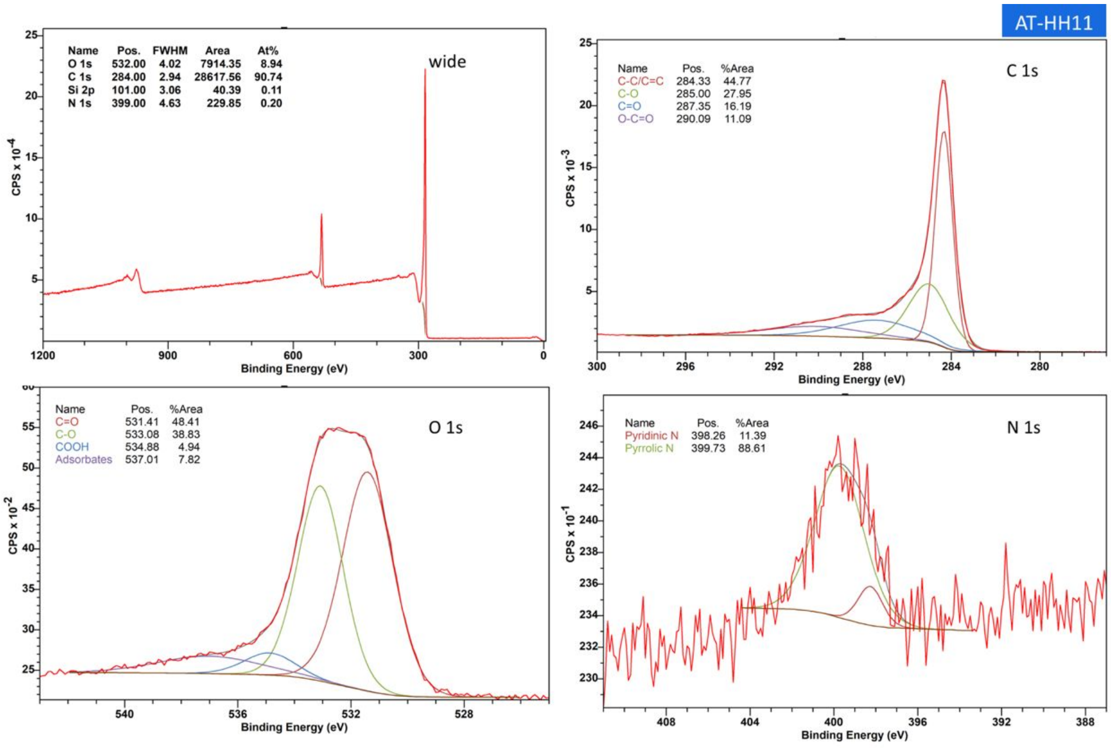

Figure S26. XPS survey, C 1s, O 1s, and N 1s spectra for AT-HH11.

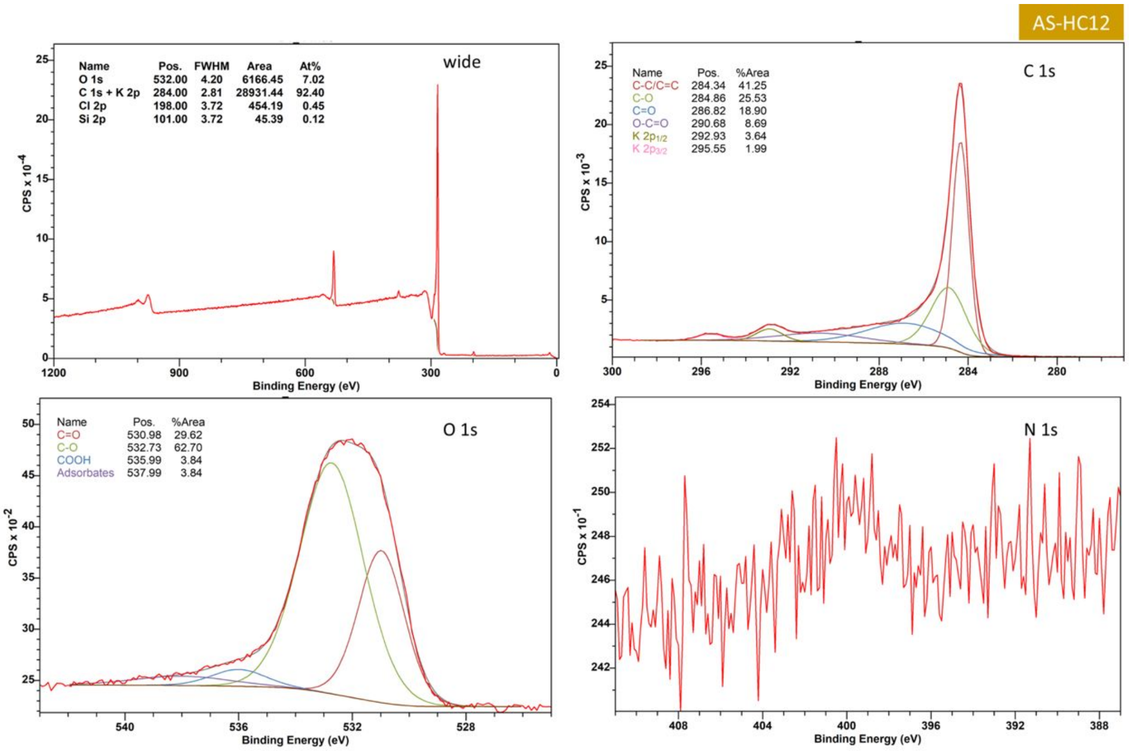

Figure S27. XPS survey, C 1s, O 1s, and N 1s spectra for AS-HC12.

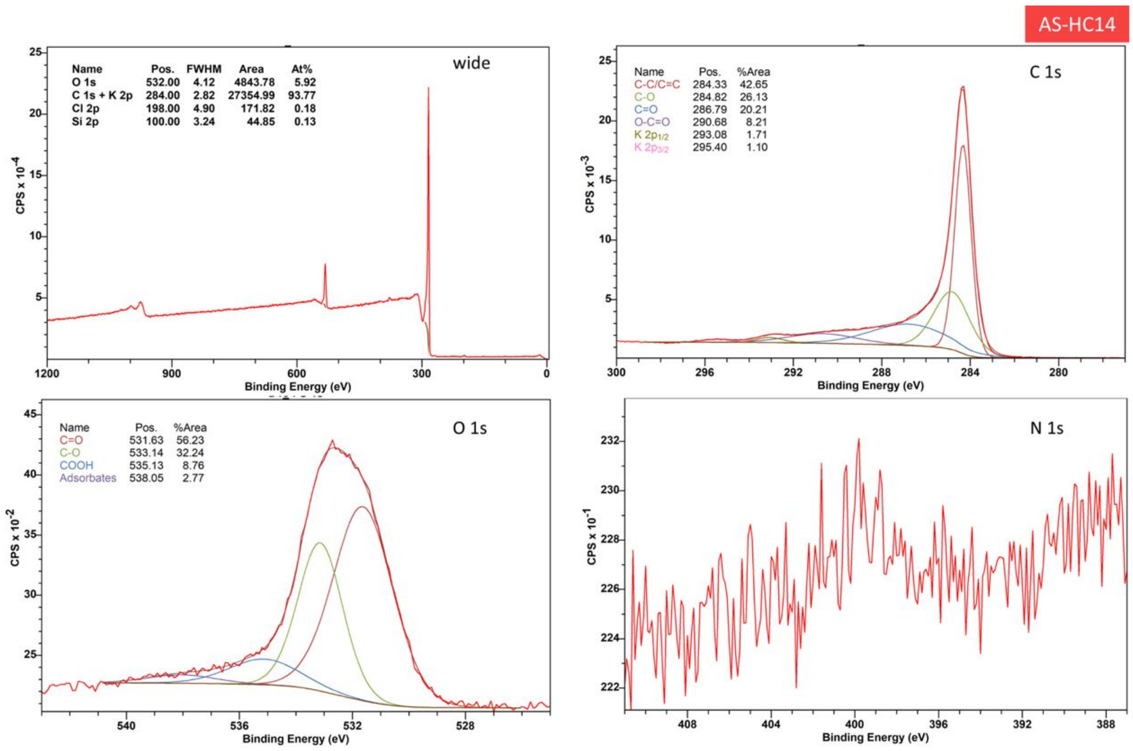

Figure S28. XPS survey, C 1s, O 1s, and N 1s spectra for AS-HC14.

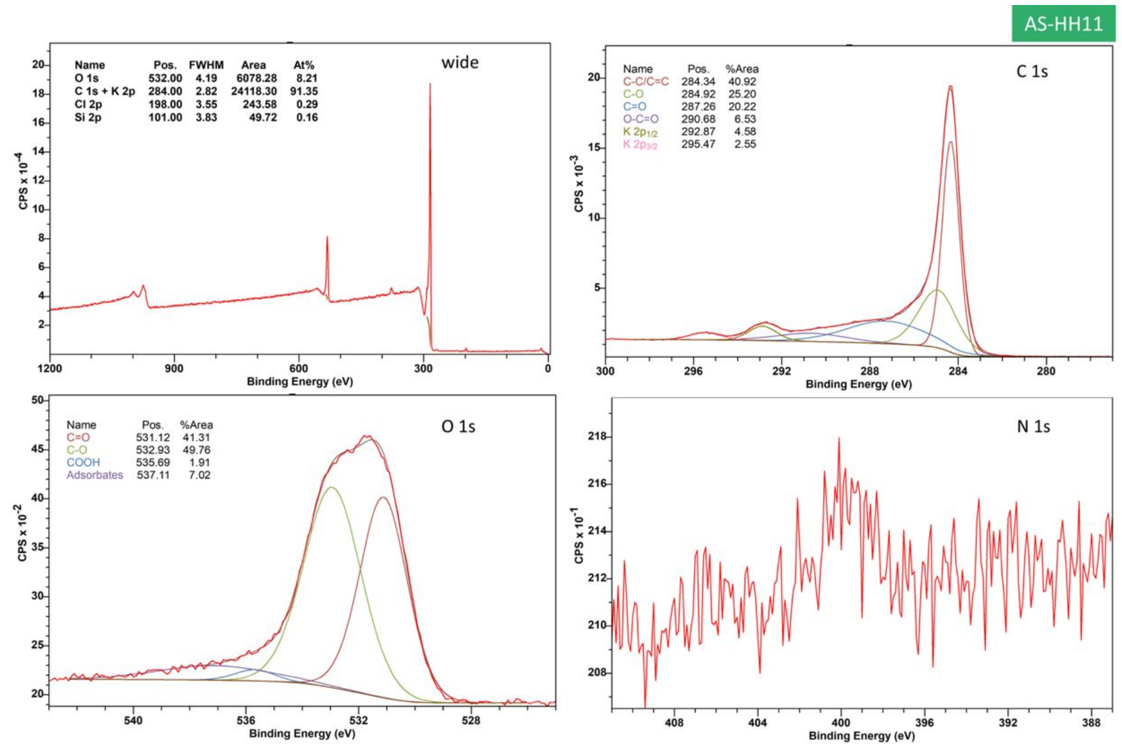

Figure S29. XPS survey, C 1s, O 1s, and N 1s spectra for AS-HH11.

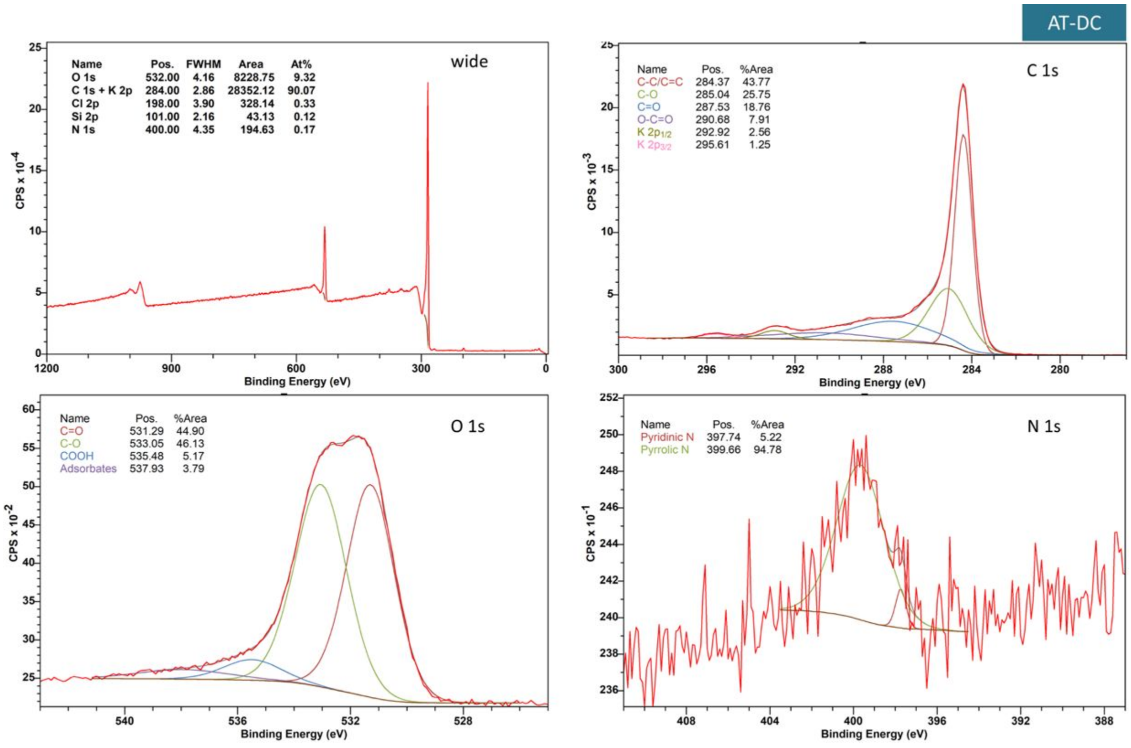

Figure S30. XPS survey, C 1s, O 1s, and N 1s spectra for AT-DC.

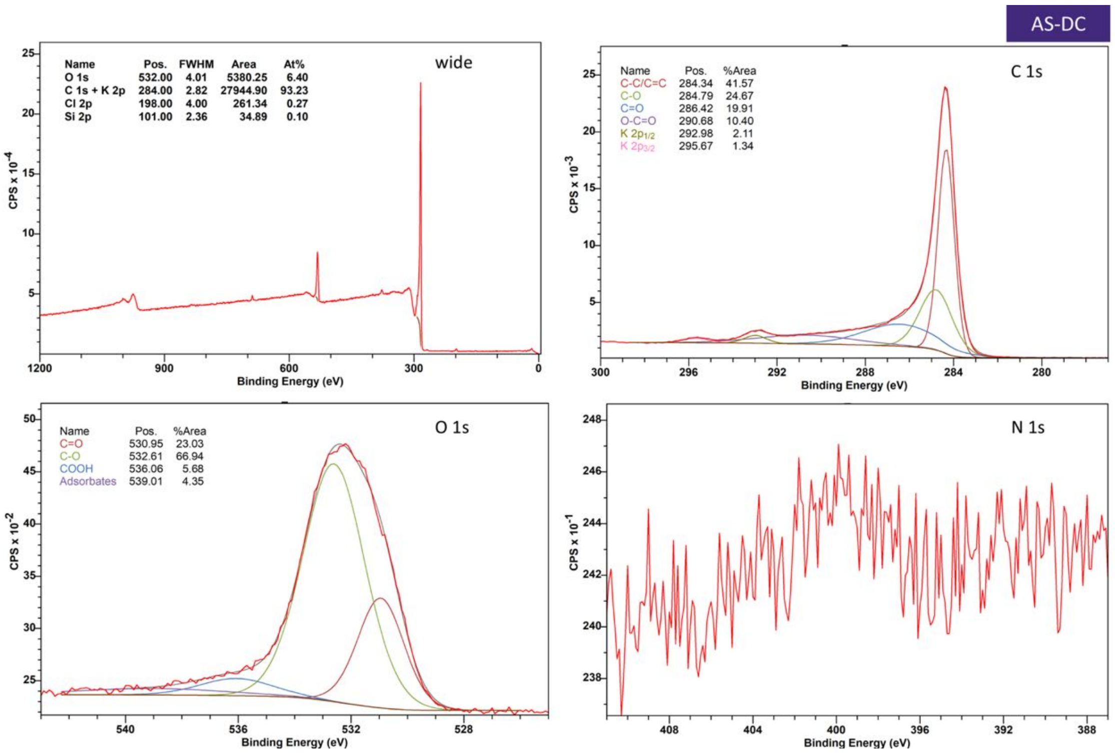

Figure S31. XPS survey, C 1s, O 1s, and N 1s spectra for AS-DC.

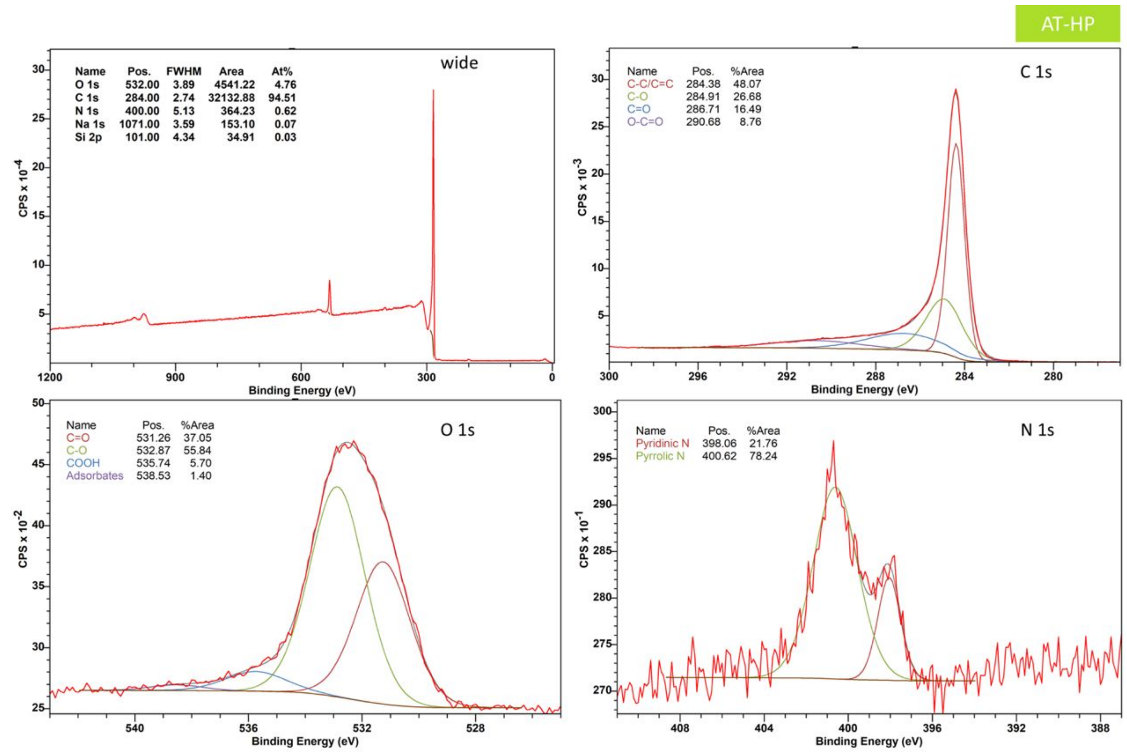

Figure S32. XPS survey, C 1s, O 1s, and N 1s spectra for AT-HP.

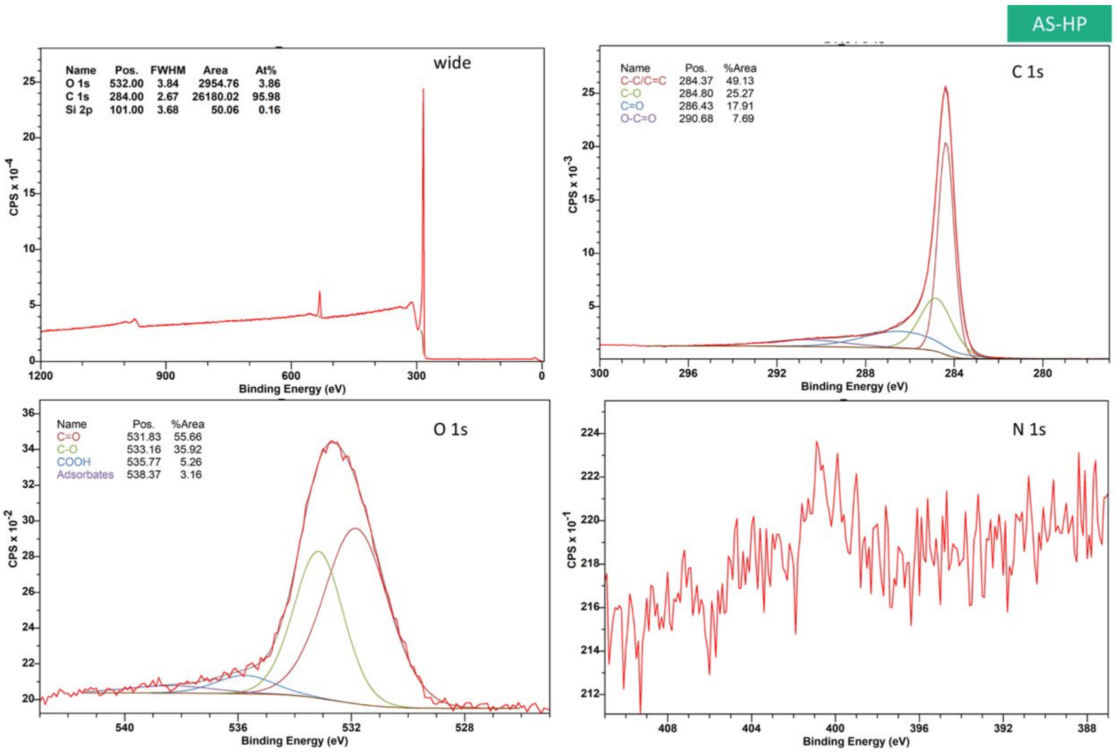

Figure S33. XPS survey, C 1s, O 1s, and N 1s spectra for AS-HP.

Table S4. Elemental composition from XPS survey spectra (at %)

sample ID	C 1s	O 1s	N 1s	K 2p	Cl 2p
AT-HC12	89.6	9.95	0.34	–	–
AT-HC14	93.4	6.37	0.10	–	–
AT-HH11	90.7	8.94	0.20	–	–
AS-HC12	87.2	7.02	–	5.20	0.45
AS-HC14	91.1	5.92	–	2.63	0.18
AS-HH11	84.8	8.21	–	6.51	0.29
AT-DC	86.6	9.32	0.17	3.43	0.33
AS-DC	90.0	6.40	–	3.22	0.27
AT-HP	94.5	4.76	0.62	–	–
AS-HP	96.0	3.86	–	–	

Table S5. Elemental composition from CHN analysis of produced BCs (wt %)

sample ID	C	H	N	O
AT-HC12	83.11 ± 0.26	1.33 ± 0.21	0.74 ± 0.01	14.83 ± 0.33
AT-HC14	86.77 ± 0.12	1.29 ± 0.15	0.85 ± 0.01	11.09 ± 0.19
AT-HH11	85.27 ± 0.02	1.32 ± 0.04	0.82 ± 0.04	12.60 ± 0.06
AS-HC12	83.90 ± 0.51	1.37 ± 0.08	0.54 ± 0.11	14.19 ± 0.53
AS-HC14	84.00 ± 0.59	1.50 ± 0.21	0.50 ± 0.07	14.00 ± 0.63
AS-HH11	81.86 ± 0.23	2.22 ± 1.08	0.67 ± 0.21	15.25 ± 1.12
AT-DC	82.14 ± 0.77	1.54 ± 0.20	0.87 ± 0.02	15.45 ± 0.80
AS-DC	83.72 ± 1.04	1.40 ± 0.11	0.36 ± 0.07	14.51 ± 1.05
AT-HP	88.00 ± 0.18	1.39 ± 0.09	1.19 ± 0.03	9.42 ± 0.20
AS-HP	93.78 ± 0.09	1.13 ± 0.04	0.62 ± 0.00	4.47 ± 0.10

Table S6. Specific surface areas and pore volumes of produced carbons

sample ID	specific surface area ($m^2\ g^{-1}$)[a]	total pore volume ($cm^3\ g^{-1}$)[a]	QSDFT surface area ($m^2\ g^{-1}$)[b]	BET N_2 surface area ($m^2\ g^{-1}$)[c]	BET CO_2 surface area ($m^2\ g^{-1}$)[d]
AT-HC12	2251	0.7837	1370	1351	1119
AT-HC14	2264	0.8461	1400	1461	1156
AT-HH11	2165	0.8332	1356	1494	1153
AS-HC12	1462	0.4900	704.9	718.4	719.0
AS-HC14	1660	0.5651	845.8	921.4	822.6
AS-HH11	1974	0.6708	1158	1171	961.8
AT-DC	1985	0.7024	1159	1208	980.9
AS-DC	1223	0.3920	489.1	509.0	636.2
AT-HP	611.3	0.1760	81.73	74.08	434.4
AS-HP	744.9	0.2143	193.8	199.6	460.6

[a] Calculated from combined CO_2 adsorption isotherm (cumulative surface area and pore volume estimated with the Monte Carlo model until 0.7 nm) and N_2 adsorption isotherm (cumulative surface area and pore volume estimated with the QSDFT model).

[b] Cumulative surface area from N_2 adsorption isotherm calculated with the QSDFT model.

[c,d] Surface areas according to the BET model from both N_2 adsorption ([c]) and CO_2 adsorption ([d]) isotherms.

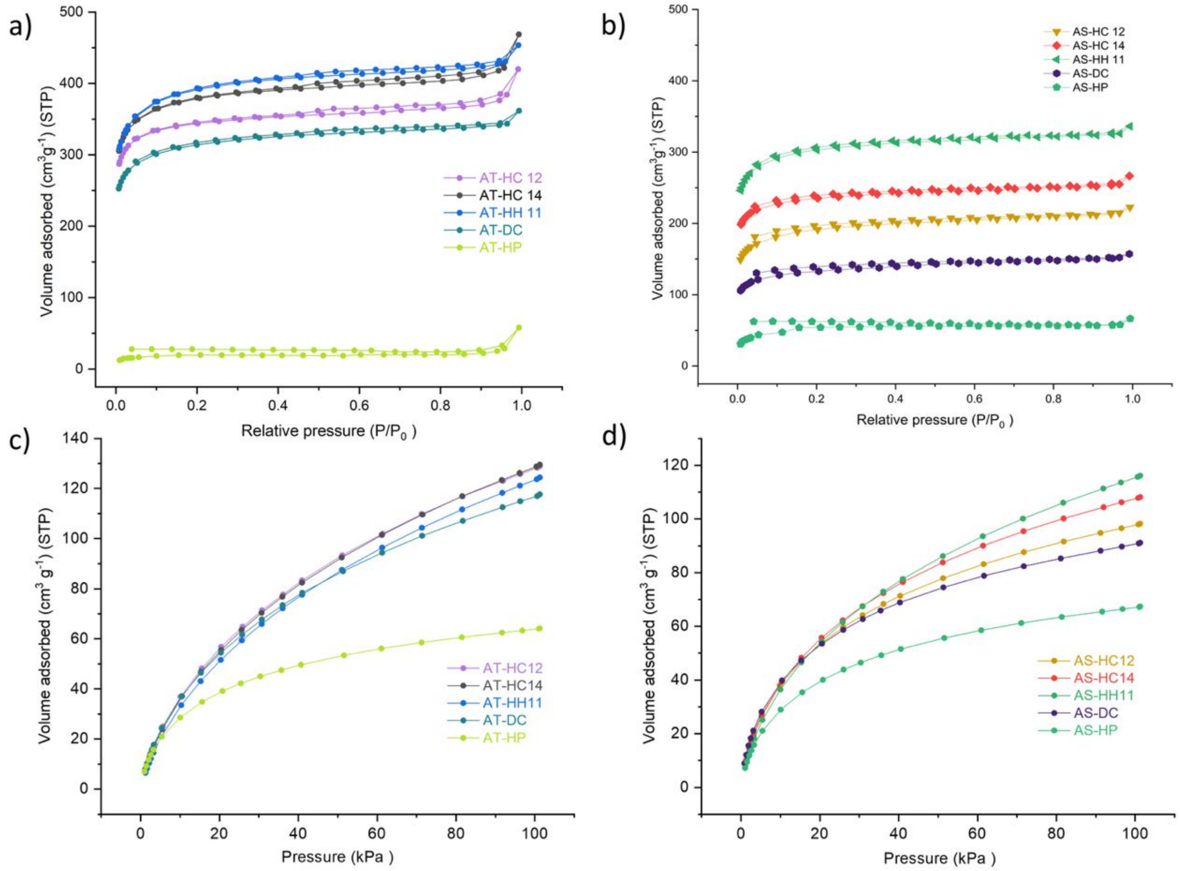

Figure S34. N_2 adsorption isotherms at –196 °C of AT- (a) and AS-derived carbons (b); CO_2 adsorption isotherms at 0 °C of AT- (c) and AS-derived carbons (d).

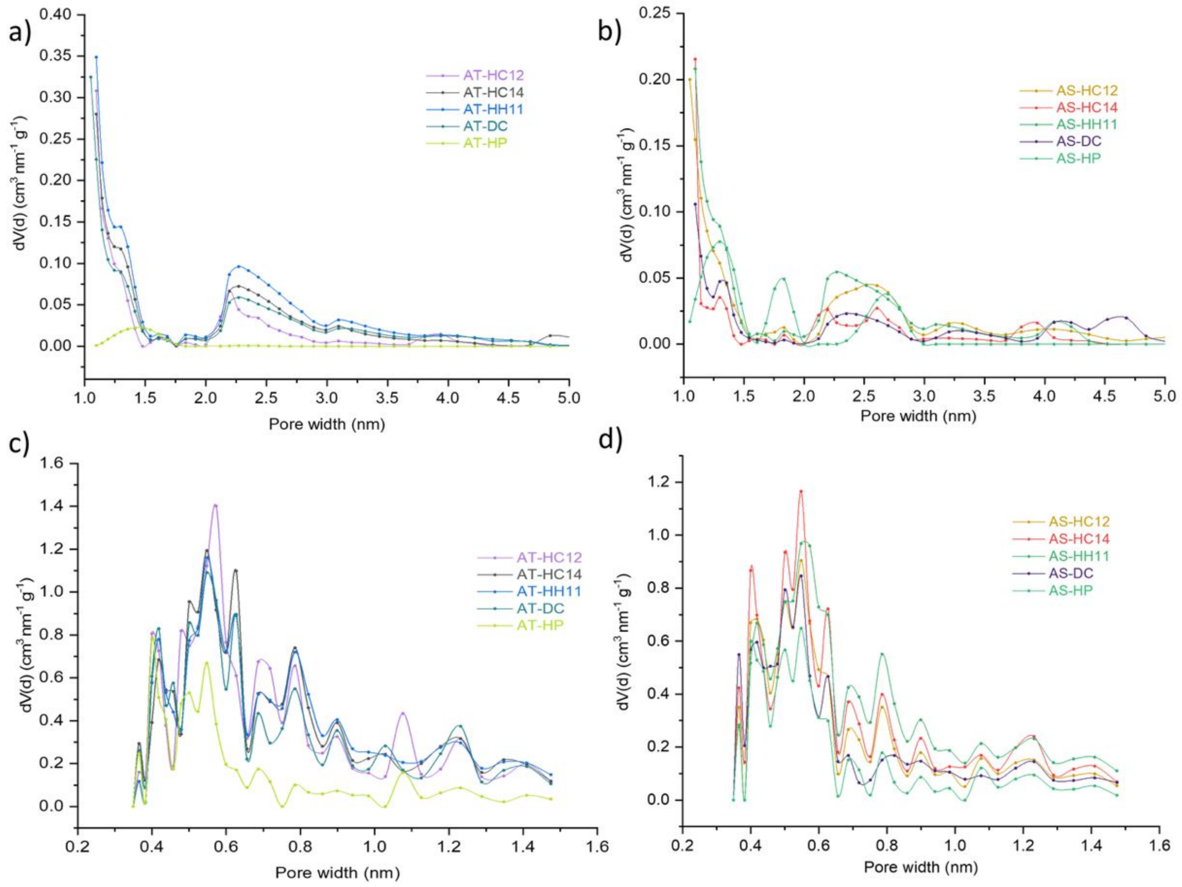

Figure S35. Pore sizes distributions (PSDs) from N_2 adsorption of AT- (a) and AS-derived carbons (b); PSDs from CO_2 adsorption of AT- (c) and AS-derived carbons (d).

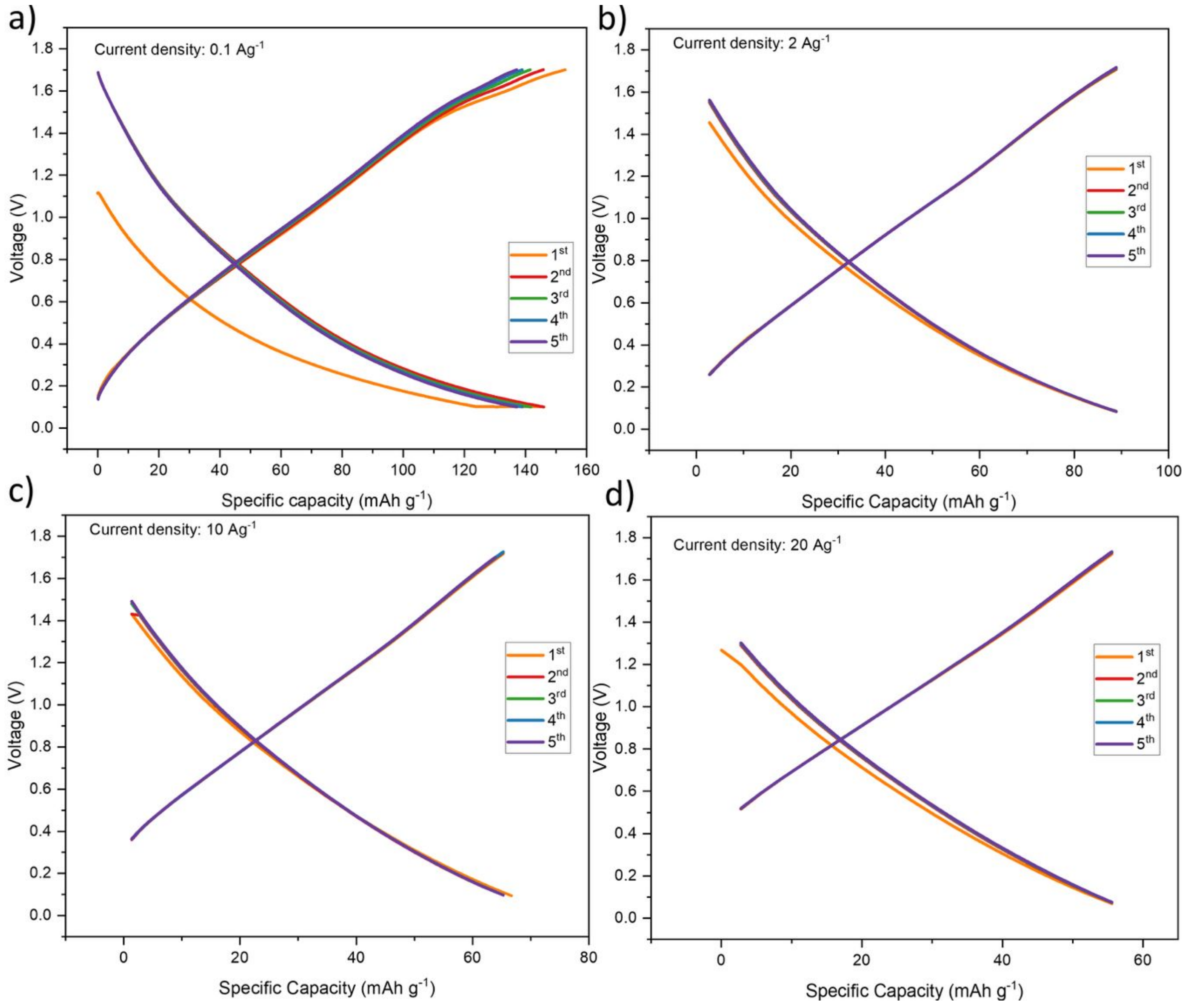

Figure S36. GCD profiles of AT-HC14-CB at 0.1 (a), 2 (b), 10 (c), and 20 A g^{-1} (d).

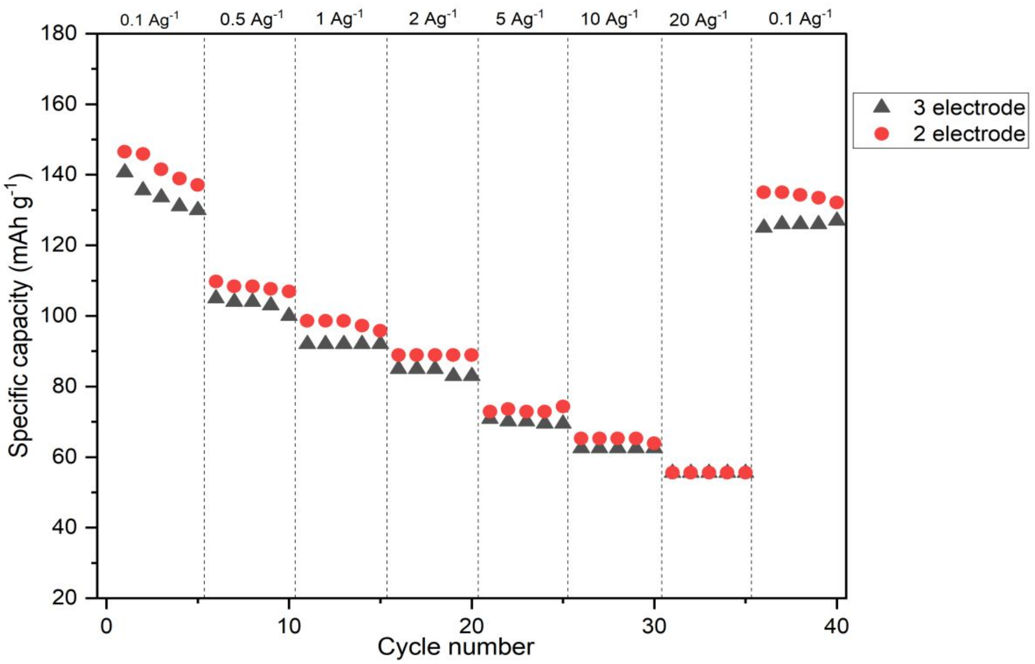

Figure S37. Rate capability tests for AT-HC14-CB cathode in two-electrode and three-electrode setups.

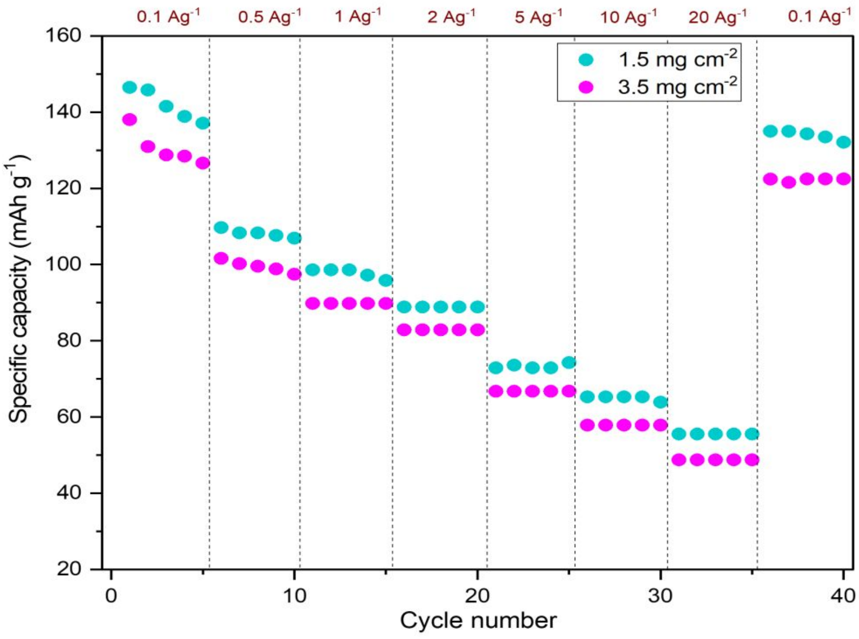

Figure S38. Rate capability tests for AT-HC14-CB cathode for two mass loadings (1.5 and 3.5 mg cm^{-2}).

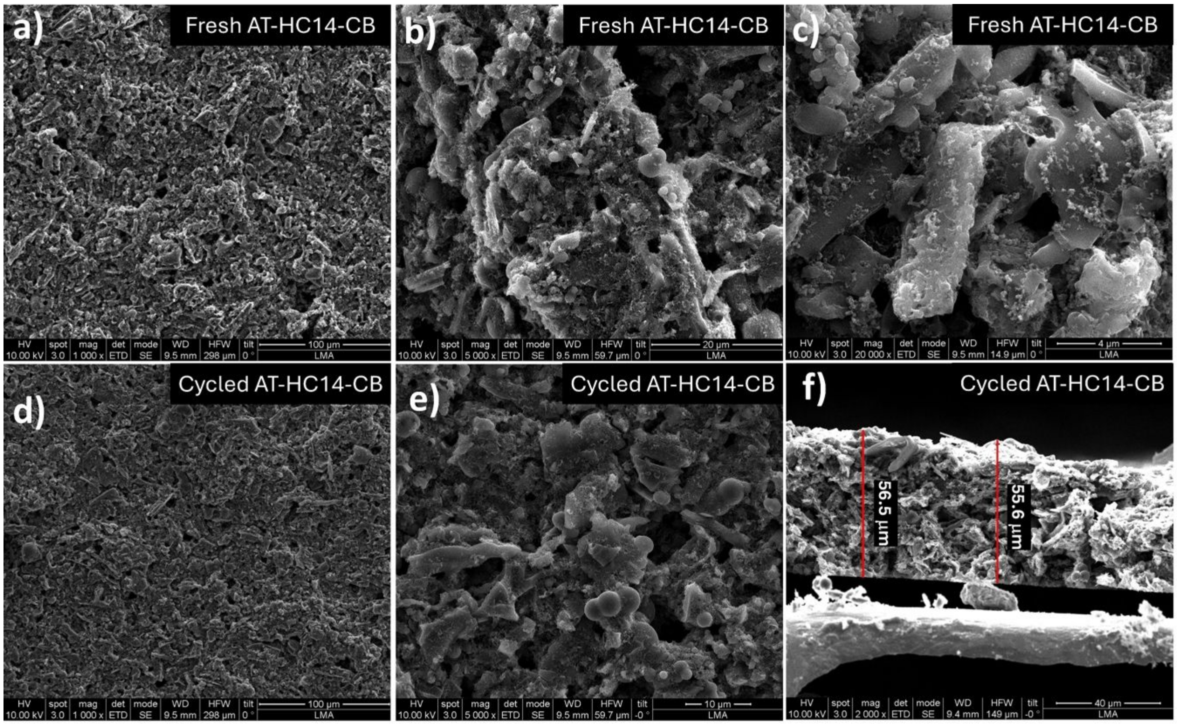

Figure S39. Post-mortem SEM analysis of the AT-HC14-CB cathode after 2000 cycles.

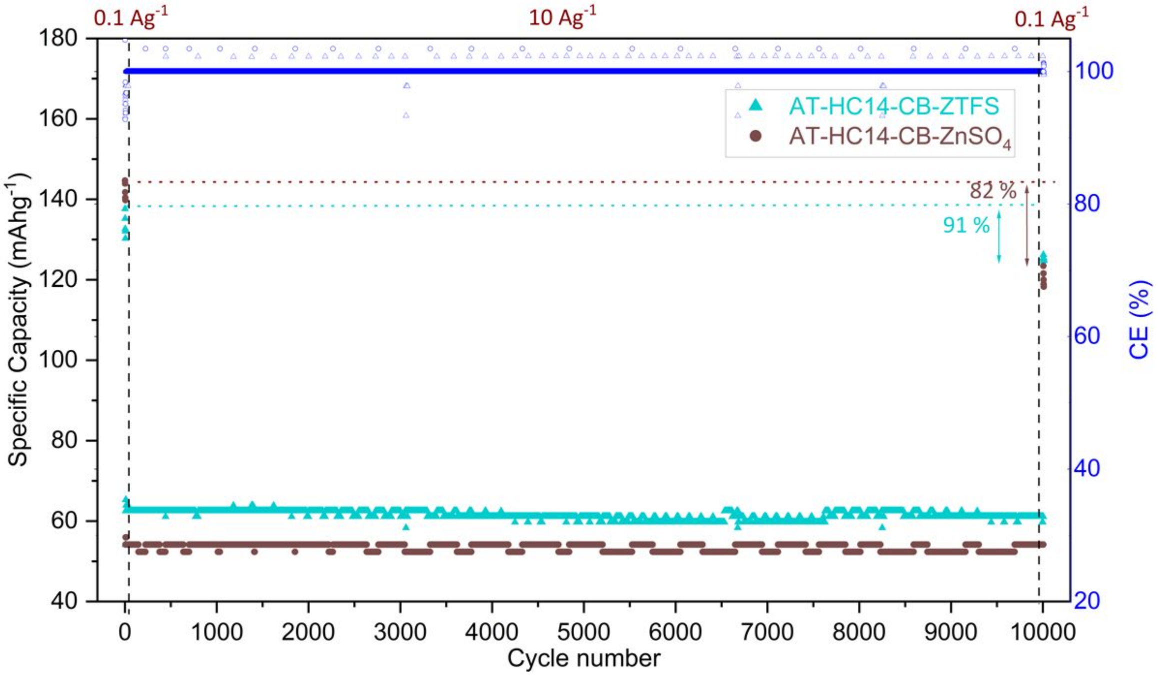

Figure S40.Long-term cycling (10,000 cycles at 10 Ag^{-1}) of AT-HC14-CB in ZTFS and $ZnSO_4$ aqueous electrolytes.

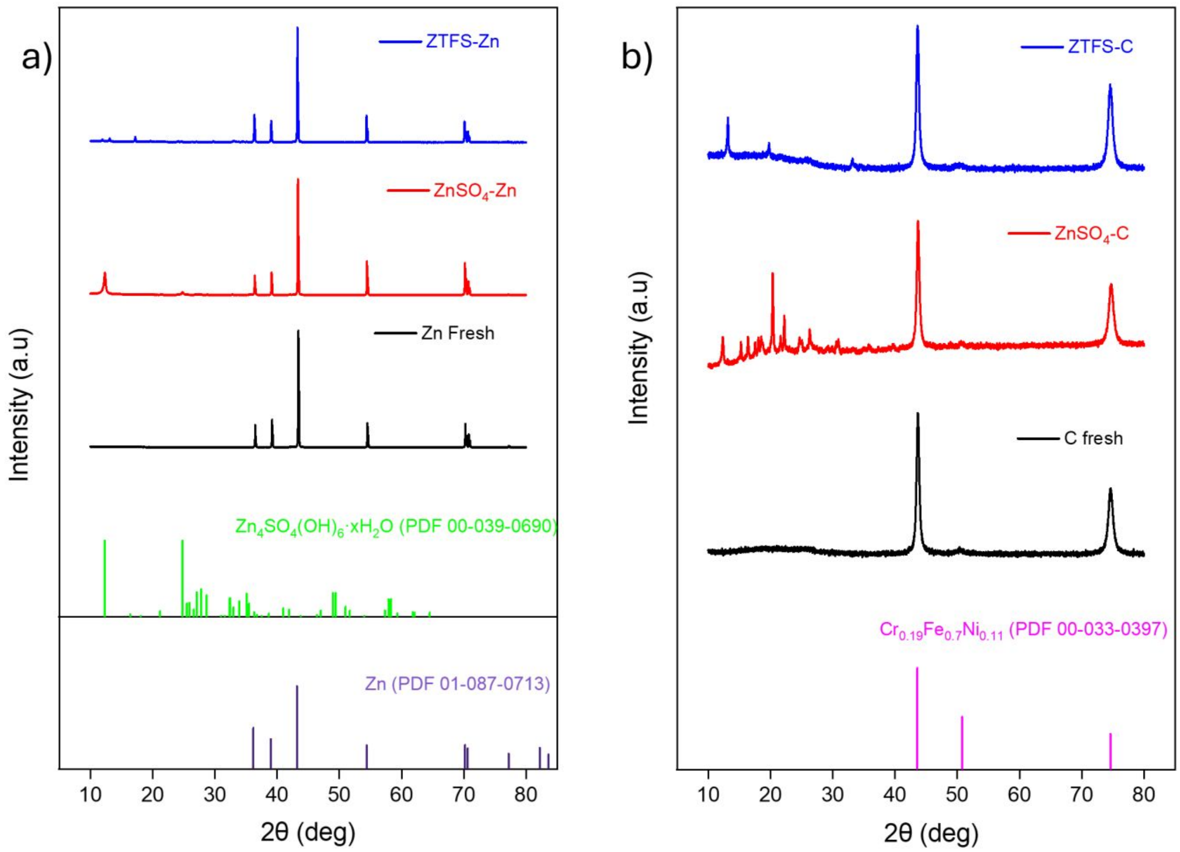

Figure S41. XRD spectra of Zn anodes (a) and AT-HC14-CB-based cathodes (b); both fresh and spent after 10,000 cycles in ZTFS- and $ZnSO_4$-based electrolytes.

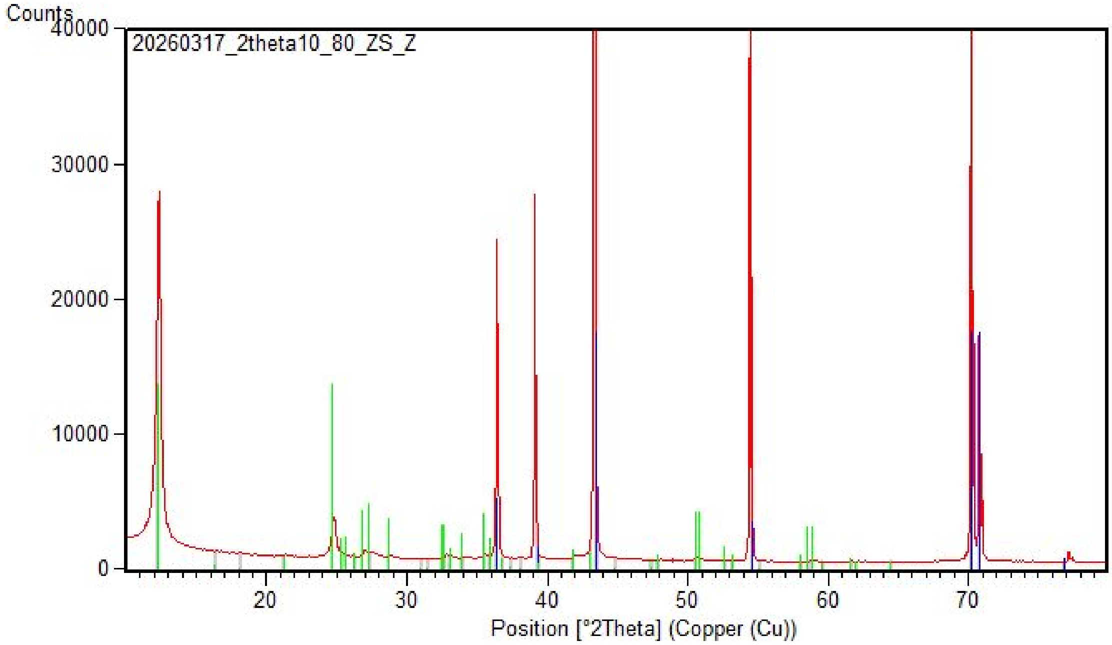

Figure S42. XRD spectrum and peak fitting of zinc anode after 10,000 cycles in the 2 M $ZnSO_4$ electrolyte ($ZnSO_4$-Zn).

Table S7. Table of accepted patterns for peak fitting in $ZnSO_4$-Zn

sample ID	reference code	compound name	chemical formula	score
$ZnSO_4$-Zn	00-001-1244	zinc	Zn	55
	00-039-0690	zinc sulfate hydroxide hydrate	$Zn_4(OH)_6SO_4{\cdot}xH_2O$	4

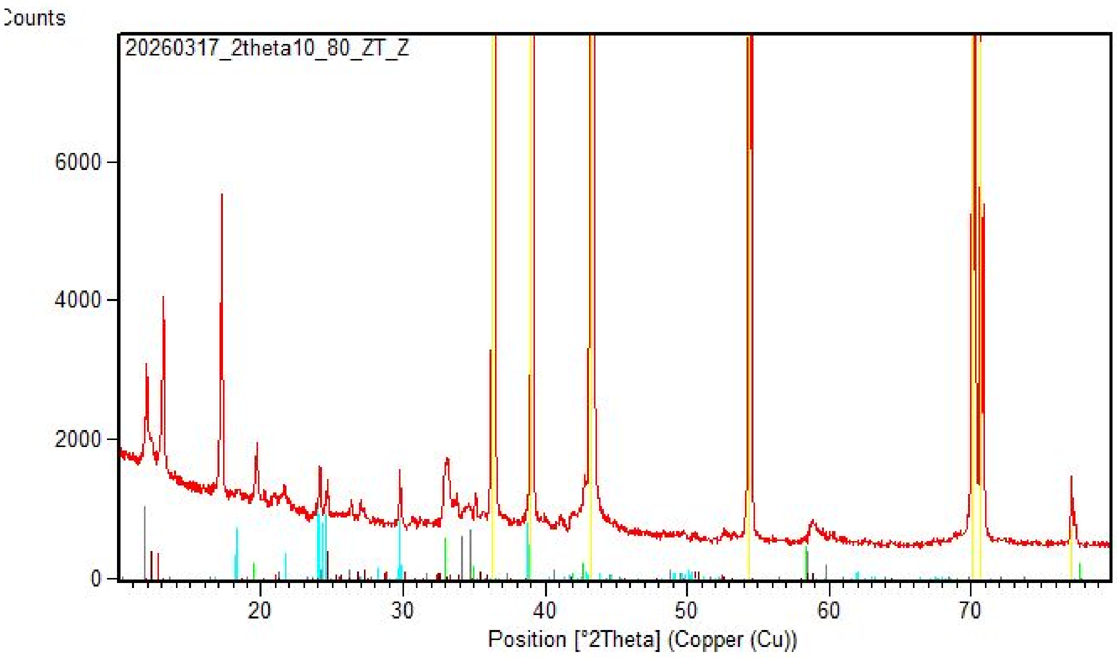

Figure S43. XRD spectrum and peak fitting of zinc anode after 10,000 cycles in the 1 M ZTFS electrolyte (ZTFS-Zn).

Table S8. Table of accepted patterns for peak fitting in ZTFS-Zn

sample ID	reference code	compound name	chemical formula	score
ZTFS-Zn	00-003-0797	zinc oxide sulfate hydrate	$Zn_4O_3(SO_4)\cdot 7H_2O$	24
	00-011-0280	zinc sulfate hydroxide hydrate	$6Zn\ (OH)_{2.}\ ZnSO_4\cdot 4H_2O$	16
	00-039-0690	zinc sulfate hydroxide hydrate	$Zn_4SO_4(OH)_6\cdot xH_2O$	16
	01-085-1785	carbon fluoride	CF_4	30
	01-078-2155	sulfur fluoride	SF_6	21
	00-049-1410	carbon fluoride	C_6F	16
	01-071-1892	zinc fluoride hydrate	$ZnF_2(H_2O)_4$	2
	00-004-0831	zinc	Zn	55
	00-012-0142	zinc hydroxide	$Zn(OH)_2$	10

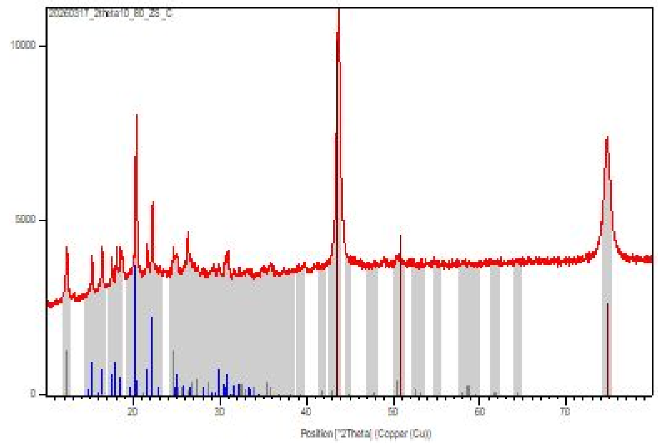

Figure S44. XRD spectrum and peak fitting of AT-HC14-CB cathode after 10,000 cycles in the 2 M $ZnSO_4$ electrolyte ($ZnSO_4$-C).

Table S9. Table of accepted patterns for peak fitting in $ZnSO_4$-C

sample ID	reference code	compound name	chemical formula	score
$ZnSO_4$-C	00-032-1478	zinc sulfate hydrate	$ZnSO_4 \cdot 6\ H_2O$	47
	00-039-0690	zinc sulfate hydroxide hydrate	$Zn_4SO_4(OH)_6 \cdot xH_2O$	12
	00-033-0397	chromium iron nickel	$Cr_{0.19}\ Fe_{0.7}\ Ni_{0.11}$	27

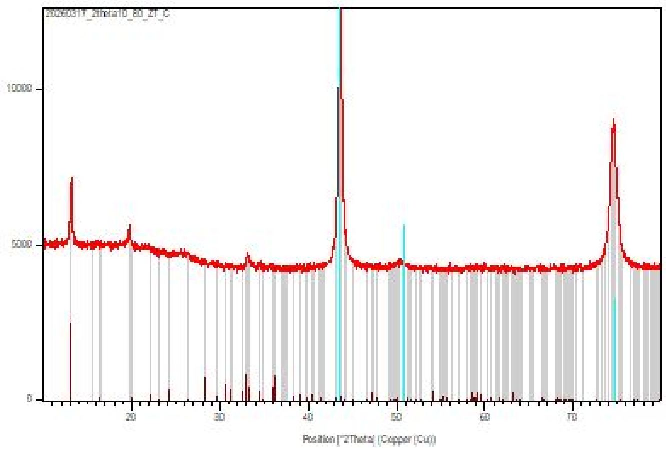

Figure S45. XRD spectrum and peak fitting of AT-HC14-CB cathode after 10,000 cycles in the 1 M ZTFS electrolyte (ZTFS-C).

Table S10. Table of accepted patterns for peak fitting in ZTFS-C

sample ID	reference code	compound name	chemical formula	score
ZTFS-C	01-072-1100	zinc hydroxide carbonate	$Zn_5(OH)_6(CO_3)_2$	5
	00-033-0397	chromium iron nickel	$Cr_{0.19}$ $Fe_{0.7}$ $Ni_{0.11}$	32

Table S11. EDS results for Zn anodes and AT-HC14-CB-based cathodes after 10,000 cycles in ZTFS and $ZnSO_4$ electrolytes

element	$ZnSO_4$-Zn		ZTFS-Zn		$ZnSO_4$-C		ZTFS-C	
	at %	wt %	at %	wt %	at %	wt %	at %	wt %
C	9.5	4.1	17.7	6.0	65.7	46.7	85.5	77.3
O	61.4	34.7	24.8	11.3	23.5	22.4	9.3	11.3
F	–	–	13.6	7.3	–	–	3.5	5.0
Si	0.2	0.2	2.1	1.6	0.1	0.2	–	–
S	4.9	5.5	3.7	3.4	5.3	10.2	0.8	1.9
K	–	–	0.3	0.4	–	–	–	–
Ca	–	–	0.3	0.4	–	–	–	–
Zn	24.0	55.5	37.5	69.6	5.3	20.5	0.9	4.4

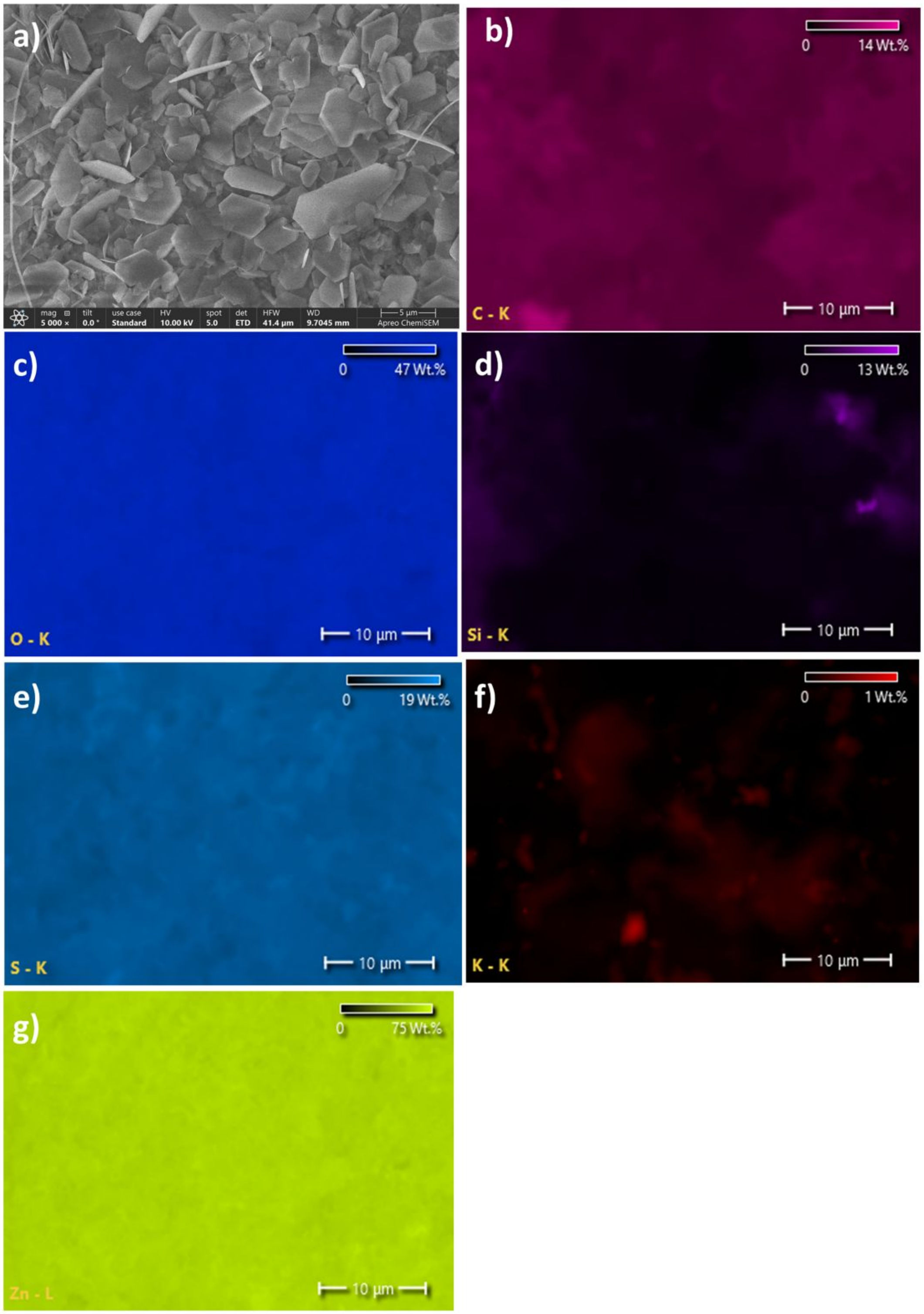

Figure S46. SEM-EDS elemental mapping of $ZnSO_4$-Zn: SEM image (a), and spatial distributions of C (b), O (c), Si (d), S (e), K (f), and Zn (g).

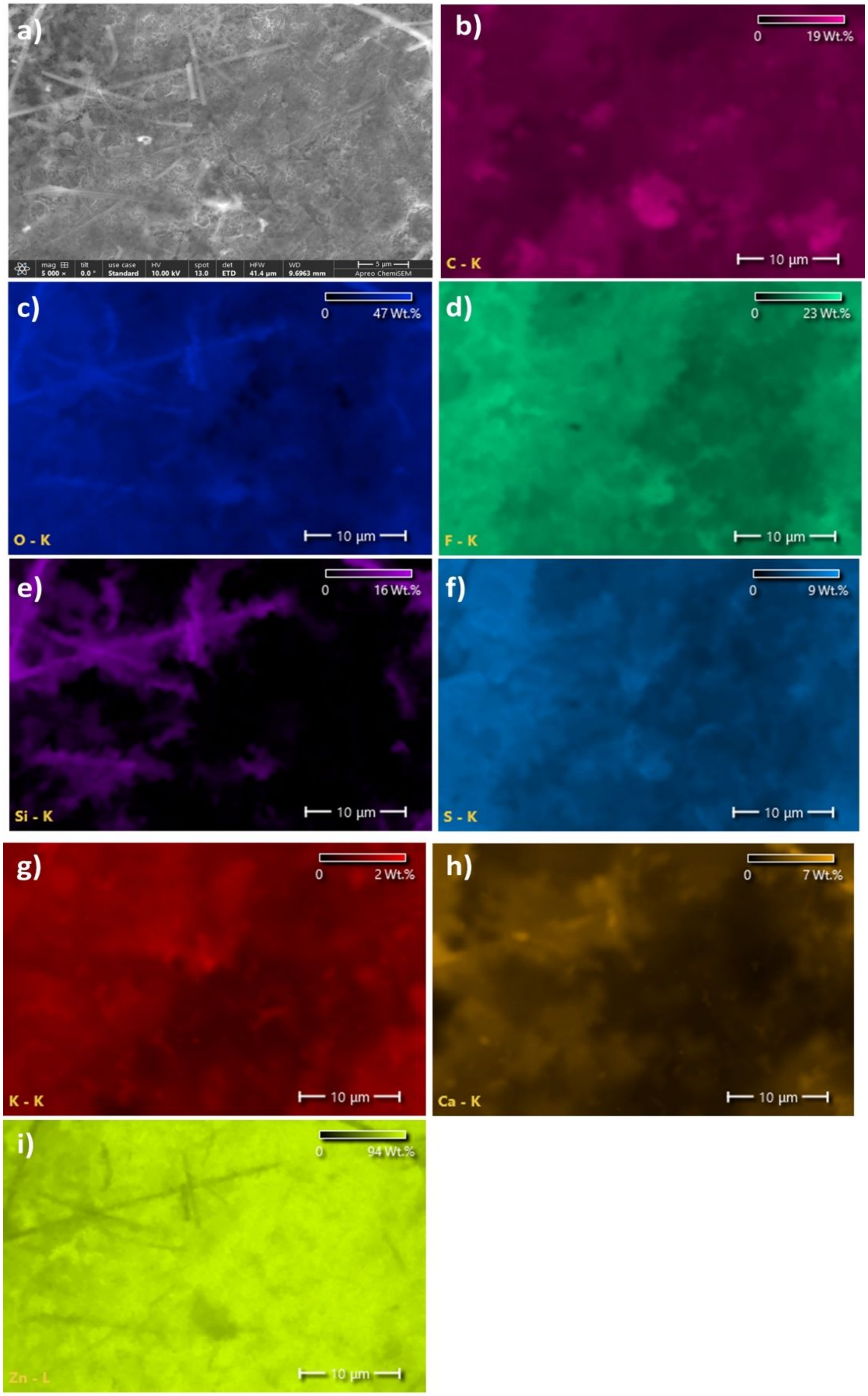

Figure S47. SEM-EDS elemental mapping of ZTFS-Zn: SEM image (a), and spatial distributions of C (b), O (c), F (d), Si (e), S (f), K(g), Ca (h), and Zn (i).

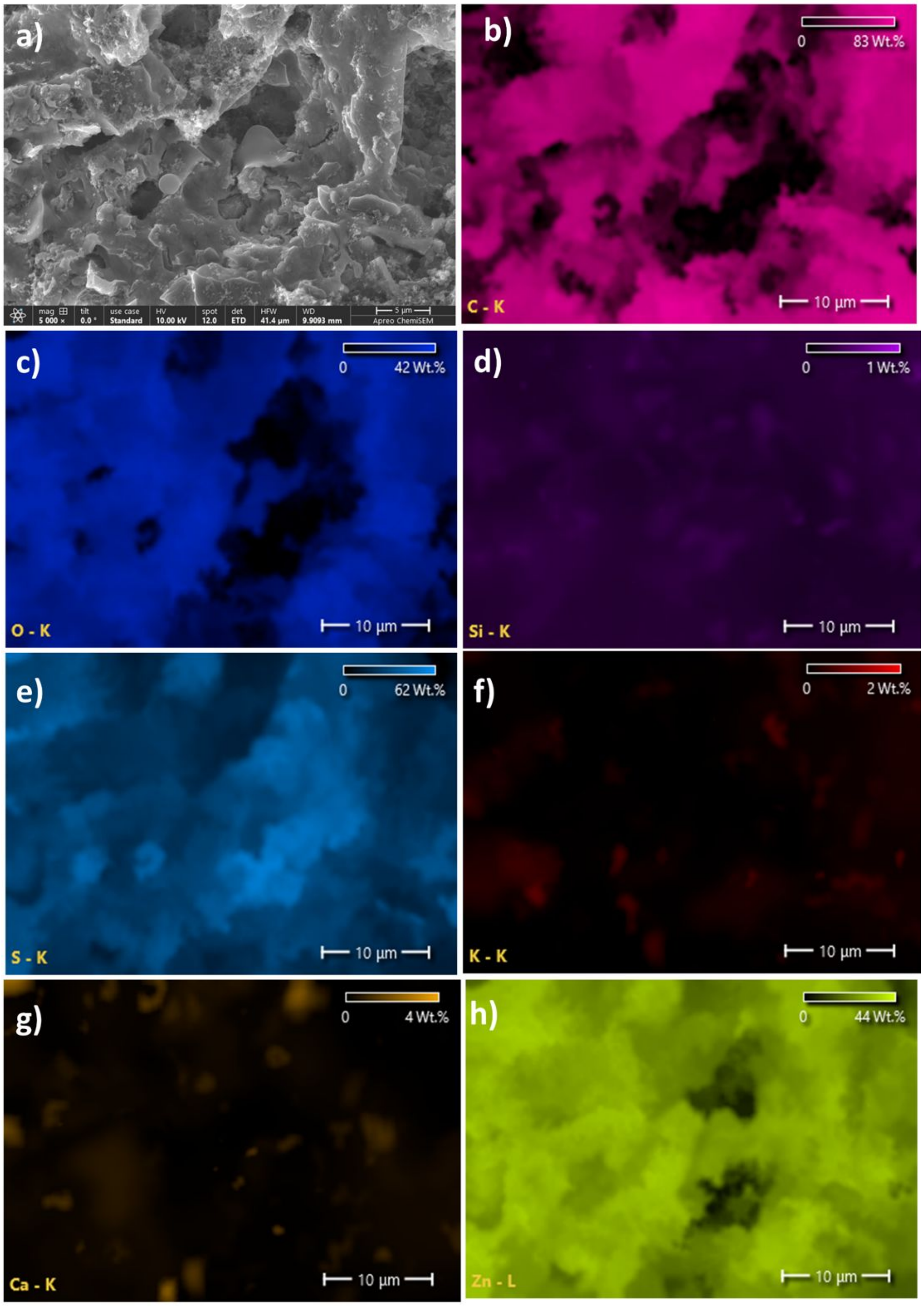

Figure S48. SEM-EDS elemental mapping of $ZnSO_4$-C: SEM image (a), and spatial distributions of C (b), O (c), Si (d), S (e), K (f), Ca (g), and Zn (h).

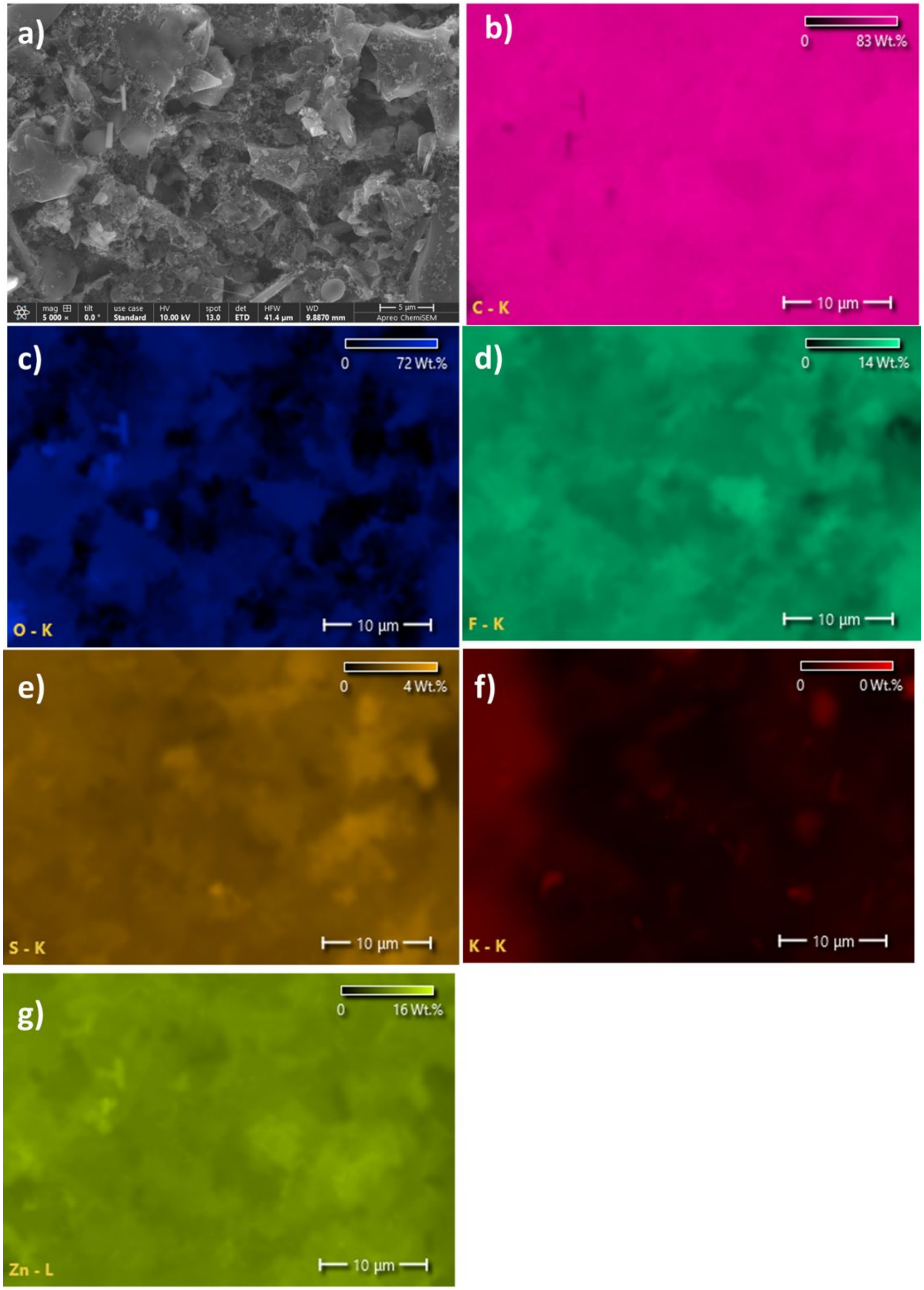

Figure S49. SEM-EDS elemental mapping of ZTFS-C: SEM image (a), and spatial distributions of C (b), O (c), F (d), S (e), K (f), and Zn (g).

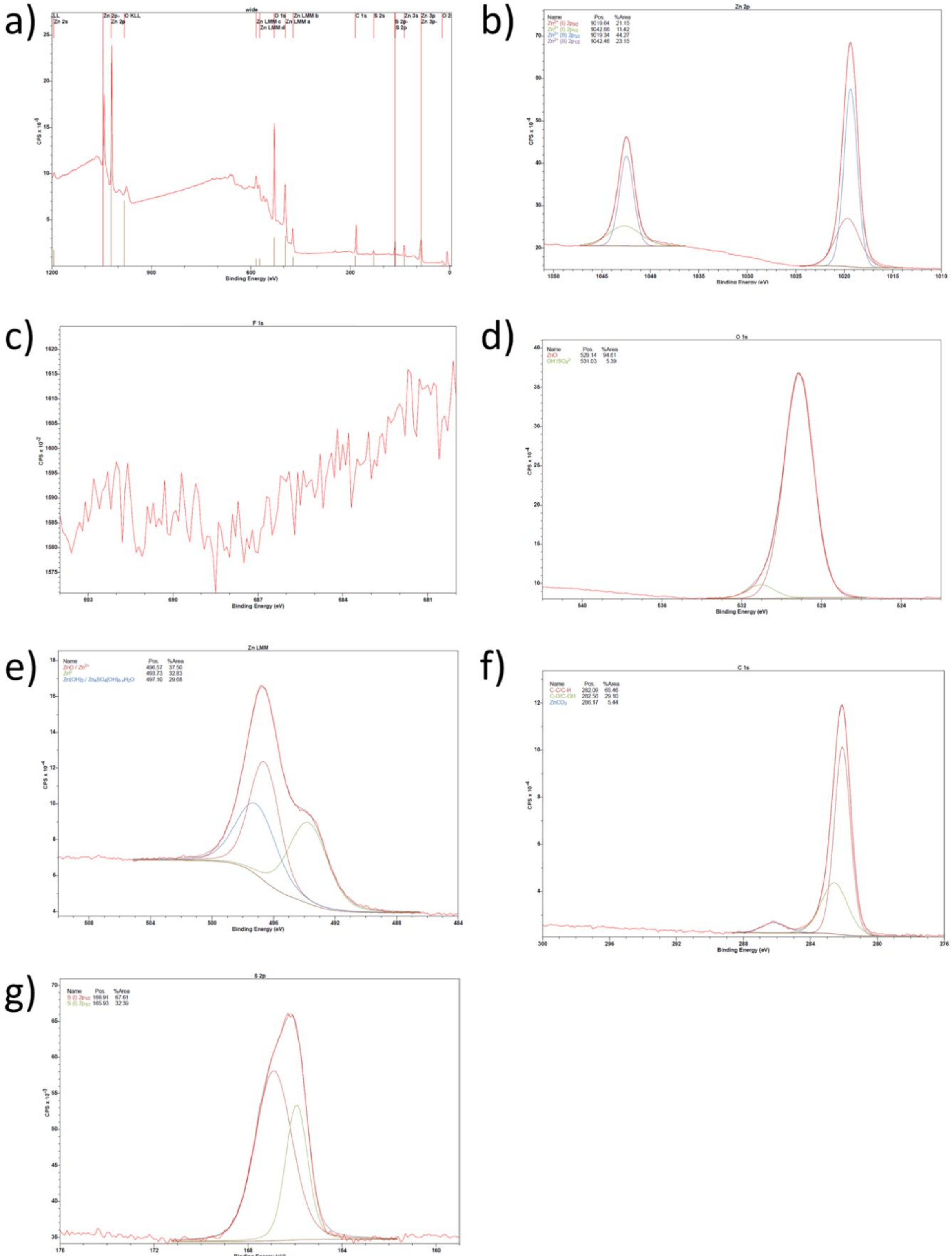

Figure S50. XPS spectrum of $ZnSO_4$-Zn: survey (a), Zn 2p (b), F 1s (c), O 1s (d), Zn LMM (e), C 1s (f), and S 2p (g).

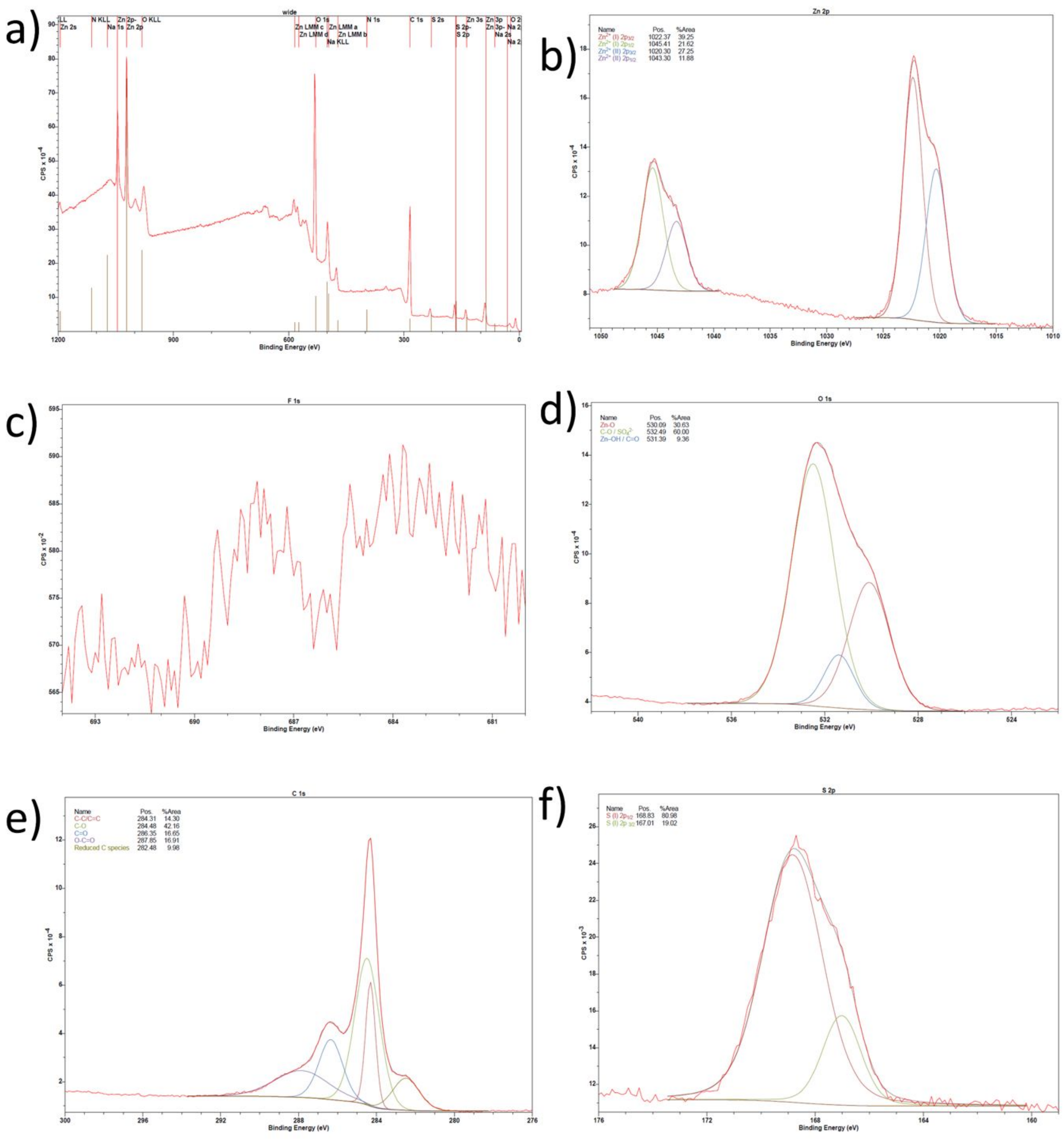

Figure S51. XPS spectrum of $ZnSO_4$-C: survey (a), Zn 2p (b), F 1s (c), O 1s (d), C 1s (e), and S 2p (f).

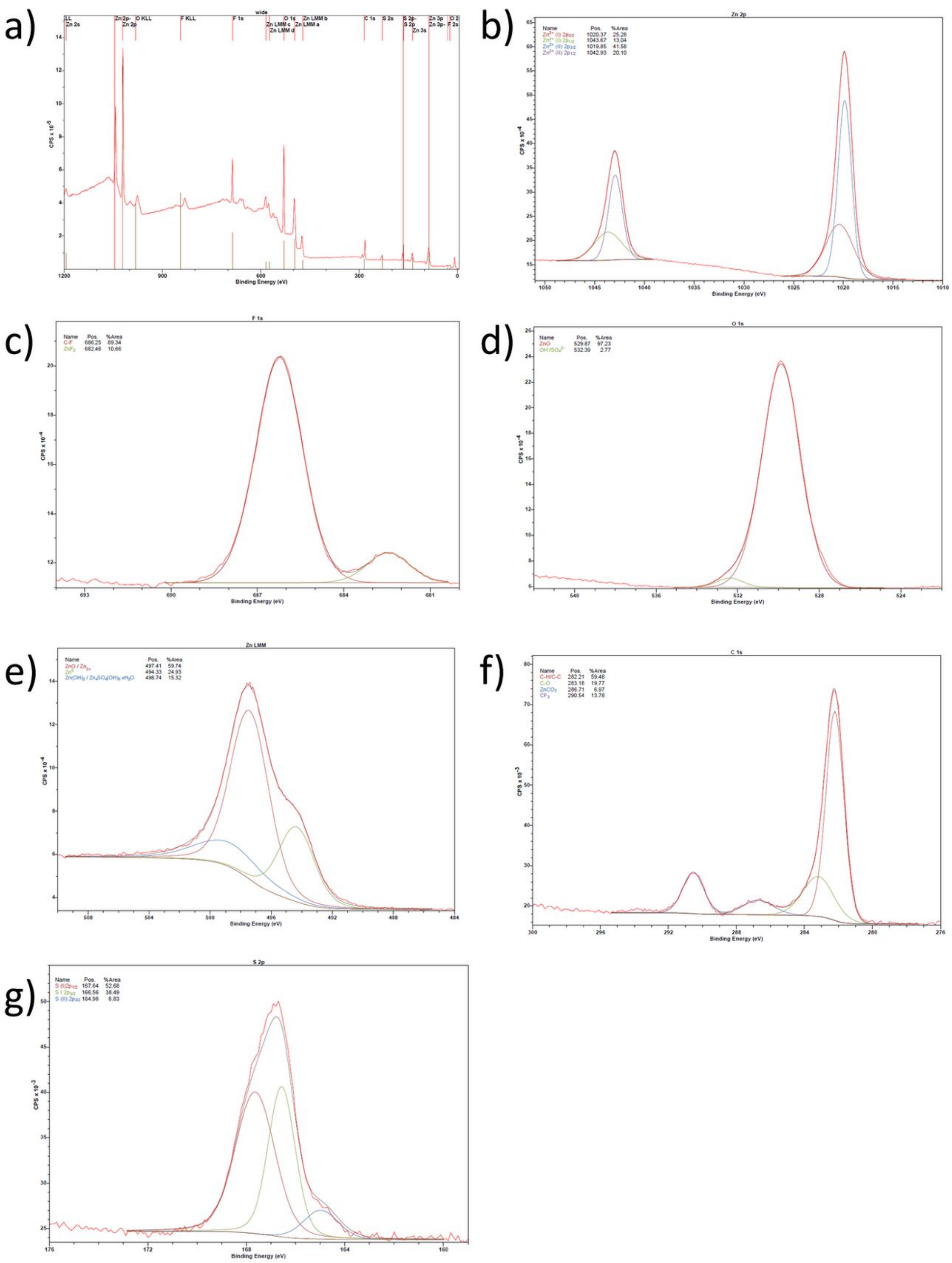

Figure S52. XPS spectrum of ZTFS-Zn: survey (a), Zn 2p (b), F 1s (c), O 1s (d), Zn LMM (e), C 1s (f), and S 2p (g).

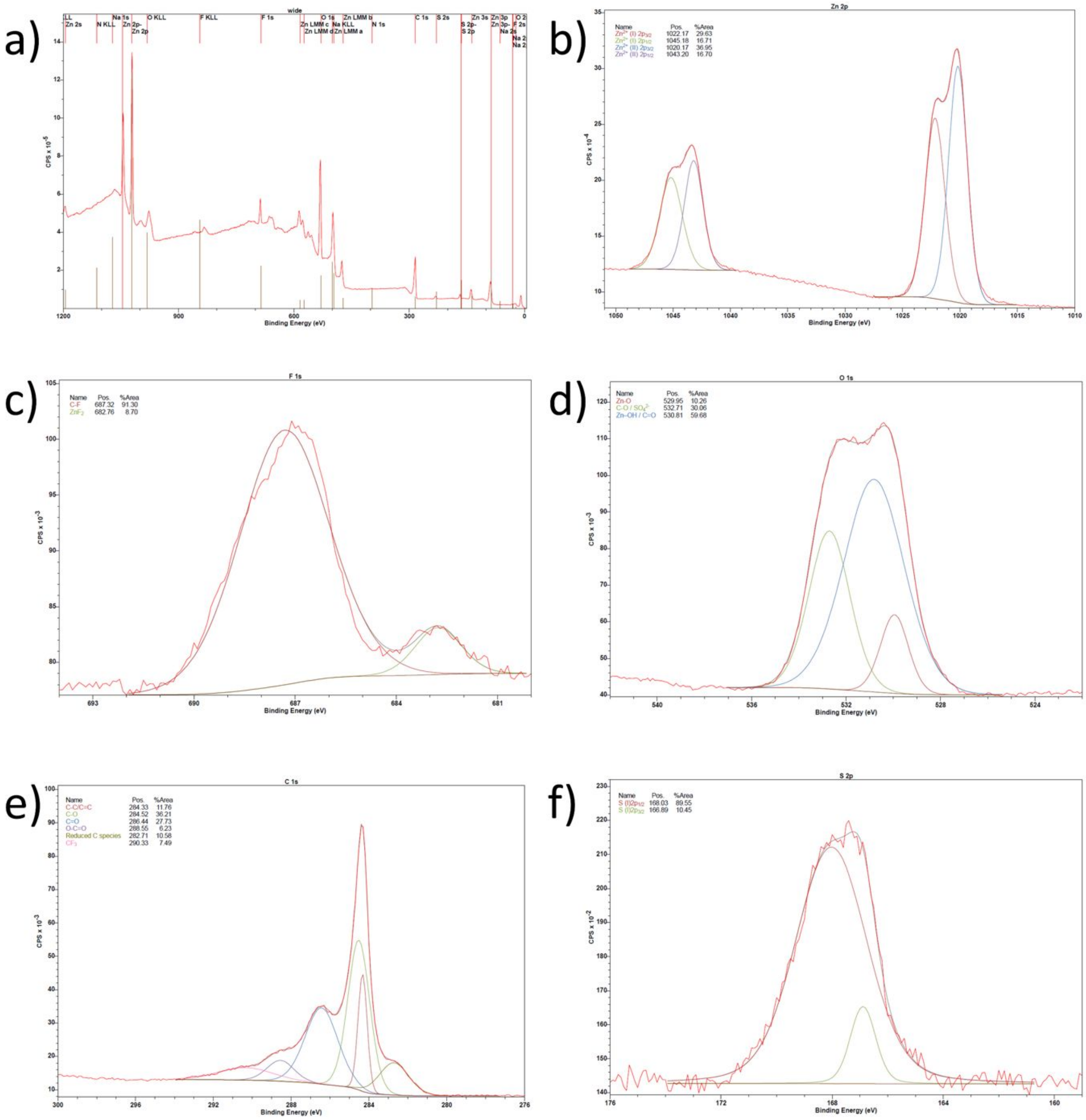

Figure S53. XPS spectrum of ZTFS-C: survey (a), Zn 2p (b), F 1s (c), O 1s (d), C 1s (e), and S 2p (f).

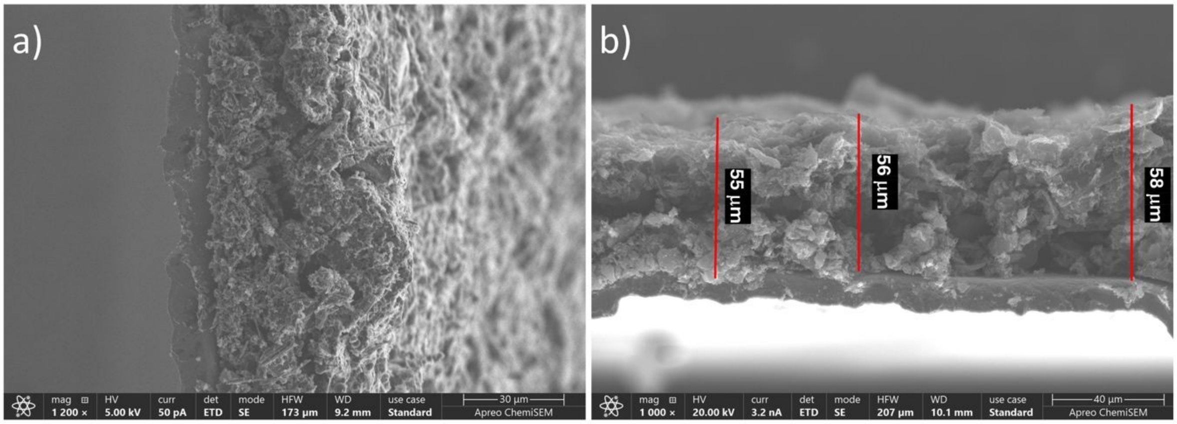

Figure S54. Cross-sectional SEM images of AT-HC14-CB cathode in ZTFS after 10,000 cycles.

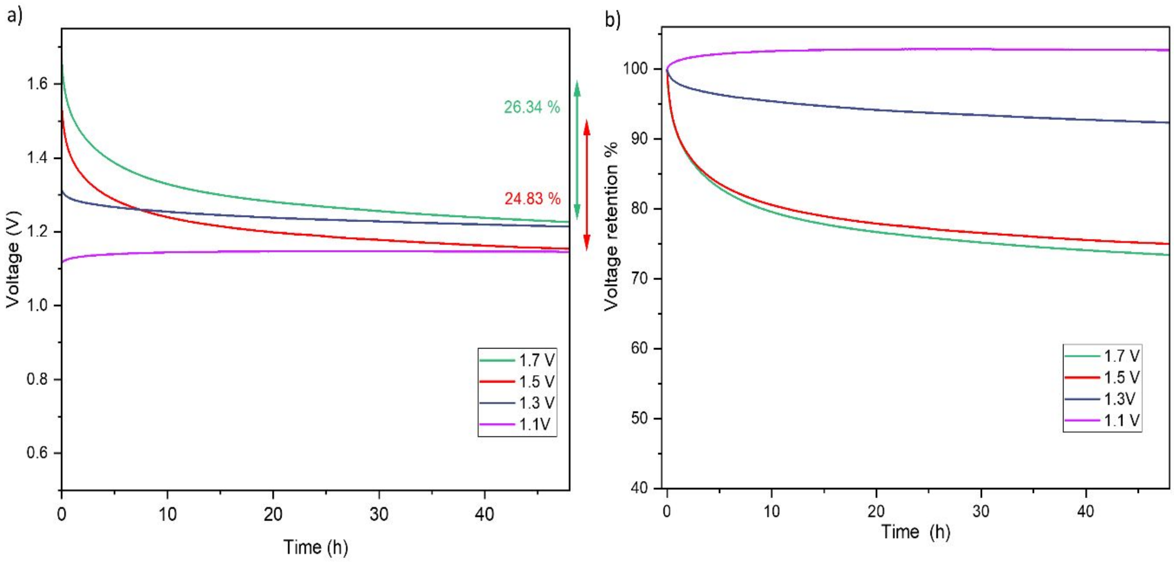

Figure S55. Self-discharge behaviour of the AT-HC14-CB cathode (a) and voltage retention plot (b).

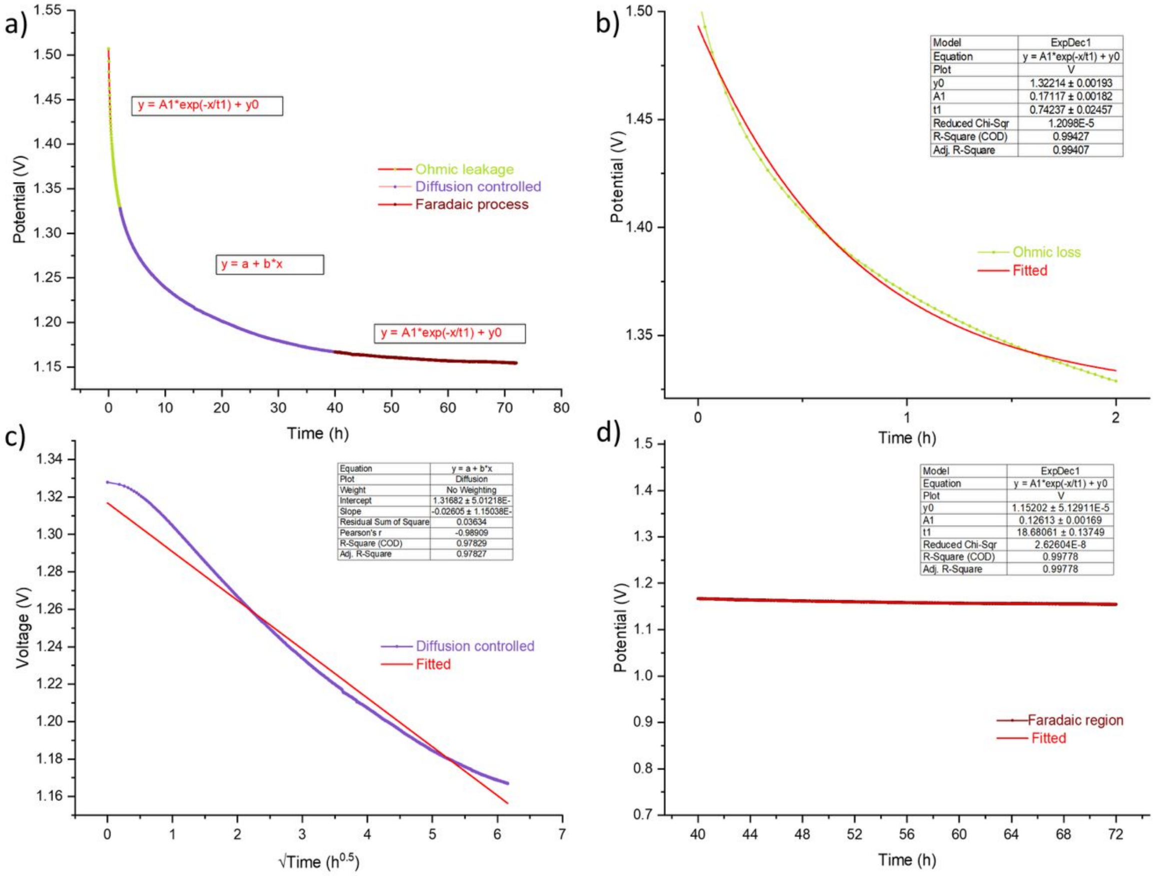

Figure S56. Fitting of self-discharge behaviour of the AT-HC14-CB cathode at 1.5 V for 72 h based on a mixed mechanism (a), and the corresponding fitting of ohmic region (b), diffusion-related region (c), and faradaic region (d).

Table S12. EIS equivalent-circuit fitting parameters for AT-HC12-CB, AT-HC14-CB, and AT-HH11-CB [circuit: R_s-$p(CPE_1,R_1)$-p(CPE_2,R_2-Wo)].

element	AT-HC12-CB	AT-HC14-CB	AT-HH11-CB
R_s (Ω)	1.69	1.53	2.81
Q_1 (F $s^{\alpha-1}$)	1.33×10^{-2}	6.86×10^{-4}	6.37×10^{-3}
α_1 (–)	0.513	0.683	0.491
R_1 (Ω)	783	165	412
Q_2 (F $s^{\alpha-1}$)	5.34×10^{-4}	9.61×10^{-3}	1.94×10^{-4}
α_2 (–)	0.690	0.559	0.693
R_2 (Ω)	172	797	246
Z_0 (Ω $s^{-1/2}$)	66.5	≈0	37.9
τ (s)	6.51	84.7	2.26
RMSE (Ω)	5.82	7.38	18.1
rRMSE (–)	0.0336	0.0430	0.0723
Kramers-Konig (lin-KK) validation	AT-HC12-CB	AT-HC14-CB	AT-HH11-CB
M	24	19	16
μ	0.86	0.89	0.89
$RMSE_{KK}$ (–)	0.032	0.027	0.056

Table S13. Overview of biomass-derived carbons for zinc-ion hybrid supercapacitors (electrochemical metrics are normalized to the mass of the cathode active material)

biomass	synthesis protocol	surface area (m^2 g^{-1})[a]	specific capacity or capacitance (current density)	energy (Wh kg^{-1}) and power (W kg^{-1}) densities[b]	reference
hemp tow	H_2SO_4-assisted HTC at 210 ℃ for 40 h, then pyrolysis at 700 ℃, then KOH activation at 600 ℃	1300	220 F g^{-1} (1 mA cm^{-2})	65 / 151	Tekin and Topcu (2024)[3]
rice husk	LiCl-assisted HTC at 220 ℃ for 24 h, then desilication with 1 M KOH at 95 ℃, then $ZnCl_2$ activation at 600 ℃	971	172.5 F g^{-1} (0.5 A g^{-1})	78 / 450	Feng at al.(2025)[4]
ground nutshell	pre-carbonization at 350 ℃ for 2h, then H_3PO_4-wet mixing activation at 800 ℃	617	146 F g^{-1} (0.1 A g^{-1})	50 / 100	Gautam et al. (2024)[5]
starch	KOH plus KNO_3 wet mixing, then pyrolysis at 800 ℃ for 1h	2672	149 mAh g^{-1} (0.2 A g^{-1})	60 / 15976	Wang et al. (2020)[6]
coconut shell	pre-carbonization at 500 ℃ for 1h in Ar, then KOH activation at 800 ℃	3384	170 F g^{-1} (0.1 A g^{-1})	53 / 1725	Wang et al. (2018)[7]
jute stick	KOH wet mixing at 1:4 mass ratio, then activation at 700 ℃ for 5 h	1370	204 F g^{-1} (0.5 A g^{-1})	73 / 400	Mohamed et al. (2024)[8]
peanut red peel	carbonization at 600 for 2h, then KOH activation at 800 ℃ for 2 h	2072	86 mAh g^{-1} (0.1 A g^{-1})	66 / 218	Sun et al. (2023)[9]
orange peel	carbonization at 600 for 2 h, then KOH activation at 850 C for 2 h with1:4 mass ratio	2156	125.7 mAh g^{-1} (1 A g^{-1})	69 / 7570	Yu et al. (2021)[10]
almond tree pruning	HTC (H_2O) at 185 ℃ for 12 h, then K_2CO_3 activation (1:4 mass ratio) at 800 ℃ for 2 h	1461	142 mAh g^{-1} (0.1 A g^{-1})	88–38 / 62–13600	this work

[a] BET surface area estimated from N_2 adsorption isotherm.

[b] Calculated based on mass of cathode active material.

References

(1) Arellano, O.; Flores, M.; Guerra, J.; et al. Hydrotermal Carbonization (HTC) of Corncob and Characterization of the Obtained Hydrochar. *Chem. Eng. Trans.* **2016**, *50*, 235–240.

(2) Suhas; Chaudhary, M.; Chaudhary, S.; et al. Transforming Biomass Waste into Hydrochars and Porous Activated Carbon: A Characterization Study. *Resources* **2025**, *14* (3), 34.

(3) Tekin, B.; Topcu, Y. Novel Hemp Biomass-Derived Activated Carbon as Cathode Material for Aqueous Zinc-Ion Hybrid Supercapacitors: Synthesis, Characterization, and Electrochemical Performance. *J. Energy Storage* **2024**, *77*, 109879.

(4) Feng, D.; Wang, Y.; Li, J.; et al. Rice Husk-Derived Mesoporous Carbons via Pore-Tailoring as High-Performance Electrode Materials of Zinc-Ion Hybrid Supercapacitors. *Mater. Today Commun.* **2025**, *46*, 112937.

(5) Gautam, M.; Patodia, T.; Kushwaha, P.; et al. Evaluation of Zinc-Ion Hybrid Super-Capacitor Based on Chemically Activated (KOH/H3PO4) Ground Nutshell Biochar. *Carbon Trends* **2024**, *15*, 100341.

(6) Wang, D.; Pan, Z.; Lu, Z. From Starch to Porous Carbon Nanosheets: Promising Cathodes for High-Performance Aqueous Zn-Ion Hybrid Supercapacitors. *Microporous Mesoporous Mater.* **2020**, *306*, 110445.

(7) Wang, H.; Wang, M.; Tang, Y. A Novel Zinc-Ion Hybrid Supercapacitor for Long-Life and Low-Cost Energy Storage Applications. *Energy Storage Mater.* **2018**, *13*, 1–7.

(8) Mohamed, M. M.; Shah, S. S.; Hakeem, A. S.; et al. A Comprehensive Evaluation of Biomass-Derived Activated Carbon Materials for Electrochemical Applications in Zinc-Ion Hybrid Supercapacitors. *ACS Appl. Energy Mater.* **2024**, *7* (17), 7517–7533.

(9) Sun, Z.; Jiao, X.; Chu, S.; Li, Z. Low-cost Porous Carbon Materials Prepared from Peanut Red Peels for Novel Zinc-ion Hybrid Capacitors. *Chemistry Select* **2023**, *8* (47), e202304071.

(10) Yu, J.; Wang, L.; Peng, J.; et al. O-Doped Porous Carbon Derived from Biomass Waste for High-Performance Zinc-Ion Hybrid Supercapacitors. *Ionics* **2021**, *27* (10), 4495–4505.
